# First-in-class multifunctional TYMS nonclassical antifolate inhibitor with potent in vivo activity that prolongs survival

**DOI:** 10.1172/jci.insight.158798

**Published:** 2023-05-22

**Authors:** Maria V. Guijarro, Patrick C. Kellish, Peter E. Dib, Nicholas G. Paciaroni, Akbar Nawab, Jacob Andring, Lidia Kulemina, Nicholas V. Borrero, Carlos Modenutti, Michael Feely, Elham Nasri, Robert P. Seifert, Xiaoping Luo, Richard L. Bennett, Daniil Shabashvili, Jonathan D. Licht, Robert McKenna, Adrian Roitberg, Robert W. Huigens, Frederic J. Kaye, Maria Zajac-Kaye

**Affiliations:** 1 Department of Anatomy and Cell Biology,; 2 Department of Medicinal Chemistry, and; 3 Department of Biochemistry and Molecular Biology, University of Florida, Gainesville, Florida, USA.; 4 Department of Biological Chemistry, Faculty of Exact and Natural Sciences, University of Buenos Aires, and; 5 Institute of Biological Chemistry of the Faculty of Exact and Natural Sciences (IQUIBICEN) CONICET, University City, Pab. II (CE1428EHA), Buenos Aires, Argentina.; 6 Department of Pathology, Immunology and Laboratory Medicine, University of Florida College of Medicine, Gainesville, Florida, USA.; 7 Department of Medicine, University of Florida Health Cancer Center, Gainesville, Florida, USA.; 8 Department of Chemistry, University of Florida, Gainesville, Florida, USA.

**Keywords:** Therapeutics, Cancer, Oncogenes

## Abstract

Although thymidylate synthase (TYMS) inhibitors have served as components of chemotherapy regimens, the currently available inhibitors induce TYMS overexpression or alter folate transport/metabolism feedback pathways that tumor cells exploit for drug resistance, limiting overall benefit. Here we report a small molecule TYMS inhibitor that i) exhibited enhanced antitumor activity as compared with current fluoropyrimidines and antifolates without inducing TYMS overexpression, ii) is structurally distinct from classical antifolates, iii) extended survival in both pancreatic xenograft tumor models and an *hTS/Ink4a/Arf* null genetically engineered mouse tumor model, and iv) is well tolerated with equal efficacy using either intraperitoneal or oral administration. Mechanistically, we verify the compound is a multifunctional nonclassical antifolate, and using a series of analogs, we identify structural features allowing direct TYMS inhibition while maintaining the ability to inhibit dihydrofolate reductase. Collectively, this work identifies nonclassical antifolate inhibitors that optimize inhibition of thymidylate biosynthesis with a favorable safety profile, highlighting the potential for enhanced cancer therapy.

## Introduction

Thymidylate synthase (TYMS) is an essential enzyme responsible for the reductive methylation of deoxyuridine monophosphate (dUMP) to deoxythymidine monophosphate (dTMP). This reductive methylation of dUMP requires the cofactor 5,10-methylenetetrahydrofolate (5,10-mTHF) as the methylene and hydride donor. The resulting dTMP is then phosphorylated to deoxythymidine triphosphate (dTTP), an essential precursor for DNA synthesis and repair. Importantly, this TYMS-catalyzed reaction is the sole intracellular source of de novo dTMP (1). Overexpression of TYMS is observed in a wide spectrum of tumor types, and elevated TYMS levels are associated with increased cellular proliferation, tumor invasiveness and metastasis, drug resistance, and poor clinical outcomes (2–11). Additionally, ectopic overexpression of TYMS in murine NIH 3T3 cells exhibits oncogene-like activity by inducing parameters of the neoplastic phenotype, including foci formation, anchor-independent growth, and tumor formation in nude mice (12). Therefore, inhibition of TYMS is an attractive target for intervention, especially given the spectrum of common adult tumor types that would benefit from TYMS inhibition. For these reasons, TYMS has been a target of cancer intervention since the 1950s, and chemotherapy agents such as 5-fluorouracil (5-FU), methotrexate, and pemetrexed are still in use for patients with colon, pancreatic, and non–small cell lung cancer (1, 13). As expected, the inhibition of TYMS results in depletion of dTMP followed by depletion of dTTP, which leads to a state of nucleotide pool imbalance (1, 14). This nucleotide imbalance impairs DNA synthesis and repair, promoting cell cycle arrest, increased DNA damage, and thymine-less death (14, 15).

Catalytically active TYMS is a homodimer where each subunit has an active site that accommodates both the dUMP substrate and the 5,10-mTHF cofactor. The nucleotide (dUMP) and the folate (5,10-mTHF) binding sites provide distinct opportunities to inhibit TYMS, and targeting either site can inhibit TYMS catalytic activity. Classically, nucleotide analogs or folate-based antimetabolites have been used to inhibit TYMS, each with their own distinct limitations.

The most well-known TYMS inhibitor is the fluoropyrimidine antimetabolite 5-FU. As a prodrug, 5-FU is able to passively diffuse into the cell and is intracellularly converted to 5-fluoro-2′-deoxyuridine monophosphate (5-FdUrd), which competes with the dUMP substrate (14). When bound in the nucleotide-binding site in the presence of the 5,10-mTHF cofactor in the folate-binding site, 5-FdUrd will form a covalent complex with TYMS, resulting in irreversible inhibition (16, 17). However, inhibition of TYMS by 5-FU alone or when combined with other cytotoxic agents in the FOLFIRINOX regimen is consistently associated with induction of TYMS overexpression, resulting in acquired drug resistance in patients with pancreatic ductal adenocarcinoma (PDAC) (1, 18). This mechanism for induction of drug resistance is hypothesized to contribute to the inability of 5-FU to induce durable complete remissions or cures in patients with locally advanced or metastatic disease (19). Therefore, our primary goal was to identify a TYMS inhibitor with a potent antitumor effect that addresses the recurrent clinical challenge of drug resistance observed with current TYMS inhibitors such as 5-FU.

It has been more than 70 years since the first folate antimetabolite, aminopterin, was used in the early treatment of childhood lymphocytic leukemia (20). Prototypic antifolates are analogs of folic acid that compete with the 5,10-mTHF binding to directly inhibit TYMS (21). Antifolates also indirectly block the TYMS-catalyzed conversion of dUMP to dTMP by inhibiting dihydrofolate reductase (DHFR) (1, 22, 23). DHFR is responsible for the reduction of DHF to THF, a critical first step in regenerating 5,10-mTHF. For example, methotrexate, a more selective antifolate DHFR inhibitor, suppresses TYMS via depletion of 5,10-mTHF levels (1, 22). As a result of the structural similarities to folic acid, classical antifolates are dependent on different folate metabolic pathways to be effective, presenting multiple pathways and feedback loops that cancer cells can use during the development of drug resistance (21, 24–28). For example, cellular uptake of drug is dependent upon folate transporters such as the reduced folate carrier and the proton-coupled folate transporter (24, 25). Accordingly, impaired transport function affecting cellular uptake is one tactic exploited by cancer cells to yield resistance to classical antifolates (27, 29, 30). Once within the cell, antifolates must undergo polyglutamation by the enzyme folylpolyglutamate synthetase (FPGS), which increases cellular retention and efficiency against the target enzymes (25–27, 31, 32). Thus, impaired polyglutamation also results in antifolate resistance.

Here we report the development of TYMS inhibitors that are structurally distinct from folic acid and other classical antifolates that require specific transporters or intracellular conversion/metabolism. These potentially novel compounds directly inhibited TYMS without inducing the TYMS overexpression associated with drug resistance. Using a library of analogs based on the lead TYMS inhibitor scaffold (19-S), we determined the structural features associated with direct TYMS inhibition while maintaining the ability to inhibit DHFR. These features, therefore, allowed for both direct TYMS inhibition and indirect inhibition of thymidine synthesis by preventing regeneration of the required 5,10-mTHF cofactor. The multifunctional nonclassical antifolates were well tolerated when administered either by intraperitoneal (IP) injection or oral gavage (per os; PO) and were effective at inhibiting tumor progression and extending survival in mouse pancreatic cancer xenograft tumor models as well as our potentially novel *hTS/Ink4a/Arf* null genetically engineered mouse model (GEMM).

## Results

To identify potential TYMS small molecule inhibitors, we first used molecular docking to computationally screen compounds with the ability to bind TYMS. The details of the procedure are presented in the Methods section. The top ranked 26 compounds were acquired from the NIH National Cancer Institute and designated 1-A through 26-Z, then used to determine the cytotoxicity at 10 μM concentration in several cancer cell lines from different tumor subtypes. Compound 19-S (NSC 382035) exhibited cytotoxicity in all 5 tumor cell lines examined: small cell lung cancer cell line H1048, pancreatic neuroendocrine cell line CM, and PDAC cells PANC-1, MIA PaCa-2, and Luc-PANC-1 (Supplemental Figure 1A; supplemental material available online with this article; https://doi.org/10.1172/jci.insight.158798DS1). The initial computational screen provided a predictive model that enabled a preliminary assessment for possible drug candidates and presented 19-S as a promising lead candidate for further study. We subsequently pursued experiments to validate biological activity and to define mechanisms for TYMS inhibition.

### Small molecule compound inhibits TYMS catalytic activity, shows cytotoxicity in vitro, and does not increase TYMS levels.

**Figure 1:**
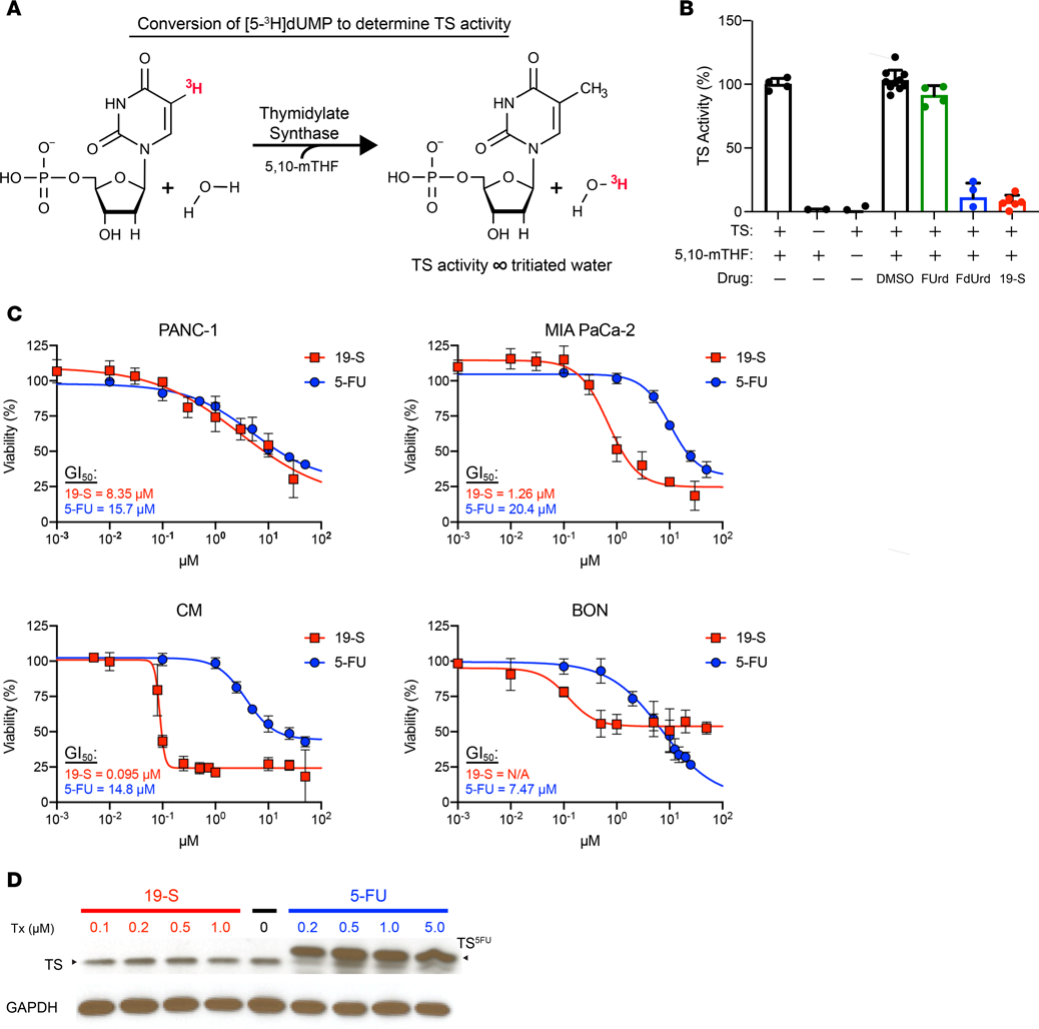
Compound 19-S inhibits TYMS catalytic activity, shows cytotoxicity in vitro, and does not increase TYMS levels. (**A**) Diagram illustrating the TYMS (TS) tritium assay and conversion of tritiated dUMP to dTMP, generating tritiated water for quantification of TS activity. (**B**) TS tritium assay showing TS activity for control reactions and reactions performed in the presence of 5-fluorouridine (FUrd), 5-fluoro-2′-deoxyuridine (FdUrd), and compound 19-S. Drug concentrations were 250 μM using 10 μg/mL bacterially expressed TS protein. Mean ± SD of 4 data points from 2 independent experiments shown. The DMSO control represents 10 data points. Compound 19-S treatment represents 6 data points. (**C**) Viability assays comparing known TS inhibitor 5-FU with compound 19-S in the indicated cell lines following a 72-hour drug incubation. Data are expressed as mean ± SD of 2 independent experiments; *n* = 4 to 5 technical replicates. (**D**) Immunoblot analysis showing TS overexpression following 5-FU treatment (TS^5FU^) and stable TS expression levels following compound 19-S treatment. PANC-1 cells were treated for 72 hours with the indicated 5-FU and 19-S concentrations. Experiment was repeated independently twice with similar results.

To determine the inhibition of TYMS activity, we utilized a tritium-based TYMS catalytic assay (Figure 1A) to quantify the conversion of dUMP, which is proportional to TYMS activity (33). The assay was based on a previously published protocol (33) and modified to determine the amount of purified human TYMS (hTYMS) to be used in the conversion of dUMP to dTMP. Increasing concentrations of purified hTYMS were used to determine hTYMS catalytic activity, and we established that 2 μg of hTYMS was an optimal dose to measure hTYMS catalytic activity (Supplemental Figure 2). Reactions were performed in the presence of compound 19-S or 5-FU metabolite controls, including FdUrd, which is known to inhibit TYMS activity, and 5-fluorouridine (FUrd), which does not inhibit TYMS activity (14). As expected, the 5-FU metabolite FdUrd reduced conversion of dUMP whereas FUrd was inactive (Figure 1B). Compound 19-S yielded comparable TYMS inhibition as seen with the 5-FU metabolite FdUrd (Figure 1B), verifying compound 19-S shows inhibition of TYMS activity.

To establish the cytotoxicity of compound 19-S, we used a panel of 4 established PDAC cell lines including 2 PDAC cell lines (PANC-1 and MIA PaCa-2) and 2 pancreatic neuroendocrine tumor (PanNET) cell lines (CM and BON). We chose these cell lines since patients with PDAC and PanNET respond to the TYMS inhibitors 5-FU (component in FOLFIRINOX treatment regimen) and capecitabine (component of CAPTEM treatment regimen), respectively. Treatment with compound 19-S resulted in potent cytotoxicity in 3 out of 4 cell lines examined (Figure 1C). The concentration for 50% of maximal inhibition of cell proliferation (GI_50_) in MIA PaCa-2 cells for compound 19-S was 16-fold lower than for 5-FU (1.26 μM versus 20.4 μM), and in CM cells the GI_50_ was over 150-fold lower than 5-FU (0.095 μM versus 14.8 μM). In PANC-1, the GI_50_ for compound 19-S was 1.9-fold lower than observed for 5-FU (8.35 μM versus 15.7 μM), and the difference in GI_50_ was further magnified by the broad hill slope observed in both dose-response curves (Figure 1C). For compound 19-S in BON cells, 50% viability was not reached, and GI_50_ could not be calculated; we did observe a more potent IC_50_ of 0.12 μM with 19-S compared with an IC_50_ of 7.1 μM for 5-FU (Figure 1C). The cytostatic effect of 19-S in BON cells may be due to the unique features of this type of carcinoid tumor, which retains differentiation and expresses functional neuropeptides, making it distinct from high-grade neuroendocrine PanNET tumors. In addition, we also tested the effect of 19-S compared with 5-FU in the H1048 small cell lung cancer cell line (SCLC) and observed that the GI_50_ for compound 19-S was 38-fold lower than for 5-FU (0.096 μM versus 3.63 μM) (Supplemental Figure 1B), verifying the broad cytotoxic effect of compound 19-S in different tumor subtypes.

Earlier studies have reported that a common molecular mechanism limiting the sustained therapeutic benefits of 5-FU therapy is drug-induced elevation of TYMS expression that contributes to increased drug resistance, limiting 5-FU effectiveness (19). Therefore, we tested if 19-S treatment also induced TYMS protein expression as compared with 5-FU treatment (Figure 1D). As expected, 5-FU induced 3-fold increase of TYMS expression with the characteristic band shift caused by the covalently bound 5-FU that retards the migration of TYMS. In contrast, there was no increase in steady-state TYMS levels after treatment with compound 19-S (Figure 1D), suggesting compound 19-S treatment can escape this drug-induced resistance mechanism.

In addition, we tested the capacity of PANC-1 and MIA PaCa-2 cells to acquire resistance to either 5-FU or compound 19-S. Parental cells were treated with a constant high concentration of 10 μM 19-S or 5-FU over 6 weeks and counted weekly. Both compounds 19-S and 5-FU showed a potent cytotoxic effect on PANC-1 and MIA PaCa-2 cells after 1 week (Supplemental Figure 3A). However, after 4 weeks of treatment with compound 19-S, there were no viable cells whereas 5-FU–treated cells remained detectible, and weekly cell counts of cells treated with 10 μM 5-FU showed relatively constant counts from weeks 3 to 7. This suggests that PANC-1 and MIA PaCa-2 cells acquired resistance to 5-FU but were killed by compound 19-S treatment (Supplemental Figure 3A). We also tested 5-FU and compound 19-S’s effect on the clonogenic capacity of PANC-1 cells that were engineered in our lab to develop resistance to 5-FU (Supplemental Methods). PANC-1 cells that were 5-FU resistant were treated with either 8.35 μM compound 19-S (concentration that corresponds to the GI_50_ for PANC-1 cells treated with compound 19-S, as shown in Figure 1D) or 6.5 μM 5-FU. The growth of 5-FU– and 19-S–treated cells was compared with the growth of untreated controls. We observed that 19-S treatment of 5-FU–resistant PANC-1 cells abolished colony formation while colonies were observed following 5-FU treatment and in untreated controls (Supplemental Figure 3B). These results suggest that 19-S treatment does not induce drug resistance in PANC-1 and MIA PaCa-2 cells and show that 19-S has a potent cytotoxic effect in cells that are resistant to 5-FU treatment. In addition, we compared 19-S efficacy between cancer and normal cells using MTT assay. We observed that 19-S inhibited growth of Colo230, and H630, while we did not detect inhibition of growth in normal fibroblast-like colon CCD18Co cells (Supplemental Figure 1C).

### Compound 19-S inhibits tumor growth in pancreatic xenograft tumor models.

Based on the in vitro studies demonstrating the ability of compound 19-S to inhibit TYMS catalytic activity (Figure 1B) and cause cytotoxicity in PDAC, PanNET, colon cancer, and SCLC cell lines (Figure 1C and Supplemental Figure 1, B and C), we studied the in vivo effects of compound 19-S in 2 xenograft tumor models available in our laboratory. We first tested a subcutaneous tumor model with the PANC-1 cell line expressing firefly luciferase (Luc-PANC-1) and then tested a disseminated tumor model with the CM cell line also expressing firefly luciferase (Luc-CM).

**Figure 2:**
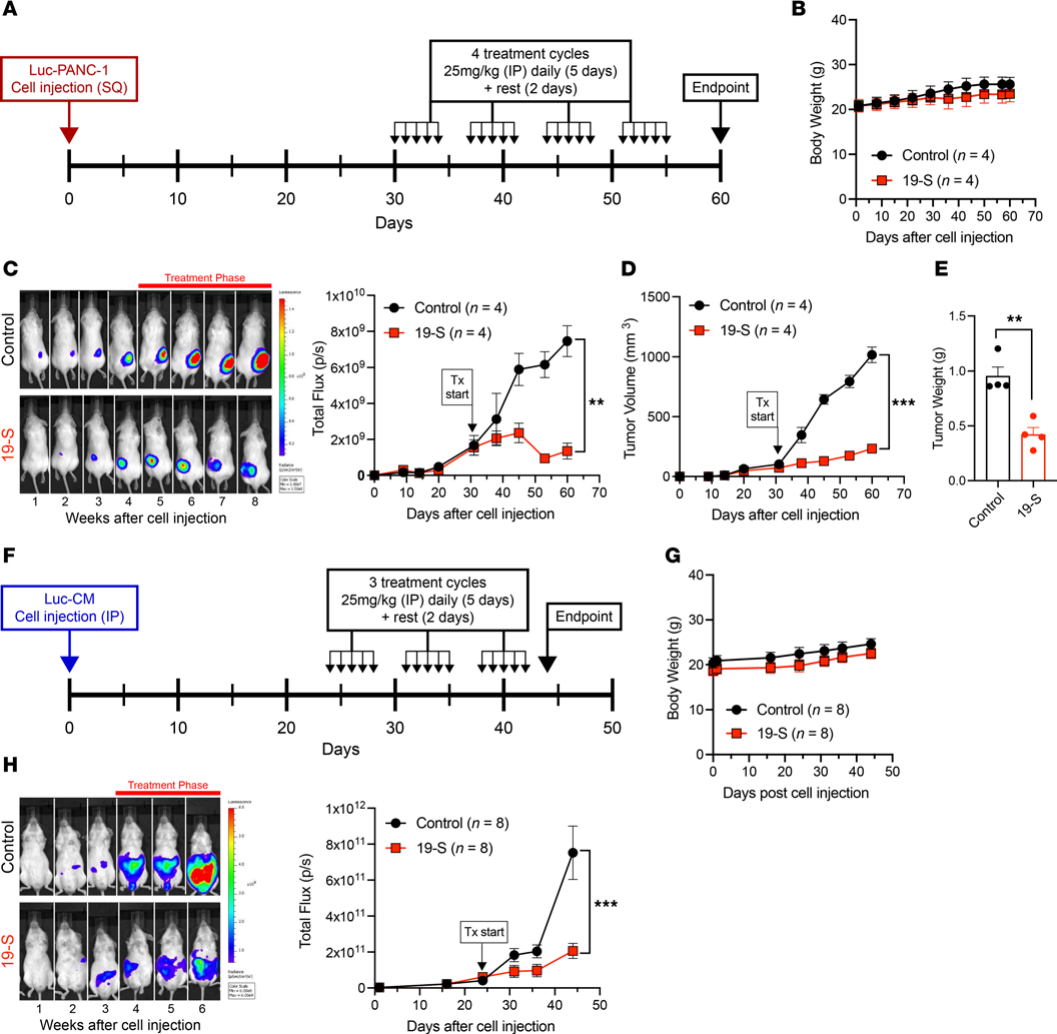
Compound 19-S inhibits tumor growth and progression. (**A**) Experimental timeline for the subcutaneous (SQ) Luc-PANC-1 cell line–derived tumor model treated with compound 19-S or vehicle control. Compound 19-S (25 mg/kg) or vehicle control treatment cycles were administered by IP injection. For each cycle animals were treated once a day for 5 continuous days, then allowed 2 days’ rest when no treatments were administered. Tumor-bearing mice received a total of 4 treatment cycles before endpoint (*n* = 4 per cohort). (**B**) Effect of compound 19-S and vehicle control administered by IP injection on body weight as described in **A**. (**C**) Bioluminescence imaging of Luc-PANC-1–derived tumors and quantification of bioluminescence photon flux over time for animals treated with compound 19-S or vehicle control (**P = 0.0023). (**D**) Luc-PANC-1 tumor volumes for animals treated with compound 19-S or vehicle control (***P < 0.0001). (**E**) Final excised Luc-PANC-1 tumor weight for animals treated with compound 19-S or vehicle control (**P = 0.0029). (**F**) Timeline indicating the IP injection of Luc-CM cells to generate a disseminated tumor model and compound 19-S (25 mg/kg) or vehicle control treatment cycles administered by IP injection. For each cycle animals were treated once a day for 5 continuous days, then allowed 2 days’ rest when no treatments were administered. Tumor-bearing animals received 3 treatment cycles before endpoint (*n* = 8 per cohort). (**G**) Effect of compound 19-S and vehicle control administered by IP injection on body weight for Luc-CM tumor–bearing NSG mice. (**H**) Bioluminescence imaging of Luc-CM tumor–bearing NSG mice and quantification of bioluminescence photon flux from the abdominal region of animals treated with compound 19-S (*n* = 8) or vehicle control (*n* = 8). Data are expressed as mean ± SEM, ***P < 0.0001. Statistical analysis in **C**, **D**, and **H** was performed using 2-way ANOVA; in **E**, unpaired 2-tailed Student’s *t* test with Welch’s correction was used.

In the subcutaneous tumor model, the resulting Luc-PANC-1 xenograft tumors were monitored by direct tumor measurements and bioluminescence photon flux that is proportional to tumor size. The experimental timeline (Figure 2A) illustrates the treatment cycles used for compound 19-S and the vehicle control delivered by IP injections to tumor-bearing NSG mice. Treatment was initiated 30 days after Luc-PANC-1 cell line subcutaneous injections (5 × 10^6^ cells), when tumors reached 100 mm^3^ in volume, generating a bioluminescence photon flux of 3 × 10^9^ to 4 × 10^9^ photons/s. Animals were randomly assigned into treatment groups receiving a total of 4 treatment cycles, with each cycle defined as either 25 mg/kg 19-S treated once a day for 5 continuous days, then allowed 2 days’ rest without treatment, or an equivalent volume of the vehicle control delivered by IP injection. Toxicity studies demonstrated that the 25 mg/kg treatment dose was the maximum tolerated dosage (MTD) (Supplemental Figure 4) since no changes in body weight (Supplemental Figure 4B) and no evidence of drug tissue injury were detected in any of the organs examined both grossly and in histological sections following treatment with 25 mg/kg 19-S compared to control mice (Supplemental Figure 4C). Body weight was monitored for all animals throughout the experiment, showing no significant deviations between 19-S– or control vehicle–treated animals at the 25 mg/kg treatment dose (Figure 2B), and no adverse animal behavior was observed for both treatment groups. While vehicle control–treated tumors rapidly progressed, treatment with 19-S significantly inhibited tumor progression (*P* = 0.0023, Figure 2C and Supplemental Figure 5) and tumor volume (*P* < 0.0001, Figure 2D). After completion of the fourth treatment cycle, animals were given a 5-day rest without treatment, and then all animals were euthanized because of the tumors reaching experimental endpoints in the control animals. Tumors from both treatment groups were excised, and the tumor mass was weighed (Figure 2E). Consistent with the bioluminescence photon flux and tumor volume, the final tumor mass for the compound 19-S treatment group was significantly reduced compared with the tumors from the vehicle control group (*P* = 0.0029). These data from the subcutaneous xenograft pancreatic tumor model demonstrate the ability of compound 19-S to inhibit tumor progression of localized solid tumors. In addition, we compared the antitumor effect of compound 19-S to 5-FU in the PANC-1 xenograft model following the same procedure described above. We also established the MTD for 5-FU treatment in NSG and FVB/129/Sv mice to be 25 mg/kg (Supplemental Figure 6, A and B). We observed that both 5-FU– and 19-S–treated mice showed a significant reduction in tumor volume compared with control mice (*P* < 0.0001); however, mice treated with compound 19-S had further reduced tumor volume when compared with the volume after 5-FU treatment (*P* = 0.049) (Supplemental Figure 6, C and D). In addition, we compared TYMS levels in vivo following Luc-PANC-1 xenograft treatment with 5-FU and compound 19-S at 25 mg/kg daily IP injection for 5 consecutive days (Supplemental Figure 6E). As expected, TYMS expression levels in 5-FU–treated tumors were higher than TYMS protein level in vehicle-treated controls (*P* = 0.003) (Supplemental Figure 6F). Most importantly, TYMS level did not increase in vivo after compound 19-S treatment (*P* = 0.0005).

To further test the effect of compound 19-S on tumor growth and progression, we utilized a Luc-CM disseminated xenograft tumor model. Luc-CM cancer cells were delivered by IP injection, allowing distribution throughout the abdominal cavity. Tumor progression was then monitored by the bioluminescence photon flux from the abdominal region, and treatment was initiated after 24 days, when the abdominal region bioluminescence photon flux was in the range of 5 × 10^10^ photons/s. Animals were randomly assigned into treatment groups receiving either 25 mg/kg 19-S or an equivalent volume of the vehicle control delivered by IP injection. A total of 3 treatment cycles were administered, and for each treatment cycle animals were treated once a day for 5 continuous days and then allowed 2 days’ rest without treatment (Figure 2F). Similar to the data obtained with the subcutaneous tumor model, the animals with Luc-CM–derived disseminated tumors did not show significant deviations in body weight or display adverse behavior because of 19-S treatment (Figure 2G). Although this disseminated tumor model does not allow direct tumor measurements, the bioluminescence photon flux from the abdominal region is proportional to the tumor burden. Once treatment was initiated, the vehicle control group experienced rapid tumor progression indicated by the increasing bioluminescence photon flux from the abdominal region, while 19-S treatment efficiently inhibited tumor progression (*P* < 0.0001, Figure 2H and Supplemental Figure 7). In addition, this disseminated tumor model with Luc-CM cells was also used to determine if oral delivery of 19-S was well tolerated and resulted in similar inhibition of tumor progression. When treated with the same dose of 19-S (25 mg/kg) by oral gavage, no significant deviations in body weight were observed, and a similar inhibition of tumor progression as compared to delivery by IP injection (Supplemental Figure 8) was observed. Collectively, both xenograft tumor models demonstrate compound 19-S is tolerated without apparent toxicity while inhibiting tumor growth and its progression.

### Compound 19-S prolongs survival in a pancreatic xenograft tumor model.

**Figure 3:**
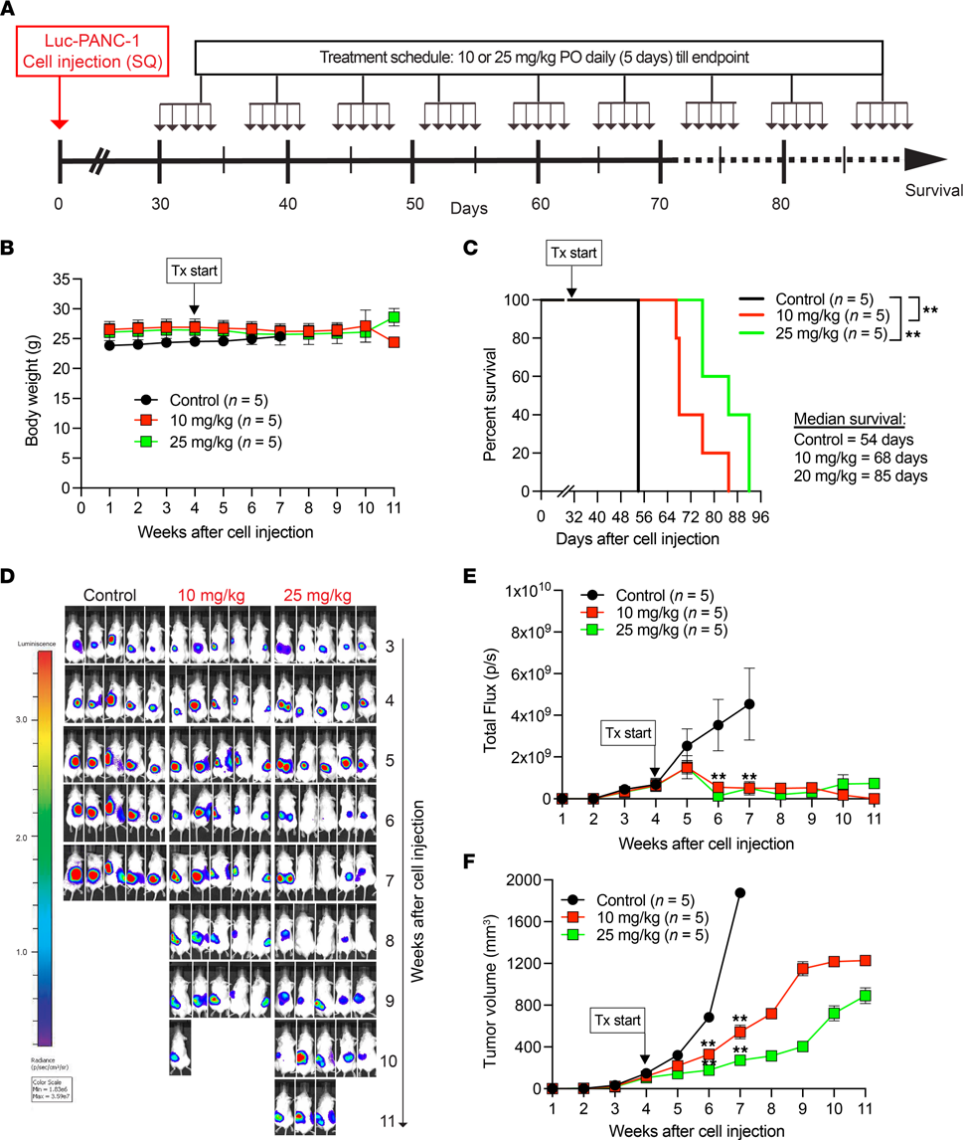
Compound 19-S prolongs survival in a pancreatic xenograft tumor model. (**A**) Experimental timeline indicating the subcutaneous Luc-PANC-1 cell line–derived tumor model treated with compound 19-S or vehicle control. Timeline indicates the subcutaneous injection of Luc-PANC-1 cells in NSG mice to generate tumors and compound 19-S (10 mg/kg or 25 mg/kg) or vehicle control treatment cycles administered by oral gavage (per os; PO). For each cycle animals were treated once a day for 5 continuous days, then allowed 2 days’ rest when no treatments were administered. Animals received treatment until survival endpoint. (**B**) Effect of compound 19-S and vehicle control administered PO on body weight for Luc-PANC-1 tumor–bearing NSG mice. Data are presented as mean body weight of 5 animals per group ± SEM. (**C**) Kaplan-Meier survival analysis for Luc-PANC-1–injected NSG mice treated with compound 19-S (10 mg/kg or 25 mg/kg, *n* = 5 per group) or vehicle control (*n* = 5); **P = 0.003. Log-rank (Mantel-Cox) test was used to calculate *P* values. (**D** and **E**) Bioluminescence imaging of Luc-PANC-1–derived tumors and quantification of bioluminescence photon flux over time for animals treated with compound 19-S (10 mg/kg or 25 mg/kg) or vehicle control. (**F**) Luc-PANC-1 tumor volumes for animals treated with compound 19-S (10 mg/kg or 25 mg/kg) or vehicle control. In **E** and **F**, data are presented as mean total flux or tumor volume, respectively, of 5 animals per group ± SEM; **P ≤ 0.01 by 2-tailed Mann-Whitney *t* test.

Since we observed that compound 19-S inhibited tumor growth in 2 xenograft tumor models (Figure 2 and Supplemental Figure 8), we then asked whether the antitumor effect of compound 19-S would prolong survival. We tested the Luc-PANC-1 subcutaneous tumor model with the PANC-1 cell line expressing firefly luciferase and followed the experimental timeline shown in Figure 3A. Treatment was initiated 30 days after 5 × 10^6^ Luc-PANC-1 cell injections, when tumors reached 100 mm^3^. Animals were randomly assigned into treatment groups receiving 10 or 25 mg/kg compound 19-S PO once daily for 5 consecutive days and then allowed 2 days to rest without treatment; control animals received vehicle (corn oil) on the same schedule. Treatment was administered until survival endpoint as determined by the IACUC-approved protocol. We chose PO over IP treatment since we observed similar antitumor effect by either route (Figure 2 and Supplemental Figure 8). Body weight was monitored throughout the experiment and showed no significant differences between 19-S groups and vehicle control animals (Figure 3B). Treatment with 19-S at either dose resulted in a statistically significant (*P* = 0.003) prolonged survival compared with vehicle control (Figure 3C). While controls had to be sacrificed at 7 weeks due to tumor size, 19-S–treated animals reached endpoint between 4 and 5 weeks after controls because of tumor ulceration rather than tumor growth. Thus, median survival increased from 54 days in control-treated animals to 68 and 85 days in 10 mg/kg or 25 mg/kg 19-S–treated animals, respectively. Compound 19-S treatment at both 10 mg/kg and 25 mg/kg concentration inhibited tumor progression as determined by the reduced abdominal bioluminescence photon flux. For example, following 2 weeks of treatment with 19-S, there was a significant reduction of total flux compared with vehicle control measured at the 6-week period after initial tumor cell injection (*P* = 0.0079, Figure 3, D and E), consistent with a significant inhibition of tumor volume (*P* = 0.0079) that was maintained until endpoint (Figure 3F). These data demonstrate the effectiveness of daily oral 19-S to prolong survival and control disease progression.

### Compound 19-S prolongs survival in a GEMM.

**Figure 4:**
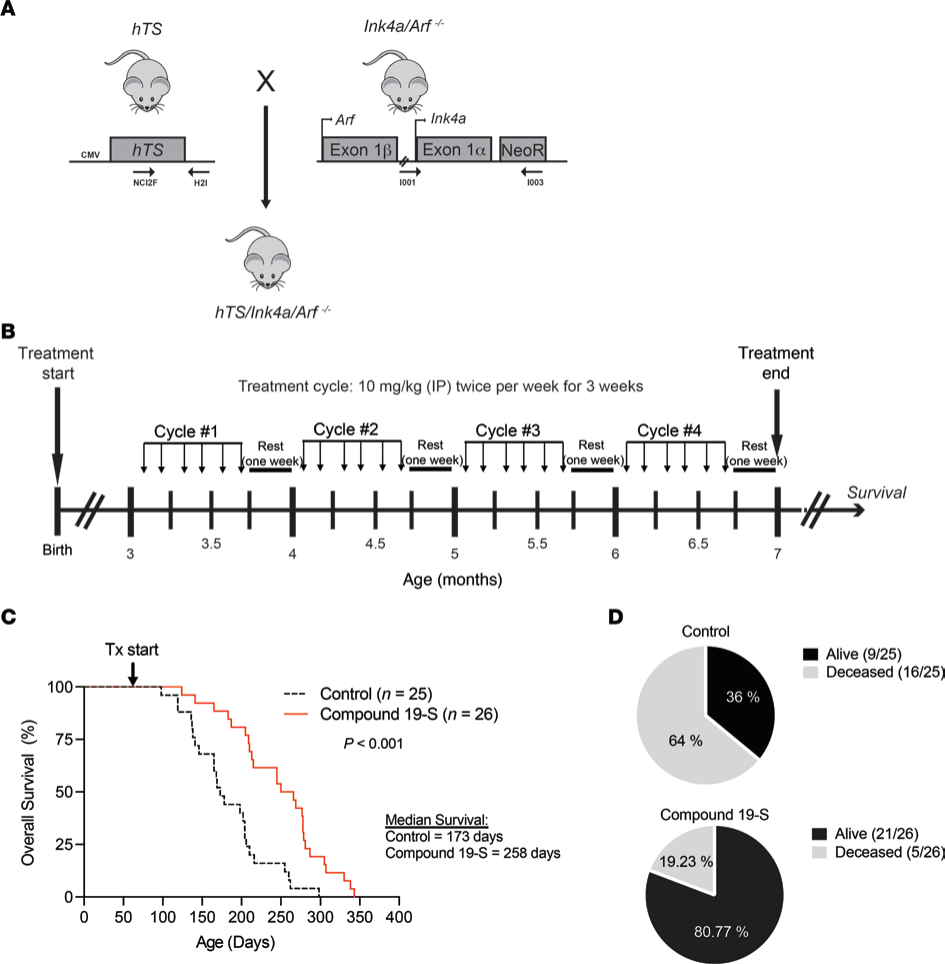
Compound 19-S prolongs survival in an *hTS/Ink4a/Arf ^–/–^* GEMM. (**A**) Generation of *hTS/Ink4a/Arf^–/–^* mice by crossing hTS transgenic mice with *Ink4a/Arf^–/–^* mice. Locations of forward and reverse primers for the detection of hTS transgene and Ink4a/Arf locus are shown by arrows. (**B**) Experimental timeline for *hTS/Ink4a/Arf^–/–^* GEMM survival experiments. Treatment started when animals were 3 months of age, and a total of 4 treatment cycles were administered. For each treatment cycle, animals were administered compound 19-S (10 mg/kg) or vehicle control by IP injection twice weekly for 3 weeks and then allowed 1-week rest with no treatment. Animals were then monitored until survival endpoint. (**C**) Kaplan-Meier survival analysis for *hTS/Ink4a/Arf ^–/–^* animals treated with compound 19-S (*n* = 26) or vehicle control (*n* = 25). ***P < 0.0001 was calculated by log-rank (Mantel-Cox) test. Tx, treatment. (**D**) Diagrams indicating the surviving and deceased fractions for the compound 19-S treatment group and vehicle control group at the end of treatment cycle 4, when treatment was discontinued for all animals.

We also tested whether compound 19-S blocked tumor progression to increase survival using an *hTS/Ink4a/Arf^–/–^* GEMM. This transgenic model developed in our laboratory was generated by crossing mice that express hTS (34) with *Ink4a/Arf*–null mice (35) to generate *hTS/Ink4a/Arf^–/–^* mice on a mixed FVB/129/Sv background (Figure 4A) (36). To test the potency of 19-S in prolonging survival of the *hTS/Ink4a/Arf^–/–^* GEMM, we first measured MTD in wild-type FVB/129/Sv and in *Ink4a/Arf^–/–^* FVB/129/Sv mice (Supplemental Figure 9). We demonstrated that daily IP delivery of 25 mg/kg 19-S for 3 weeks was the highest drug dose delivered when all animals were alive following 1 month after treatment. For survival studies, 3-month-old *hTS/Ink4a/Arf^–/–^* animals were randomized into 2 treatment cohorts receiving either 19-S (*n* = 26) or vehicle control (*n* = 25). For each treatment cycle, animals were administered 10 mg/kg of 19-S or vehicle control by IP injection twice a week for 3 weeks, then allowed a 1-week rest (Figure 4B). Animals received 4 treatment cycles and were then monitored once off treatment until survival endpoint. We found that twice-weekly treatment with 10 mg/kg of 19-S resulted in a statistically significantly prolonged survival (258 days) as compared with vehicle control mice (173 days) (*P* < 0.0001) (Figure 4C). In addition, the percentage of animals surviving after completing treatment with 19-S in the final cycle 4 was 80.7% (21/26 animals) while it was 36% (9/25 animals) in the control group (Figure 4D). Histological sections of kidney, liver, pancreas, lung, spleen, and brain of *hTS/Ink4a/Arf^–/–^* 19-S–treated mice were evaluated by masked pathologist, verifying that no evidence of drug injury was found in any of the organs examined compared to vehicle control–treated mice (Supplemental Figure 10). These data demonstrate the effectiveness of compound 19-S to prolong survival and control disease progression in the *hTS/Ink4a/Arf^–/–^* GEMM after only 4 cycles of 19-S treatment.

### Synthesis of compound 19-S analogs.

**Figure 5:**
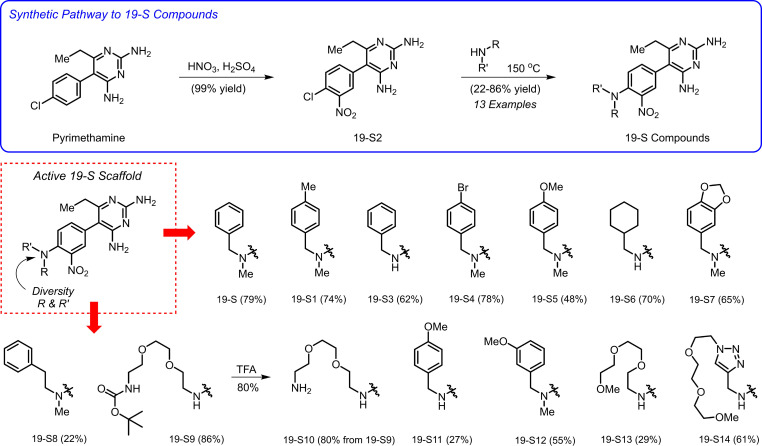
Synthesis of compound 19-S analogs. Reagents and conditions: synthesis of 19-S2: pyrimethamine, HNO_3_, H_2_SO_4_, 0°C to 50°C, 80 minutes, 99% yield. Synthesis of 19-S, 19-S1, 19-S3 through 19-S9, and 19-S11 through 19-S14: 19-S2, amine (neat), 150°C, sealed tube, 6 hours, 22–86% yield. Synthesis of 19-S10: 19-S9, TFA, CH_2_Cl_2_, room temperature, 80% yield. TFA, trifluoroacetic acid.

After verifying the safety and tumor-inhibitory activity of 19-S, a series of 19-S analogs were synthesized (Figure 5). These analogs introduced structural diversity with a focus designed to increase aqueous solubility. Generating this diverse series of 19-S analogs provides the ability to determine structural features with increased potency, while allowing a better understanding of which structural features contribute to the observed biological activities.

Compound 19-S and related analogs were synthesized from pyrimethamine using a 2-step route that involved i) nitration of the *p*-chlorobenzene ring of pyrimethamine and ii) subsequent nucleophilic aromatic substitution with several primary or secondary amines (Figure 5). This short synthetic sequence was used to access 13 analogs of 19-S in 22%–86% yield. In addition, 19-S10 was prepared using TFA for removal of the Boc group of 19-S9. This robust synthetic route enabled rapid access to sufficient material (300 mg to 1 g of several analogs) for both in vitro and in vivo studies. See Supplemental Methods section titled “Chemical Synthesis, Characterization Data, and NMR Spectra” for details.

The precursor molecule pyrimethamine, used in the synthesis of the 19-S and its analogs, is a known inhibitor of the protozoan DHFR enzyme (37). Therefore, we also explored a possible dual and/or complementary mechanism of action for this class of 19-S inhibitors given that TYMS and DHFR are both folate-dependent enzymes.

### Compound 19-S and its analogs 19-S5 and 19-S7 show dual TYMS and DHFR inhibition.

**Figure 6:**
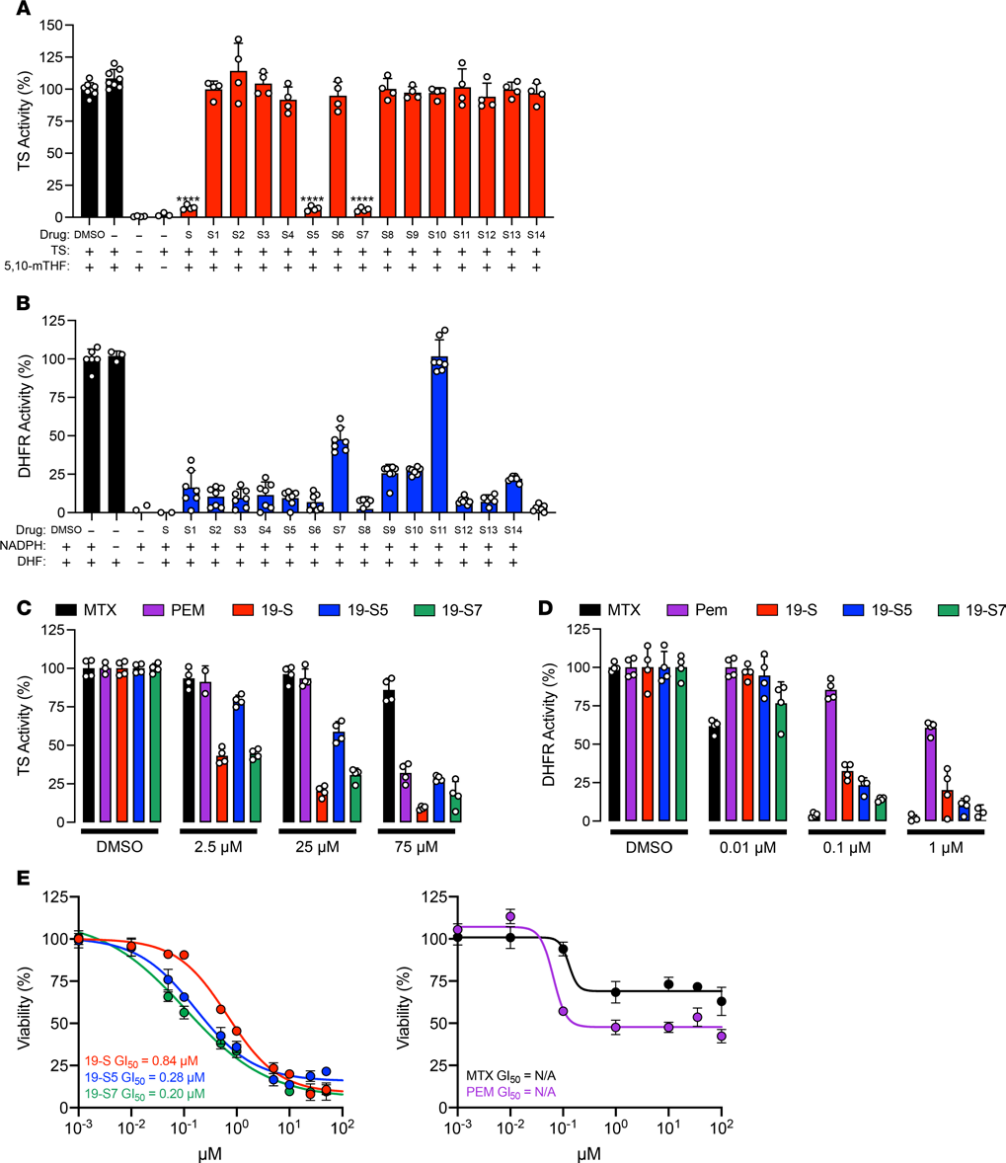
Select compound 19-S series analogs show dual TS and DHFR inhibition. (**A**) Tritium-based TS activity assay screen of compound 19-S series analogs. Compounds were initially screened at 250 μM to determine analogs showing TS inhibition. Mean ± SD of 4 data points from 2 independent experiments shown. DMSO, TS + 5,10-mTHF, and 5,10-mTHF controls represent 8 data points. *P* values are calculated using 2-tailed unpaired *t* test, ****P ≤ 0.0001. (**B**) Absorbance-based DHFR activity assay screen of compound 19-S series analogs; all compounds were initially screened at 1 μM concentration. Data are expressed as mean ± SD of *n* = 7 from 2 independent experiments. (**C**) Comparison showing TS activity utilizing the tritium-based activity assay in the presence of pemetrexed (PEM), methotrexate (MTX), and compounds 19-S, 19-S5, and 19-S7 at the indicated concentrations. Data are expressed as mean ± SD of *n* = 4 from 2 independent experiments. (**D**) Comparison showing DHFR activity utilizing the absorbance-based activity assay in the presence of PEM, MTX, and compounds 19-S, 19-S5, and 19-S7 at the indicated concentrations. Data are expressed as mean ± SD of *n* = 4. (**E**) MIA PaCa-2 cell line viability assays following 72-hour drug treatment with compound 19-S and 19-S series analogs 19-S5 and 19-S7 and known control antifolate inhibitors PEM and MTX. Data are expressed as mean ± SD (triplicates).

This potentially new, structurally diverse 19-S series compound library with 15 compounds including the lead compound 19-S and its 14 analogs, 19-S1 through 19-S14, were then screened to establish the ability of each compound to inhibit TYMS catalytic activity (Figure 6A). In addition to lead compound 19-S, 19-S5 and 19-S7 inhibited TYMS activity, while the other analogs did not. The initial drug screen with the tritium-based TYMS activity assay utilized a higher threshold for the drug concentration (250 μM), which was designed to identify compounds showing even weak TYMS inhibition. We observed that compounds showed either near-complete inhibition of TYMS activity (*P* < 0.0001) or no inhibition at all (Figure 6A). This distinct binary difference suggests there could be a stringent binding criterion for TYMS inhibition dependent upon the benzylmethylamine moiety.

There are 2 major classes of clinically approved TYMS inhibitors, fluoropyrimidines and antifolates, and both mimic the normal substrates involved in the TYMS-catalyzed conversion of dUMP to dTMP (1). Therefore, if the 19-S series compounds also inhibit DHFR activity, this would suggest the folate site as the target for inhibition by these compounds. The 15-compound library of the 19-S series was used to screen for DHFR inhibition using the standard absorbance-based activity assay as described in Methods (Figure 6B). With the exception of 19-S10, all compounds in the 19-S series library were able to inhibit DHFR activity at 1 μM concentration (Figure 6B).

To further study and discriminate the inhibitory activity for the dual TYMS/DHFR inhibitors 19-S, 19-S5, and 19-S7, we repeated the tritium-based TYMS catalytic activity and DHFR absorbance-based activity assays at decreasing drug concentrations. Additionally, the classical antifolate compounds pemetrexed (PEM) and methotrexate (MTX) were included as controls to provide a reference for comparison given the different assays utilized to determine TYMS and DHFR activity. As expected, PEM displayed greater TYMS inhibition than the MTX antifolate while MTX was a more potent inhibitor of DHFR (Figure 6, C and D). Compounds 19-S, 19-S5, and 19-S7 demonstrated greater TYMS inhibition than both MTX and PEM (Figure 6C). When DHFR activity was determined using reduced concentrations of each inhibitor, as expected, MTX retained the expected potent inhibition while PEM was less effective against DHFR (Figure 6D). In contrast, compounds 19-S, 19-S5, and 19-S7 showed both potent TYMS and DHFR inhibition (Figure 6, C and D).

While compounds 19-S, 19-S5, and 19-S7 demonstrated potent dual TYMS- and DHFR-inhibitory activity, these compounds are structurally distinct from classical antifolates such as PEM and MTX. Due to the structural similarity of classical antifolates as analogs of normal folate substrates, their cytotoxicity is dependent on folate transport and metabolic pathways. These include factors such as energy-dependent membrane transport into cells by the reduced folate carrier and the intracellular conversion to its polyglutamated metabolite by FPGS (25, 26, 38, 39). Therefore, it is important to validate the purified enzyme assay results using cell-based assays that are dependent on factors such as cellular uptake and transport as well as drug metabolism. Therefore, cell viability assays were performed with compounds 19-S, 19-S5, 19-S7, as well as the classical antifolates PEM and MTX, using MIA PaCa-2, a PDAC cell line more sensitive to 19-S than PANC-1 cells as shown in Figure 1C. Following the 72-hour treatment, 19-S, 19-S5, and 19-S7 demonstrated potent cytotoxicity with GI_50_ values ranging from 0.20 μM to 0.84 μM, while the classical antifolates showed only a cytostatic effect and were unable to reduce viability below 50% at the highest 350 μM concentration (Figure 6E). These data further demonstrate that this new class of dual TYMS/DHFR inhibitors exemplified by compounds 19-S, 19-S5, and 19-S7 is structurally distinct from classical folate antimetabolites.

### Mechanism of TYMS inhibition reveals nonclassical antifolate inhibitors.

**Figure 7:**
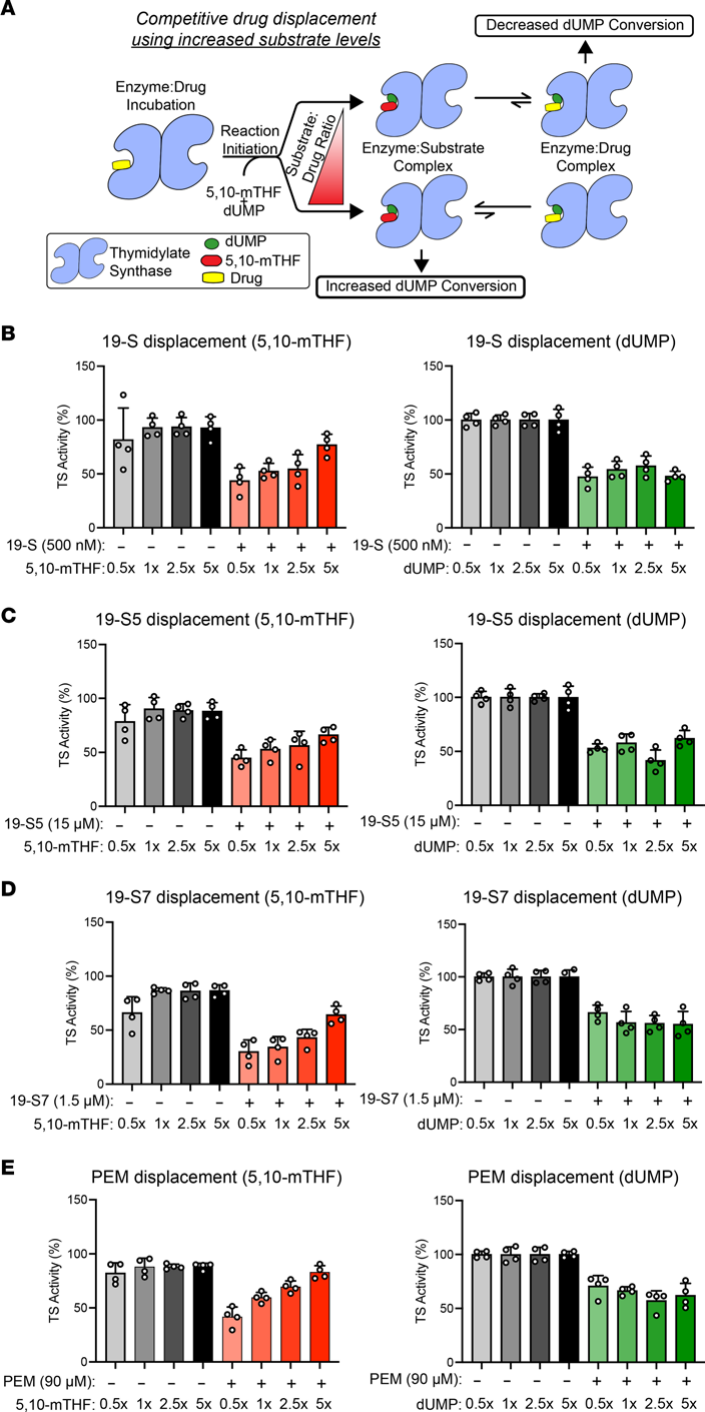
Mechanism of TS inhibition reveals nonclassical antifolate inhibitors. (**A**) Illustration outlining the drug displacement from TS with increasing substrate concentrations to highlight how increased molar ratios of the competing substrate will displace the prebound drug and shift the equilibrium to the enzyme/substrate complex. resulting in an increased conversion of tritiated dUMP. (**B**–**E**) Tritium assay for TS activity with increasing 5,10-mTHF or dUMP ratios for 19-S (19-S) (**B**), 19-S5 (19-S5) (**C**), 19-S7 (19-S7) (**D**), and control classical antifolate PEM (**E**). TS was preincubated with each compound using the indicated concentration; for each compound the concentration required for a 50% reduction in dUMP conversion after a 30-minute reaction was utilized to allow changes in dUMP conversion to be observed. Increasing TS activity with increasing 5,10-mTHF ratios was observed for all compounds, including the control antifolate PEM, while increasing dUMP ratios had no significant effect on TS activity, indicating compounds 19-S, 19-S5, and 19-S7 act as nonclassical antifolate inhibitors by competing only with the 5,10-mTHF substrate. Data are expressed as mean ± SD of *n* = 4 from 2 independent experiments.

To further study the nonclassical antifolate mechanism of dual TYMS/DHFR inhibition observed with 19-S, 19-S5, and 19-S7, we designed a series of drug displacement experiments based on the established tritium TYMS activity assay. For these experiments the TYMS protein was first incubated with each drug, and the reaction was then initiated by addition of 5,10-mTHF and dUMP using different concentrations of either 5,10-mTHF or dUMP to change the substrate/drug ratio. If there was competition between one of the substrates and the drug for the same binding site, then increasing the substrate concentration would displace more of the prebound drug and shift the equilibrium toward the enzyme/substrate complex, thus allowing the conversion of dUMP to proceed (Figure 7A).

First, we verified that increasing the concentration of either the dUMP substrate or the 5,10-mTHF cofactor did not affect the amount of dUMP converted during the reaction and therefore did not increase the scintillation counts (Supplemental Figure 11). The different substrate concentrations are expressed relative to the standard concentrations used for the tritium assay. These standard saturating substrate concentrations were previously determined when the assay was optimized to ensure that either substrate was not depleted during the reaction. Since TYMS activity is quantified by conversion of the tritiated dUMP tracer ([5-^3^H]dUMP), maintaining the [5-^3^H]dUMP/dUMP ratio is essential for comparing the reactions performed with increased concentrations of dUMP. For example, increasing dUMP concentration will cause a reduction in [5-^3^H]dUMP/dUMP ratio and could be perceived as a reduction in TYMS activity. As observed from the raw scintillation count data, increasing the 5,10-mTHF concentration by 2.5-fold or 5-fold did not have an impact on the conversion of dUMP (Supplemental Figure 11). As expected, when the [5-^3^H]dUMP/dUMP ratio was maintained and the total dUMP concentration was increased, there was no impact on the total amount of [5-^3^H]dUMP converted during the reaction (Supplemental Figure 11). These data validate the amount of dUMP conversion during the reaction does not increase with increasing 5,10-mTHF or dUMP concentrations.

We then performed the drug displacement assay for 19-S, 19-S5, and 19-S7. We found that increasing 5,10-mTHF concentration increased the amount of dUMP conversion during the reaction for all 3 of the 19-S series compounds (Figure 7, B–D). This demonstrates that 5,10-mTHF can displace the prebound 19-S series compounds, shifting the equilibrium toward the enzyme/substrate complex, allowing increased conversion of dUMP. When the reactions with each of the 19-S series compounds were performed with increasing dUMP concentrations, the amount of dUMP conversion did not increase (Figure 7, B–D), demonstrating dUMP is not competing for the same binding site as the 19-S series compounds. Since PEM is a classical antifolate known to bind in the TYMS folate site, we performed the drug displacement assay with PEM as an additional control. As expected, increasing the 5,10-mTHF concentration increased the dUMP conversion because of displacement of PEM from the folate site, and increasing dUMP concentration did not increase dUMP conversion (Figure 7E). In summary, these data provide evidence that the mechanism of TYMS inhibition for compounds 19-S, 19-S5, and 19-S7 is through blocking the folate-binding site and therefore acting as “nonclassical” antifolates.

### Structural basis for dual TYMS/DHFR inhibition.

**Figure 8:**
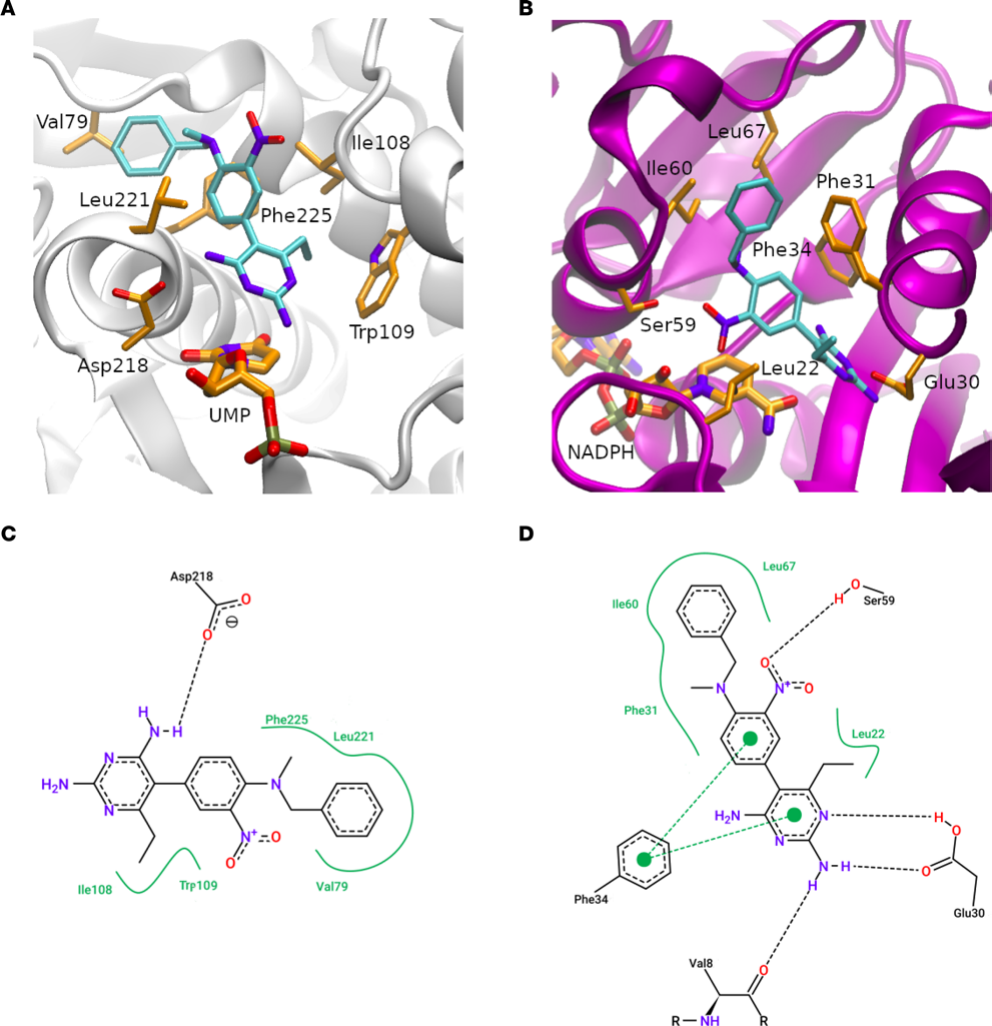
Proposed binding mode for compound 19-S. (**A**–**D**) Compound 19-S at canonical binding sites of TS and DHFR, respectively. **A** and **B** show the protein backbone as gray and purple ribbons for TS and DHFR, respectively. Cofactors, key residues, and compound 19-S are depicted as sticks and colored by element with carbons, oxygens, nitrogen atoms, and phosphorous in orange, red, blue, and brown, respectively (except compound 19-S with carbons in cyan). **C** and **D** show a 2D diagram of the protein-ligand interactions between compound 19-S and TS and DHFR, respectively. Key residues and 19-S are colored by elements with carbons, oxygens, and nitrogen atoms in black, red, and blue, respectively. Polar and aromatic interactions are presented as black and green dotted dashed lines, respectively. Nonpolar interactions are presented as a continuous green line surrounding the ligand functional groups.

Analysis of the molecular docking simulation suggests that 19-S binds the active site of TYMS occupying the folate-binding pocket (Figure 8, A and C). This proposes a binding model in which the aminopyrimidine group establishes the main interactions, involving a hydrogen bond with Asp218. The benzyl group lies on an adjacent hydrophobic region, establishing several nonpolar interactions. The nitro group is exposed to the solvent and apparently does not interact with any residue.

On the other hand, 19-S also binds the active site of DHFR occupying the folate-binding pocket (Figure 8, B and D). The proposed ligand-binding model agrees with previously reported structures (Protein Data Bank ID: 4KAK, Supplemental Figure 12, and Supplemental Table 1) in which the aminopyrimidine group also establishes a main interaction with the protein. The model suggests that compound 19-S established more polar and aromatic interactions with DHFR than TYMS. The nitro group is involved in a hydrogen bond with Ser59, and the aminopyrimidine group could interact with the Glu30 side chain, Val8 backbone, and Phe34 ring. Finally, as observed in TYMS, the benzyl group is placed on an adjacent hydrophobic region, establishing several nonpolar interactions. To validate the binding of 19-S to predicted amino acids on TYMS and DHFR, we performed in silico alanine mutational analysis (IAS), which is a standard computational method to estimate the contribution of the individual amino acids toward the binding of a given ligand (40). During the IAS simulation, each residue of the ligand-binding site is mutated to alanine, and the binding free energy is performed by IAS using pyFoldX software (41) (Supplemental Methods). We observed a positive change in free energy when substituting residues in TYMS and DHFR that bind 19-S to alanine, validating the predicted amino acid contribution to the interaction with 19-S (Supplemental Tables 2 and 3).

### Compounds 19-S and 19-S7 prevent tumor progression following oral delivery.

**Figure 9:**
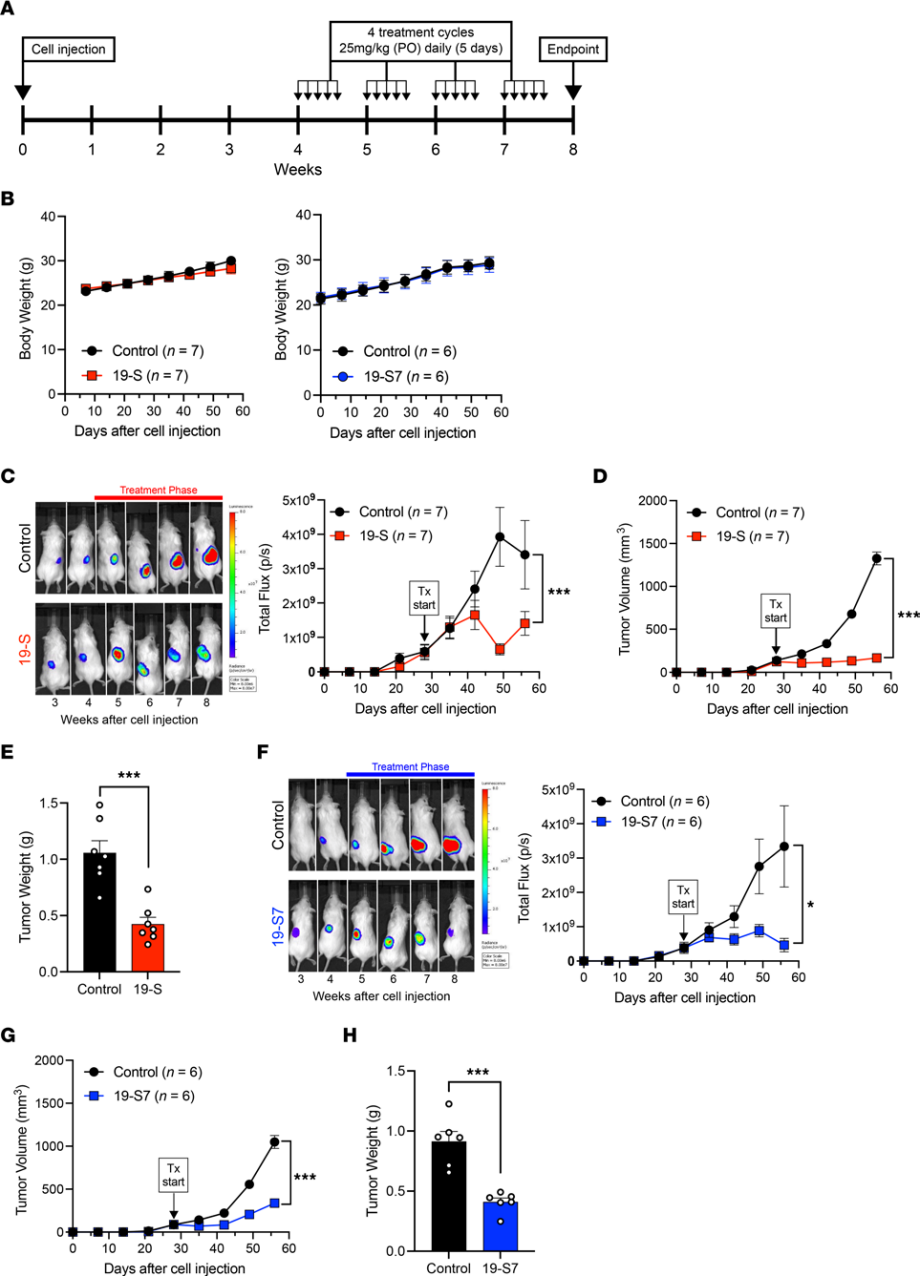
Compounds 19-S and 19-S7 prevent tumor progression without signs of toxicity following oral delivery. (**A**) Timeline indicating the subcutaneous injection of Luc-PANC-1 cells to generate subcutaneous tumors and the treatment cycles for compound 19-S (25 mg/kg), compound 19-S7 (25 mg/kg), or vehicle control administered PO. For each cycle animals were treated once a day for 5 continuous days, then allowed 2 days’ rest when no treatments were administered. NSG mice received 4 treatment cycles until endpoint. (**B**) Effect of compound 19-S and 19-S7 treatment with matched vehicle control treatment on body weight for Luc-PANC-1 tumor–bearing NSG mice. Data are expressed as mean ± SEM of compound 19-S (*n* = 7), 19-S7 (*n* = 6), and controls (*n* = 7 and *n* = 6, respectively). (**C**) Bioluminescence imaging of Luc-PANC-1–derived tumors and quantification of bioluminescence photon flux over time for animals treated PO with compound 19-S or vehicle control (***P = 0.0002). (**D**) Luc-PANC-1 tumor volumes for animals treated with compound 19-S or vehicle control administered PO (***P < 0.0001). (**E**) Final excised Luc-PANC-1 tumor weight for animals treated PO with compound 19-S or vehicle control (***P = 0.0010). (**F**) Bioluminescence imaging of Luc-PANC-1 derived tumors and quantification of bioluminescence photon flux over time for animals treated PO with compound 19-S7 or vehicle control (*P = 0.0192). (**G**) Luc-PANC-1 tumor volumes for animals treated PO with compound 19-S7 or vehicle control (***P < 0.0001). (**H**) Final excised Luc-PANC-1 tumor weight for animals treated PO with compound 19-S7 or vehicle control, ***P = 0.0010. Data are expressed as mean ± SEM of *n* = 7 for 19-S and *n* = 6 for 19-S7 treatment. Statistical analysis in **C**, **D**, **F**, and **G** was performed using 2-way ANOVA; in **E** and **H** unpaired *t* test with Welch’s correction was used.

Classical antifolates such as PEM have shown improvements in disease survival when continued in the maintenance phase of cancer treatment (42). Due to the poor bioavailability of classical antifolates, drug treatment often requires intravenous infusions at MTD every 21 days (42, 43). However, oral administration of chemotherapy treatment is preferred in clinical management, especially for maintenance therapy, allowing for more continuous, metronomic treatment protocols. Therefore, we tested the toxicity and antitumor activity following oral administration of compound 19-S and the potent analog 19-S7 using the Luc-PANC-1–derived subcutaneous tumor model (Figure 9). The experimental timeline (Figure 9A) illustrates the treatment cycles used for both compounds. For each cycle, animals received daily treatments by oral gavage (25 mg/kg) for 5 continuous days, then were allowed to rest for 2 days without treatment, and a total of 4 cycles were administered. The same treatment dose of 25 mg/kg was utilized for both compound 19-S and 19-S7 after verifying the MTD for 19-S7 was the same 25 mg/kg observed for 19-S (Supplemental Figure 13). Following oral delivery, tumor-bearing animals treated with both 19-S and 19-S7 did not show adverse effects on or declines in body weight (Figure 9B). Tumor progression was determined by the bioluminescence photon flux (Figure 9, C and F, and Supplemental Figure 14), by direct tumor measurements (Figure 9, D and G), and by measurements of the final excised tumor mass (Figure 9, E and H). Both compounds inhibited tumor progression, as determined by the reduced bioluminescence photon flux from tumor cells (*P* = 0.0002 for 19-S and *P* < 0.0192 for 19-S7), reduced and stable tumor volumes (*P* < 0.0001 for both compounds), and reduced mass of the final excised tumors (*P* = 0.0010 for both compounds). In summary, compounds 19-S and 19-S7, administered orally as a single agent, show antitumor activity in a pancreatic tumor model with no observed adverse effects.

## Discussion

While TYMS inhibition has been a component of combination cytotoxic therapy for difficult-to-treat advanced cancers, current fluoropyrimidines and antifolate TYMS inhibitors are associated with induction of TYMS overexpression that confers drug resistance, resulting in limited long-term benefit and negligible cure rates in patients with advanced disease (1). For example, both the 5-FU prodrug analog capecitabine (1) and the compounded agent TAS-102 (trifluridine combined with tipiracil) result in the active FdUrd metabolite that exhibits the same potential for inducing TYMS overexpression as observed with 5-FU (1, 44, 45). Therefore, the pursuit of more effective TYMS inhibitors alone or in combination therapy is an important goal.

We have identified compounds that are structurally distinct from folic acid yet compete with the 5,10-mTHF cofactor required for the TYMS-catalyzed conversion of dUMP while maintaining the ability to inhibit DHFR, thus acting as multifunctional nonclassical antifolates. The three 19-S series compounds with dual TYMS/DHFR-inhibitory activity examined in this study show potent biological activity and are predicted to avoid the prototypic drug resistance arising from induction of TYMS overexpression. In addition, since these compounds are not classical folic acid analogs, they are not dependent on folate transporters or folate metabolism to achieve their full therapeutic potential. Therefore, these nonclassical antifolates provide therapeutic benefits of targeted therapy for patients with resistance to the standard classical antifolates related to these folate transport and metabolism pathways.

Our laboratory has previously reported that aberrant elevated levels of TYMS have oncogenic activity (12). Therefore, the ability to efficiently lower TYMS catalytic activity using a continuous maintenance regimen without the risk of inducing reciprocal feedback mechanisms such as TYMS overexpression is a key strategy to improve outcomes for patients with difficult-to-treat cancers (1). Maintenance-phase therapy using daily, weekly, or metronomic therapy could be an effective option for controlling TYMS activity, reducing side effects, and improving long-term outcomes. For this approach to be feasible, oral administration is preferred for improved patient logistics/compliance to maintain a continuous lower dose exposure compared with repeated high-dose bolus intravenous (IV) infusions. While current fluoropyrimidine treatment options can also be administered orally (44, 46), the induction of TYMS overexpression commonly observed in response to fluoropyrimidines limits the therapeutic benefits of inhibiting TYMS activity (1, 44). Maintenance therapy using antifolates was shown to be effective, as observed with PEM for non–small cell lung cancer, although such maintenance phase treatment still relies on IV infusions every 21 days because of poor oral absorption of the drug (42, 47, 48). Recent preclinical studies reported PEM bioavailability using PEM–bile acid conjugate complexes to enhance absorption (49–52). These complexes allow daily oral administration with antitumor activity and reductions in new blood vessel formation. While less frequent biweekly IV infusions had similar antitumor activity, there were negligible antiangiogenic properties observed (49, 52). These preclinical studies further highlight the potential benefits from antifolate therapy using frequent, low-dose treatment. We have now identified a family of orally administered nonclassical antifolates that optimizes inhibition of thymidylate biosynthesis with a favorable safety profile and extends survival in a pancreatic xenograft tumor mouse model and an *hTS/Ink4a/Arf^–/–^* genetically engineered spontaneous mouse tumor model. Elevated TYMS expression has been reported as a poor prognostic biomarker in many cancer subtypes, and somatic mutational or epigenetic silencing of the *INK4a/ARF* locus is one of the most common oncogenic events in human cancers. Therefore, the discovery of this family of nonclassical antifolates opens the door for new therapeutic approaches to offer potential benefit for a wide range of human tumors.

## Methods

### Cell culture.

PANC-1 and MIA PaCa-2 cells were purchased from the American Type Culture Collection and were grown in DMEM high-glucose medium (MilliporeSigma). BON cells, derived from a serotonin-secreting PanNET, were a gift from Kirk Ives (University of Texas, Galveston, Texas, USA) and were grown in DMEM-F12 (50/50) medium (Corning). CM cells derived from an insulin-secreting PanNET were a gift from Aldo Scarpa (University of Verona, Verona, Italy) and were grown in RPMI-1640 medium (MilliporeSigma). All were supplemented with 5 U/mL penicillin/streptomycin and 10% fetal bovine serum, except for CM cells that were supplemented with 5% fetal bovine serum. All cells were grown at a constant temperature of 37°C in a humidified atmosphere of 5% carbon dioxide and were routinely tested for mycoplasma contamination. PANC-1 and CM cells were further transduced with lentiviral firefly luciferase (Addgene, catalog 19785) and maintained in puromycin 8 μg/mL; luciferase-expressing cell lines were designated Luc-PANC-1 and Luc-CM. All cells were tested for mycoplasma before any experiment using a commercially available PCR-based detection kit (MilliporeSigma, catalog MP0025).

### Cell treatment, chemicals, and compound synthesis.

To prepare lysates, 0.5 × 10^6^ PANC-1 cells were seeded in 100 mm dishes in 10 mL DMEM high glucose, and 24 hours later, 5-FU (MilliporeSigma, catalog F6627) or 19-S were added at the specified concentrations. Cells were harvested after 72 hours, and cell pellets were stored at –80°C. For GI_50_ determination, 3,000 MIA PaCa-2 cells, 4,000 PANC-1 or CM cells, or 5,000 BON cells per well were plated in 96-well plates, and 16–20 hours after seeding, cells were treated with increasing doses of the indicated drug: 5-FU (MilliporeSigma, catalog F6627), MTX (MilliporeSigma, catalog A6770), PEM (LC Labs, catalog P-7177), 19-S, 19-S5, or 19-S7. After 72 hours, cell viability was assessed by reduction of MTS using Cell Titer 96 R Aqueous One Solution Cell Proliferation Assay Kit (Promega, catalog G8081), following manufacturer’s recommendations. Chemiluminescence output (integration time 1,000 ms) was measured on a SpectraMax M3 (Molecular Devices). Data were plotted in GraphPad Prism 9 to determine GI_50_ concentration. Chemical synthesis details, including characterization of each compound, can be found in the Supplemental section associated with this publication.

### Protein isolation.

Protein lysates were generated using RIPA buffer (Santa Cruz Biotechnology, catalog 24948A) for 20 minutes on ice, followed by 15 minutes of centrifugation at 13,000 rpm, at 4°C. Protein-containing supernatant was transferred to microcentrifuge tubes and stored at –80°C until further use. Protein was quantified using Bradford Assay (BioRad, catalog 5000006) following manufacturer’s recommendations; standard curves were generated with bovine serum albumin (Fisher, catalog BP 1600-100).

### Immunoblotting.

A total of 20 μg of total protein lysate was loaded per lane of 10% Tris Glycine Gel (Invitrogen, catalog EC6076). SDS-PAGE was run at 150 V for 1.5 to 2 hours. Proteins were transferred to nitrocellulose membranes using iBlot (Invitrogen, catalog IB3011002). Membranes were blocked with 5% nonfat dry milk (Lab Scientific, catalog M0841) in Tris-buffered saline supplemented with Tween 20 (0.1%) (TBS-T) for 45–60 minutes at room temperature (RT). Membranes were incubated on a plate shaker overnight at 4°C with TYMS-106 antibody in 1:300 dilution as previously described (12) or GAPDH (1:1,000, MilliporeSigma, catalog ABS16) diluted in blocking buffer. Membranes were washed extensively with TBS-T (4 times, for 5 minutes each), followed by incubation with horseradish peroxidase–conjugated secondary antibody goat anti–mouse IgG (BioRad, catalog 5000006) or goat anti-rabbit (BioRad, catalog 1706515) in blocking buffer 30–60 minutes at RT on a plate shaker. Membranes were washed extensively with TBS-T (minimum 4 times for 5 minutes). Signal was detected using West Pico Plus chemiluminescence substrate (Thermo Fisher Scientific, catalog 34580) following manufacturer’s recommendations. Membranes were developed using multiple films (Gene Mate, catalog F9023) processed in a Kodak X-Omat 2000A processor with exposures ranging from 2 seconds to 2 minutes. For protein-level quantification relative to loading control, densitometric analysis was performed by ImageJ software (NIH).

### Mice.

Mice were maintained within the University of Florida Cancer Genetics Research Center, and all animal experiments were done in accordance with approved protocols from the IACUC. NSG mice were bred at University of Florida animal facility. MTD studies were performed as described in Supplemental Methods. *hTS/Ink4a/Arf^–/–^* mice were generated by crossing hTS mice (generated in-house) (34) with *Ink4a/Arf*–null mice (NIH National Cancer Institute mouse repository, strain 01XB2) (35) to generate *hTS/Ink4a/Arf^–/–^* mice on a mixed FVB/129/Sv background (36). For survival studies, mice were sacrificed after reaching tumor growth parameters and/or discomfort as required by IACUC. Necropsy was performed for histologic evaluation and to score presence of tumors. Statistical analysis was performed using GraphPad Prism 9 (GraphPad Software), using the Kaplan-Meier method, and survival of groups was compared using log-rank (Mantel-Cox) test. Primer sequences for genotyping *hTS/Ink4a/Arf^–/–^* mice are provided in Supplemental Methods.

### Xenograft tumor models.

Luc-PANC-1 (5 × 10^6^) and Luc-CM (0.5 × 10^6^) cells were resuspended in 200 μL of PBS and injected subcutaneously or IP, respectively, into 6- to 8-week-old NSG mice. Tumor-bearing mice were imaged using Xenogen IVIS Lumina Bioluminescence Imaging System (PerkinElmer) every week after cell injection. Luc-PANC-1– and Luc-CM–injected mice were randomized based on slope of luciferase signal at 28 days and 24 days, respectively, and treatment was initiated. Mice received daily 19-S or 19-S7 at the indicated doses by IP injection or by oral gavage (PO), and vehicle control mice received corn oil. For all animals, body weight was recorded weekly, and Luc-PANC-1 tumor volume was measured weekly with a caliper. For scheduled sacrifice experiments, animals were euthanized after 4 weeks of treatment. For survival studies, animals were sacrificed when tumors reached 1,500 mm^3^ or when they showed any signs of ulceration. Harvested tumors were excised and weighed, then fixed in alcoholic formalin (67.5% ethanol, 10% formaldehyde 37%, 22.5% water) for pathology analysis. Tumor volume was calculated as volume = 0.52(length × width^2^).

### Drug preparation for treatment in vitro and in vivo.

For in vitro studies 50 mM stock solutions of PEM, MTX, 19-S, 19-S5, and 19-S7 were prepared in 100% DMSO and kept protected from light at RT. We prepared 50 mM FUrd and FdUrd in water. Working solutions were prepared dissolving the stock solution in tissue culture media. Stock solutions were stored at RT; no changes in activity were observed for up to 6 months, as determined by repeated viability assays performed using MIA PaCa-2 cell. For all compounds, the dry solid was protected from light and stored at –30°C.

For all in vivo animal treatments, we first prepared 5% DMSO stock solution of compound 19-S or 19-S7 and stored the stock solution at RT for up to 6 months. From this stock, the exact volume needed for daily in vivo delivery was then formulated into 95% corn oil (Mazola) at 2 different concentrations: 1.25 mg/mL (formulation 1) and 3.125 mg/mL (formulation 2). In order to know the volume to be administered to mice, we divided 200 μL (maximum volume to be delivered in mice IP or PO) by 25 g of mouse body weight. Therefore, 8 μL of formulation 1 or 2 multiplied by the weight of the mice was used to reach a dose of 10 or 25 mg/kg of body weight, respectively.

### Bioluminescence imaging.

Prior to bioluminescence imaging the region where tumors were located was shaved. Mice were then anesthetized with 2.5% isoflurane in O_2_, then administered the d-luciferin substrate (150 mg/kg in PBS) by IP injection. Following the injection of the d-luciferin substrate mice, were imaged using the Xenogen IVIS Lumina Bioluminescence Imaging System (PerkinElmer). The peak of luciferase photon flux was recorded 6 minutes after injection of the d-luciferin substrate. The total photon flux was analyzed and restricted to tumor region of interest using Living Image v2.60.1 software (Imaging Systems).

More information regarding human TYMS and DHFR preparation, tritium-based TYMS catalytic activity assay, competitive drug displacement assay, DHFR activity assay, initial computational screening for potential inhibitors, and molecular modeling of the proposed inhibition modes is provided in Supplemental Methods.

### Statistics.

Data analysis was performed by GraphPad Prism 9 (GraphPad Software), and *P* < 0.05 was considered statistically significant. Data were analyzed by 2-tailed Student’s *t* test for comparison between control and 19-S series analogs’ TYMS activity. For in vivo studies, tumor flux and tumor volume differences between controls and treated mice were calculated by 2-way ANOVA statistical test; unpaired 2-tailed Student’s *t* test with Welch’s corrections was used to compare tumor weight between controls and treated mice at endpoint. Control and 19-S–treated mice’s survival was compared by the log-rank (Mantel-Cox) test.

### Study approval.

All animal work was conducted under the approval of the University of Florida IACUC in accordance with federal, state, and local guidelines.

## Author contributions

MVG and AN performed animal studies, and MVG analyzed animal data and prepared all final figures. PED performed tritium catalytic activity assays. PCK and PED designed tritium catalytic activity assays and analyzed the data. RLB and JDL supervised, provided reagents for tritium assay, and edited the manuscript. LK designed and performed library screening. NGP and RWH performed synthesis of 19-S analogs, and NVB and RWH scaled synthesis of 19-S for animal studies. PCK, AN, and DS performed MTS assays. JA and RM purified TYMS and DHFR and measured DHFR catalytic activity. XL performed experiments with 5-FU–resistant tumor cell lines. CM and AR performed molecular docking simulation. MF, EN, and RPS analyzed histologic tissue sections. MZK, FJK, PCK, and MVG wrote the manuscript. MZK initiated the project, designed the experiments, supervised the studies, and analyzed the data. All authors read and approved the manuscript.

## Supplementary Material

Supplemental data

## Version 1 04/25/2023

In-Press Preview

## Version 2 05/22/2023

Electronic publication
