# Supplementary material for: First-in-class multifunctional TYMS nonclassical antifolate inhibitor with potent in vivo activity that prolongs survival
Source: JCI Insight. 2023 May 22;8(10):e158798. doi: 10.1172/jci.insight.158798 (PMC10386886; doi:10.1172/jci.insight.158798)
Supplement: Supplemental data [file jciinsight-8-158798-s010.pdf]

Supplemental Data

First-in-class multifunctional TYMS non-classical antifolate inhibitor with potent in vivo activity that prolongs survival

Maria V. Guijarro, Patrick C. Kellish, Peter E. Dib, Nicholas G. Paciaroni, Akbar Nawab, Jacob Andring, Lidia Kulemina, Nicholas V. Borrero, Carlos Modenutti, Michael Feely, Elham Nasri, Robert P. Seifert, Xiaoping Luo, Richard L. Bennett, Daniil Shabashvili, Jonathan D. Licht, Robert McKenna, Adrian Roitberg, Robert W. Huigens III, Frederic J. Kaye, and Maria Zajac-Kaye

Table of Contents

- **Supplemental methods**
- **Supplemental Figure 1.** Compound 19-S induces cytotoxicity in tumor cells.
- **Supplemental Figure 2.** Tritium assay optimization.
- **Supplemental Figure 3.** Compound 19-S does not induce resistance in PANC-1 and Mia PaCa-2 cells and shows a potent cytotoxic effect in 5-FU resistant PANC-1 cells.
- **Supplemental Figure 4.** Compound 19-S is not toxic at a dose of 25 mg/kg for IP treatment in NSG mice.
- **Supplemental Figure 5.** Bioluminescence imaging of Luc-PANC-1 derived tumors (IP treatment).
- **Supplemental Figure 6.** Compound 19-S (19-S) has a potent antitumoral effect and does not increase TYMS (TS) levels in Luc-PANC-1 derived tumors.
- **Supplemental Figure 7.** Bioluminescence imaging of Luc-CM derived tumors (IP treatment).
- **Supplemental Figure 8.** Compound 19-S (19-S) inhibits tumor growth and progression at 25 mg/kg PO in a Luc-CM disseminated tumor model.
- **Supplemental Figure 9**. Compound 19-S (19-S) is not toxic at a dose of 25 mg/kg for IP treatment in FVB/129/sv mice.
- **Supplemental Figure 10.** Compound 19-S (19-S) shows no toxicity at a dose of 10 mg/kg for IP treatment in *hTS/Ink4a/Arf*$^{-/-}$ mice.
- **Supplemental Figure 11.** Increased dUMP and 5,10-mTHF concentrations do not impact conversion of dUMP.
- **Supplemental Figure 12**. Pharmacophoric interaction used as a restraint for biased docking simulations.
- **Supplemental Figure 13.** Compound 19-S7 is not toxic at 25 mg/kg for PO treatment in NSG mice.
- **Supplemental Figure 14.** Bioluminescence imaging of Luc-PANC-1 derived tumors (PO treatment).
- **Supplemental Table 1.** Pharmacophoric interaction used as a restraint for biased docking simulations.
- **Supplemental Table 2.** Effect of substitution by Alanine of TS ligand binding site amino acids in 19-S compound affinity.

- **Supplemental Table 3**. Effect of substitution by Alanine of TS ligand binding site amino acids in 19-S compound affinity.

Chemical Synthesis, Characterization Data, and NMR Spectra

- **A)** Chemical Synthesis and Characterization Data
- **B)** NMR Spectra

Supplemental methods

Cell culture

H1048, Colo320, H630 and CCD18Co cells were purchased from ATCC. Cells were grown in RPMI 1640 supplemented with 5 U/ml Penicillin/Streptomycin and 10% fetal bovine serum at a constant temperature of 37ºC in a humidified atmosphere of 5% carbon dioxide and were routinely tested for mycoplasma contamination. PANC-1 cells resistant to 5-FU were generated in the laboratory by treating low passage PANC-1 cells with increasing concentrations of 5-FU (from 0.1 μM to 7.5 μM) in a 25 cm^2 flask. When cells were reaching 80% confluency, they were trypsinized and subcultured in higher concentration of 5-FU. PANC-1 cells resistant to 5-FU used in this report are resistant to 6.5 μM of 5-FU.

Viability assays

For GI_{50} determination, 3000 Colo320, H630 and CCD18Co cells per well were plated in 96 well plates and 16-20 hours after seeding, cells were treated with increasing doses of compound 19-S. After 72 hours, cell viability was assessed by reduction of MTT (3-(4,5-dimethylthiazol-2-yl)-2,5-diphenyltetrazolium bromide) using MTT Reagent (Invitrogen, Cat # M6494), following manufacturer's recommendations. Chemiluminescent output (integration time 1000 ms) was measured on a SpectraMax M3 (Molecular Devices). Data was plotted in GraphPad Prism 9 to determine GI_{50} concentration.

Cell count assays

500000 PANC-1 and MiaPaCa-2 cells were plated in 75 cm^2 flasks in DMEM high glucose supplemented with 5 U/ml Penicillin/Streptomycin and 10% fetal bovine serum. Cells were treated with 10 μM of either compound 19-S or 5-FU. Once a week for 7 weeks, cells were trypsinized with Trypsin-EDTA 0.05% (Gibco) and counted using Cellometer Auto T4 (Nexcelom Bioscience). 500000 cells were re-seeded in a new 75 cm^2 flask in the appropriate treatment. Data was plotted in GraphPad Prism 9.

Clonability assay

500 PANC-1 cells resistant to 6.5 μM 5-FU cells were seeded in 6 well plates by triplicate in media containing either 6.5 μM 5-FU or 8.35 μM compound 19-S. Media with treatment was replaced every week. After 21 days, cells were washed with PBS, fixed with Methanol for 20 minutes and stained with 1% Crystal violet (in PBS) for 20 minutes. After extensive washing, plates were let dry and colonies were counted and plotted in GraphPad Prism 9. Images were taken using scanner Epson Perfection V700 photo (Epson).

Maximum tolerated dose (MTD)

For IP treatment, 6-8 weeks old NSG or FVB/129/sv (*Ink4a/Arf* $^{+/+}$ and *Ink4a/Arf* $^{-/-}$) mice were IP injected with 10, 25, 50 or 100 mg/kg of compound 19-S either weekly or biweekly (depending on the experiment) or 5, 10, 25 or 50 mg/kg of 5-FU daily during 5 consecutive days. After 3 treatment cycles, animals rested (no treatment) for one more week before euthanasia. Body weight and physical status of all mice were closely monitored. Tissues were collected and fixed in alcoholic formalin to test for toxicity.

For PO treatment, 6-8 weeks old NSG were administered PO 25 or 50 mg/kg of compound 19-S7 daily during 5 consecutive days for 3 weeks. After 3 treatment cycles, animals rested (no treatment) for one more week before euthanasia. Body weight and physical status of all mice were closely monitored. Tissues were collected and fixed in alcoholic formalin to test for toxicity.

hTS/Ink4a/Arf $^{-/-}$ mice genotyping

Genotype analysis in tail snips was performed by standard PCR analysis using human TS-specific primers (1) as well as a set of primer pairs designed to score for the wild-type, heterozygous, or homozygous *Ink4a/Arf* genotype. The sequences for *hTS* primers are: NCI2 5'-ATGCCCTCTGCCAGTTCTATGTGG-3' and H2I 5'-TAGAAGGCACAGTCGAGG-3'; and *Ink4a/Arf* locus primers are as follows: #I001, 5'-GTGATCCCTCTACTTTTTCTTC-3', #I002, 5'-CGGAACGCAAATATCGCAC-3', and #I003, 5'-GAGACTAGTGAGACGTGCTAC-3'. I001/I003 detects a 313 bp band for the knockout and I001/I002 a 278 bp band for the wild type.

Histopathology

Specimens were fixed in alcoholic formalin for 48h and then transferred to 70% ethanol, processed and embedded in paraffin. Tissues were serially sectioned and stained by conventional H&E. Slides were interpreted by blinded certified pathologists: Robert P. Seifert (Hematopathology Program Director and Clinical Assistant Professor), Elham Nasri (Bone and soft tissue Pathology and Clinical Assistant Professor) and Michael Feely (Gastrointestinal/liver and genitourinary Pathologist and Clinical Assistant Professor) at Anatomic Pathology Department at UF, Gainesville, FL.

Tumor lysate preparation

A small piece of frozen tumor (stored at -80 °C) in RIPA buffer with phosphatase and proteinase inhibitors (Santa Cruz, sc-24948) was homogenized using Omni general laboratory homogenizer (GLH). Tumor was then centrifuged for 20 min at 12000 rpm and 4 °C to obtain whole-cell lysates that was transferred to a new tube. Protein concentration was measured using Bradford Assay and 20 μg of total protein lysate was loaded as described in Methods.

Tritium based TYMS catalytic activity assay

The catalytic activity of TYMS was determined by measuring the release of [^{3}H] from [5-^{3}H]dUMP (ViTrax, Cat # VT122) resulting from the conversion of dUMP to dTMP as previously described (2, 3). The reaction conditions were optimized to determine the amount of TYMS protein needed to establish the range of TYMS catalytic activity assay and confirm the background signal from negative control reactions performed without TYMS or without the 5,10-methylene tetrahydrofolate (5,10-mTHF) cofactor (Supplemental Figure 2 and Supplemental methods). Reactions were performed in 50 mM KH_2PO_4 (Sigma Aldrich, Cat # P0662) at pH 7.2 using 2 µg of purified human TYMS. Reactions were performed in a final volume of 200 µL with 100 mM β-mercaptoethanol (Sigma Aldrich, Cat # M7522), 40 µM dUMP (Sigma Aldrich, Cat # D3876), 100 µM 5,10-mTHF, and the [5-^{3}H]dUMP tracer (ViTrax, Cat # VT122) accounting for 0.06% of the final dUMP concentration in the reaction. The 5,10-mTHF used in the reaction was separately generated from tetrahydrofolic acid (Sigma Aldrich, Cat # T3125) by dissolving the solid in 10 mM KH_2PO_4 with 143 mM β-mercaptoethanol (1:100 from 14.3 M pure liquid), and 6.35 mM formaldehyde (Sigma Aldrich, Cat # 47608) to yield a 100 mM stock solution of 5,10-mTHF. Reactions were initiated by the addition of the dUMP substrate and 5,10-mTHF cofactor, then incubated at 37°C for 30 minutes. Reactions performed in the presence of compound 19-S series analogues, FUrd (Sigma, Cat # F5130), FdUrd (Sigma, Cat # F0503), PEM (Sigma Aldrich, Cat # PHR1596), or MTX (Sigma Aldrich, Cat # CS0340) were pre-incubated with each drug for 30 minutes before the reaction was initiated by the addition of the dUMP substrate and 5,10-mTHF cofactor. Reactions were terminated by the addition of 100 µL of ice-cold 20% trichloroacetic acid (Sigma Aldrich, Cat # T6399), then excess [5-^{3}H]dUMP was removed by the addition of 200 µL of an albumin-coated activated charcoal solution. The albumin-coated activated charcoal solution was prepared by mixing 10 g of acid-washed activated dextran coated charcoal (Sigma Aldrich, Cat # C6241) with 2.5 g of bovine albumin (Sigma Aldrich, Cat # A1933) in 100 mL of H_2O. The suspension was incubated at room temperature for 30 minutes and then centrifuged for 30 minutes at 10,000 x g. Then 150 µL of the supernatant was collected and added to 5 mL of ultima gold liquid scintillation cocktail (Perkin Elmer, Cat # 6013326, lot: 77-19085), and scintillation measurements were perform using a liquid scintillation counter (Beckman Coulter LS6500) to determine [^{3}H]H_2O levels in each sample. For all experiments, positive control reactions were performed with no drug representing 100% TYMS activity, and negative

control reactions were performed with no TYMS enzyme or no 5,10-mTHF substrate representing 0% TYMS activity. All assays were performed in biological duplicate and technical duplicate. Results were analyzed using GraphPad Prism 9 (GraphPad Software, USA).

Optimization of tritium based TYMS catalytic assay

The tritium based TYMS catalytic activity assay was optimized to determine the amount of purified TYMS protein for each reaction. Additionally, these data showing the actual CPM values obtained highlight the range of the assay with low background signal observed when either TYMS or 5,10-mTHF is absent in the reaction compared to control reaction with all components and 2 μg of TYMS protein (Supplemental Figure 2). The reactions performed without 5,10-mTHF resulting in similar signal as reactions performed without the TYMS protein (Supplemental Figure 2) demonstrate the dependence of the reaction on the 5,10-mTHF cofactor and how the increased signal is solely due to the conversion of dUMP by TYMS and not influenced by other factor like potential oxidation, as observed in assays using the conversion 5,10-mTHF to THF as a readout for the TYMS reaction. Due to the sensitivity and range of the tritium based assay a large decrease in dUMP conversion is observed in the concentration range where the TYMS dimer begins transitioning to the inactive monomer form (Supplemental Figure 2), this concentration dependent TYMS dimer to TYMS monomer transition is not detectable at the higher concentrations required for absorbance based assays.

First the hTYMS catalytic activity of different amounts of hTYMS was determined by measuring the release of [^{3}H] from [5-^{3}H]dUMP (ViTrax) during the conversion of dUMP to dTMP (Supplemental Figure 2A, B). The total volume for each reaction was 200 µL and reactions were performed using purified hTYMS ranging from 0.00625 ug to 100 ug (Supplemental Figure 2A, B). All reactions were perfomred in 50 mM KH_2PO_4 (Sigma Aldrich, P0662) at pH 7.2 with 100 mM β-mercaptoethanol (Sigma Aldrich, M7522), 40 µM dUMP (Sigma Aldrich, D3876), 100 µM 5,10-methylene tetrahydrofolate, and the [5-^{3}H]dUMP tracer (ViTrax, VT122) accounting for 0.06% of the final dUMP concentration in the reaction. Each reaction was incubated at 37 °C for 30 minutes, then the reaction was terminated by the addition of 100 µL of ice-cold 20% trichloroacetic acid (Sigma Aldrich, T6399). Excess [5-^{3}H]dUMP was removed by the addition of 200 µL of an albumin-coated activated charcoal solution (prepared by mixing 10 g of acid-washed activated dextran coated charcoal (Sigma Aldrich, C6241), 2.5 g of bovine albumin (Sigma Aldrich, A1933) and 100 mL of water. The suspension was incubated at room temperature for 30 min and then centrifugated for 30 min at 10,000 x g. Then 150 µL of the supernatant was collected and added to 5 mL of ultima gold liquid scintillation cocktail (Perkin Elmer, 6013326, lot: 77-19085), and scintillation measurements were performed using a liquid scintillation counter (Beckman Coulter LS6500) to determine [^{3}H] H_2O levels in each sample. For each 200 µL reaction, 2 µg of purified TYMS protein was selected for future assays. Then reactions were performed with all components except the 5,10-mTHF cofactor, all reaction components except 2 µg of TYMS protein, and a positive control with

all components including 2 μg TYMS protein to show the range of the assay, the background signal, and the dependence on 5,10-mTHF (Supplemental Figure 2C). To determine the signal from the [5-^{3}H]dUMP tracer used in each reaction the same amount of the dUMP substrate with the [5-^{3}H]dUMP tracer accounting for 0.06% of the final dUMP concentration was added directly to 5 mL of ultima gold liquid scintillation cocktail (Supplemental Figure 2C). The resulting signal was over 8-fold higher than the positive control reaction demonstrating all the [5-^{3}H]dUMP tracer is not converted in the reaction and demonstrates the efficiency of the albumin-coated activated charcoal solution removing the [5-^{3}H]dUMP that was not converted during the reaction. All reactions were performed in biological duplicate and technical duplicate. Results were analyzed using GraphPad Prism 9.

Competitive drug displacement assay

The tritium based TYMS catalytic activity assay was modified to determine if TYMS inhibition from 19-S, 19-S5, and 19-S7 was the result of competitive binding with either the dUMP substrate or the 5,10-mTHF cofactor. The competitive drug displacement assay utilized the standard reaction conditions for the tritium-based TYMS catalytic activity assay including 40 μM dUMP (Sigma Aldrich, Cat # D3876) with the [5-^{3}H]dUMP tracer (ViTrax, VT122) accounting for 0.06% of the final dUMP concentration and 100 μM 5,10-methylene tetrahydrofolate (defined as 1x dUMP and 1x 5,10-mTHF). Concentrations of total dUMP and 5,10-mTHF were then changed relative to these 1x concentrations to concisely indicate the changes in either the substrate or cofactor concentration. The highest 5x concentration of 5,10-mTHF was 500 μM which was limited by the solubility of the cofactor generated from THF. The highest 5x concentration of total dUMP was not limited by solubility, although increases were performed in the same increments as 5,10-mTHF for comparison and 0.06% of the [5-^{3}H]dUMP tracer was maintained at all concentrations. The remaining components in each 200 μL reaction were consistent in all reactions and included 2 μg of purified human TYMS in 50 mM KH_2PO_4 (Sigma Aldrich, Cat # P0662) and 100 mM β-mercaptoethanol (Sigma Aldrich, Cat # M7522). Control antifolate inhibitor PEM (Sigma Aldrich, Cat # PHR1596) was performed for reference of a TYMS specific classical antifolate and to illustrate the increased activity as a result of displacement with the increasing ratios of the 5,10-mTHF cofactor. While FdUrd is a known inhibitor targeting the dUMP binding site, its binding results in an irreversible covalent bond rather than the reversible binding required for the competitive drug displacement assay, although, increasing dUMP did not show drug displacement.

Human TYMS and DHFR preparation

Human Thymidylate Synthase (hTYMS) and human Dihydrofolate Reductase (hDHFR) were both expressed in BL21 (DE3) *E. coli* cells, using a standard BL21 transformation protocol, with the expression vector pQE80L-hTYMS (gift from Maria Paola Costi, University of Modena and Reggio Emilio, Italy) and pET100/D-TOPO (Invitrogen, Cat # K10001), respectively. Both vectors contained an N-terminal 6x Histidine tag attached to either hTYMS or hDHFR. After bacterial transformation, cells

were transferred to a Luria Broth culture medium for overnight growth at 37°C in presence of ampicillin and cell growth was measured via OD600 absorbance. Protein expression was induced via addition of 1 mM Isopropyl β-D-1-thiogalactopyranoside (IPTG) once OD600 reached 0.6, approximately 4 hr. After 3 hours incubation in the presence of IPTG cells were pelleted via centrifugation (4000 RPM for 10 minutes in a Beckman J20 rotor). The resulting pellet(s) were resuspended in Wash Buffer 1 (WB1) (20 mM NaH_2PO_4, 30 mM NaCl, 20 mM Imidazole, pH 7.8) and lysed via a microfluidizer (LM10, Microfluidics) set to 18,000 PSI. The cell lysate was centrifuged for 75 minutes at 12,000 RPM in a Beckman J10 rotor and subsequent supernatant was collected and passed through a 0.8 μm syringe filter (Millipore Sigma, Burlington, MA, CAT# 3 SLAA0335B) and loaded onto a HisTrap HP (GE) 5 mL column. The column was placed on AKTA pure 25 L1 FPLC (Cytiva, Marlborough, MA) and subjected to an imidazole gradient to elute the enzyme using WB1 and hTYMS Wash Buffer 2 (hTYMSWB2) (20 mM NaH_2PO_4, 30 mM NaCl, 500 mM Imidazole, pH 7.4) and hDHFR Wash Buffer 2 (hDHFRWB2) (20 mM NaH_2PO_4, 30 mM NaCl, 200 mM Imidazole, pH 7.8). Fractions collected were then subjected to 10% SDS-PAGE to identify hTYMS and hDHFR high purity fractions which were pooled and resuspended in hTYMS Storage Buffer (hTYMSSB) (20 mM NaH_2PO_4, 30 mM NaCl, pH 7.4) and hDHFR SEC Buffer (hDHFRSEC) (10 mM Tris, 1 mM EDTA, pH 8.0) using AMICON Ultra-15 Centrifugal Filters (Millipore Sigma, Burlington, MA, Cat # UFC901008) respectively. hDHFR was subjected to further purification via size exclusion chromatography utilizing a HiLoad 16/600 Superdex 75pg column (Millipore Sigma, Burlington, MA, Cat # 28989333). The major peak fractions were collected, and buffer exchanged into hDHFR Storage Buffer (hDHFRSB, 10 mM Tris, 1 mM EDTA, 1 mM DTT, 50% Glycerol, pH 8.0). Final purity was determined via 10% SDS-Page and concentration was determined by UV/Vis spectroscopy at 280 nm. hTYMS was stored in hTYMSSB at 4°C. hDHFR was stored in hDHFRSB at -20°C.

DHFR activity assay

Compound 19-S series analogues activity screen against DHFR activity was determined by using a DHFR assay kit (Sigma Aldrich, Cat # CS0340) as described by the manufacturer. 0.1-unit DHFR enzyme (Sigma, Cat # D6566) was added to 1x assay buffer (Sigma, Cat # A5603), 19-S series analogues or control drugs, 6 μL NADPH Cat # N6505 solution and 5 μl of dihydrofolic acid (DHFR substrate) (Sigma, Cat # D7006). The reaction was then read on the spectrophotometer at 340 nm at 22 ºC every 15 seconds for 2.5 minutes.

Initial computational screening for potential inhibitors

A National Cancer Institute (NCI) library of 139,735 compounds was obtained from the ZINC database (zinc.docking.org and https://pubs.acs.org/doi/abs/10.1021/acs.jcim.5b00559), and redundant structures, and molecules with molecular weight under 200 Da were then removed. Utilizing the established NCI library of compounds provides the ability to rapidly acquire compounds for in vitro

testing to validate cancer cell cytotoxicity and TYMS inhibition. We used DOCK6.5 with AMBER scoring (https://onlinelibrary.wiley.com/doi/abs/10.1002/jcc.23905) to computationally screen this curated library within the constraints of the selected docking site in TYMS. Compounds were each positioned into the target region in 1000 orientations and ranked based on their predicted energy scores. Molecular docking experiments were performed by using UF High Performance Computing Cluster resources. The top 1000 hits were visualized in PyMOL (www.pymol.org) and manually checked for consistency of molecular docking predictions and overall fit. Using the ZINC database and ALOPGS (http://www.vcclab.org/lab/alogps/), compounds with favorable lipophilicity (logP ≤ 5), solubility (logS ≥ -4), and polar surface area (PSA) under ~140Å^2 were selected, as these parameters are shown to be good predictors for absorption, distribution and oral bioavailability (4). The top 26 compounds from this final curated set were ordered from NCI and were tested, as described in the main text.

Molecular Modeling of the proposed inhibition modes

The 19-S ligand was built and optimized using the VMD Molefacture plugin (5). PDB IDs 1HVY and 4KAK were used as initial receptor structures for TYMS and DHFR, respectively. Only protein residues and cofactors (dUMP and NADPH in each case) were kept, and all structural waters were removed. The receptor structure was prepared following the standard AutoDock protocol (6) using the *prepare_receptor4.py* script from AutoDock Tools. All non-polar hydrogens were merged, and Gasteiger charges and atom types were added. The ligand PDBQT was prepared using the *prepare_ligand4.py* script and modified to include de AC atom with *prepare_bias.py* script (7), both available in AutoDock Tools. The grid size and position were chosen to include the whole ligand-binding site (including all protein atoms at a distance lower than 5 Å from all crystallized ligands). The spacing between grid points was set at 0.375 Å. AutoDock Bias protocol (7) was applied to perform a biased docking experiment taking into consideration previous information from the targets. Briefly, considering the main interactions (Supplemental Table 1) of the co-crystallized ligands (D16 and 06U ligands from PDB IDs 1HVY and 4KAK, respectively), a hydrogen bond acceptor pharmacophoric restraint was added by modifying their respective energy grids (OA and NA maps) using *prepare_bias.py* script. Additionally, aromatic biases were handled by creating a new AC atom type in the center of aromatic rings and placing the grid (AC map) modification in the location of ligand interactors (Supplemental Table 1), a strategy thoroughly validated by Arcon et al (8, 9).

For each system, 100 different docking runs were performed, and the results were clustered according to the ligand heavy atom RMSD using a cut-off of 2 Å. The Lamarckian Genetic Algorithm (LGA) parameters for each conformational search run were kept at their default values (150 for initial population size, 1×10^7 as the maximum number of energy evaluations, and 2.7×10^4 as the maximum number of generations). The docking results for 19-S were further analyzed by visual inspection.

Images of the molecules were prepared using the Visual Molecular Dynamics (VMD) program (5). The 2D diagram of the protein-ligand interactions was generated using the PoseView server (10).

Alanine Mutational Analysis

In silico Alanine mutational analysis (IAS) was performed using the structure package from the pyFodlX library (11) which allows the inclusion of ligand during the estimation of the difference in Gibbs free energy (ΔΔG) due to the substitution of a particular amino acid by Alanine. The parameters for the 19-S compound were built using the paramx package.

Binding Gibbs free energy (ΔΔG) was estimated by substituting predicted amino acids to alanine (A) (Supplemental Table 2 and 3). Spontaneous protein-ligand binding occurs only when the change in ΔΔG of the system is negative. Hence, a positive ΔΔG (over 1 Kcal/mol) using IAS indicated a significant involvement of the residue in the binding. When Asp218 (D218A) in TYMS was substituted by A, the ΔΔG was +2.31 Kcal/mol, suggesting the involvement of Asp218 on binding the aminopyrimidine group of 19-S through a hydrogen bond. Similar impact in binding was found with Trp109 (W109A) and Phe225 (F225A) that rendered ΔΔG of +1.79 and +1.97 Kcal/mol, respectively (Supplemental Table 2). In addition, we performed mutational analysis to address DHFR binding to 19-S and observed that Glu30 (E30A) and Phe34 (F34A) in DHFR rendered a ΔΔG of +2.52 Kcal/mol and +1.80 Kcal/mol, respectively, suggesting their important involvement in binding between 19-S and DHFR. ΔΔG for Val8 was +0.37 Kcal/mol, however, Val8 may be still contributing to binding to 19-S since pyFoldX software treats the backbone as a rigid body and only optimizes and measures the energy contribution of sidechains. Since Val8 interacts with the ligand through backbone oxygen, there are not significant differences when you change Val8 for Ala8 and thus Ala8 may be contributing significantly to binding energy (Supplemental Table 3). As expected, the hydrogen bond and the aromatic residues have a key role in the union to 19-S while the aliphatic amino acids contribute mainly to the stability of the cavity. These results are in agreement with previous analysis reported for TS (12) and DHFR (13) in which phenylalanine in combination with negatively charged amino acids (aspartic and glutamic acid for TS and DHFR respectively) play a main role in ligand stabilization.

Supplemental Figures

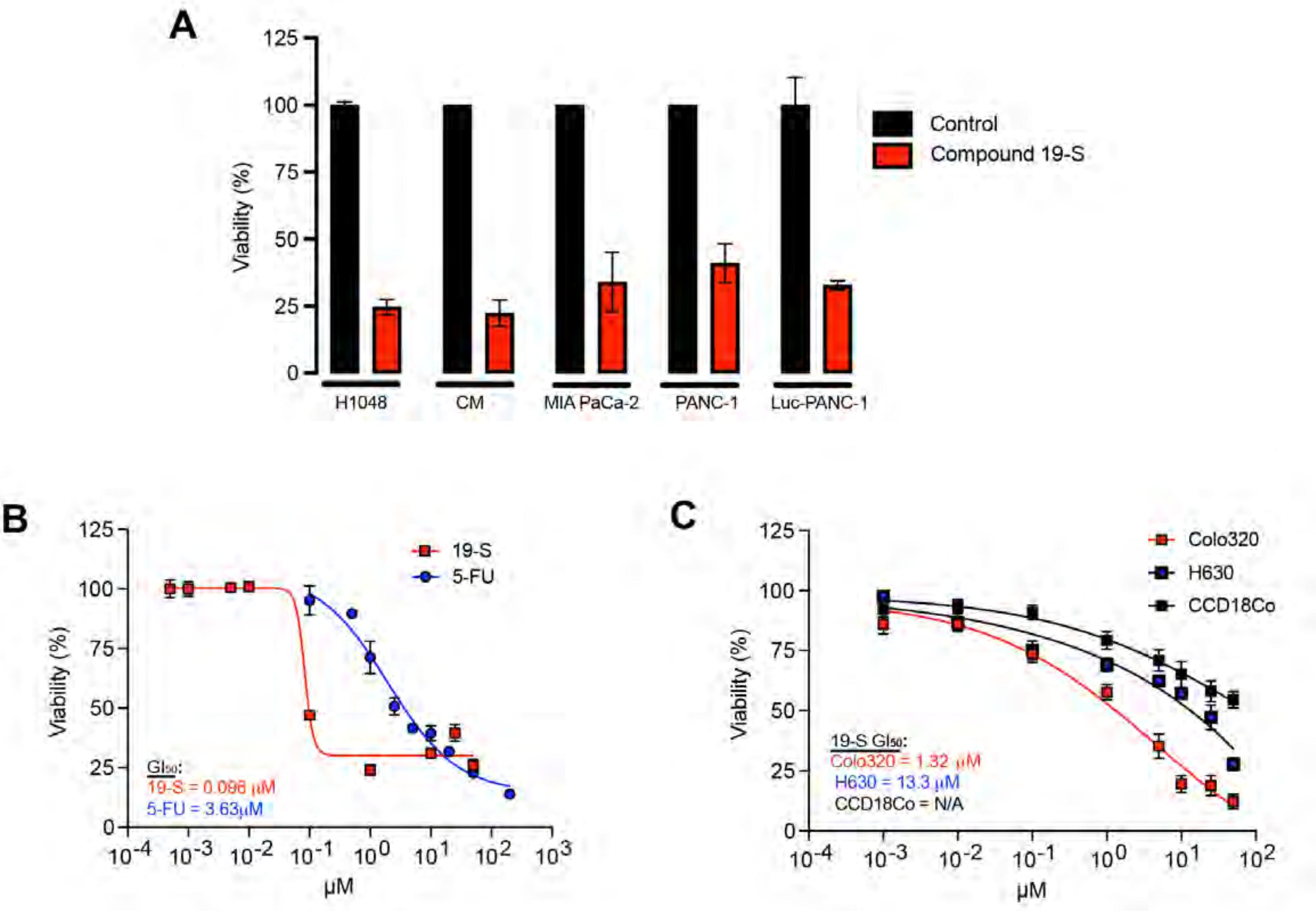

Supplemental Figure 1. Compound 19-S induces cytotoxicity in tumor cells. (**A**) Viability assay screen performed in a panel of 5 cancer cell lines including H1048 (small cell lung cancer), CM (pancreatic neuroendocrine tumor), MIA PaCa-2 and PANC-1 (pancreatic ductal adenocarcinoma) and Luc-PANC-1 (pancreatic ductal adenocarcinoma cells expressing firefly luciferase).Initial viability assay screens were performed with single drug concentration (10 μM) or DMSO control (0.25%). Each cell line was treated with either compound 19-S (right/red bars) or DMSO control (left/black bars) for 72 hours. The threshold of 10 μM was selected since compounds showing under 50% viability at 10 μM would indicate greater cytotoxicity than known TS inhibitor 5-FU and present a promising lead candidate for further study. Data is presented as mean ± SD of 3 technical replicates except for PANC-1 that shows mean ± SD of 3 experiments with 3 biological replicates each. (**B**) Viability assay of H1048 small cell lung cancer cell line comparing TS inhibitor 5-FU to compound 19-S after 72h drug incubation. Data is expressed as mean ± SD of 3 independent experiments, n = 3 technical replicates per concentration. (**C**) Viability assays of colorectal Colo320 and H630 cancer cell lines compared to normal colon CCD18Co fibroblast cell line, treated with compound 19-S for 72h. Data is expressed as mean ± SD of 3 independent experiments, n = 3 technical replicates per concentration.

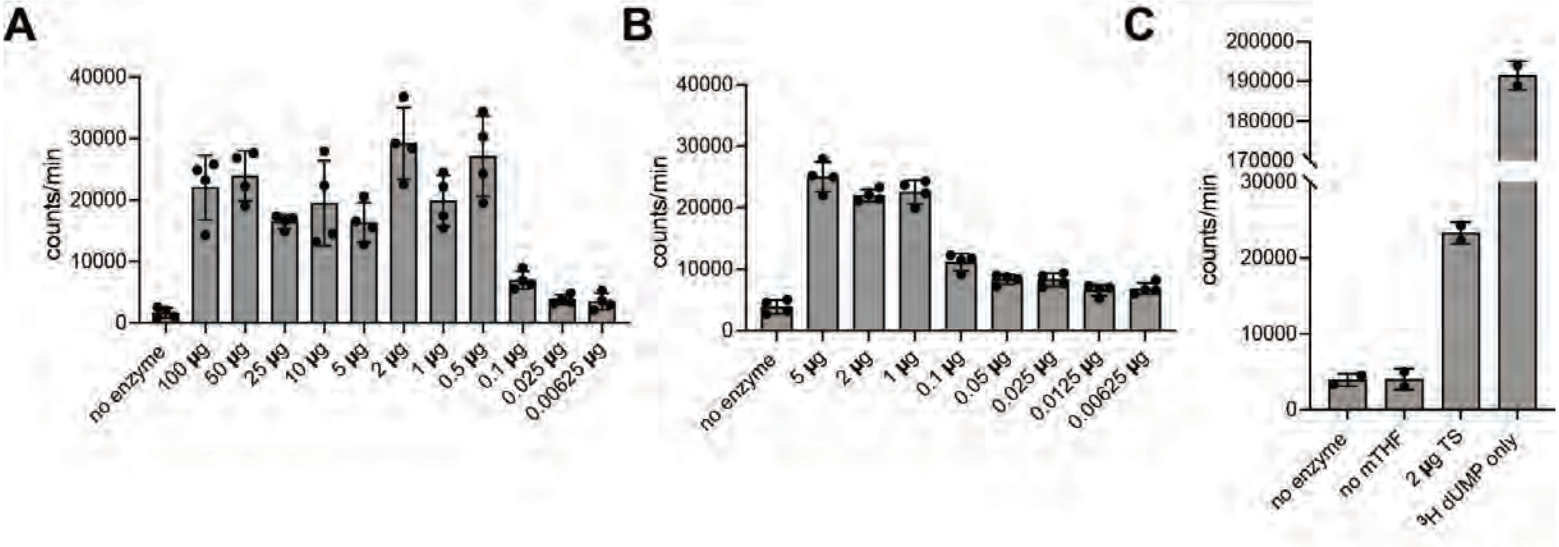

Supplemental Figure 2. Tritium assay optimization to detect TYMS (TS) catalytic activity. 2 µg is the optimal amount of purified human thymidylate synthase (hTS) to be used as a control in the tritium-based catalytic activity assay. (**A**) hTS catalytic activity was determined using a range of purified hTS, from 0.00625 µg to 100 µg. hTS catalytic activity was around 20,000 counts/minute between 100 µg and 0.5 µg hTS, but activity dropped one-fold when using from 0.1 µg to 0.00625 µg hTS. (**B**) hTS catalytic activity was determined, as in A, using a smaller hTS µg range (from 5 µg to 0.00625 µg). hTS catalytic activity dropped one-fold when using 0.1 µg to 0.00625 µg purified hTS, as previously observed. (**C**) No hTS catalytic activity is observed when no 5,10-methelyne tetrahydrofolate (mTHF) was present in the reaction in the tritium-based catalytic assay. This confirmed the importance of mTHF as methylene and hydride donor in the methylation of deoxyuridine monophosphate (dUMP) in each reaction carried. In addition, we determined the number of counts per minute present in tritiated dUMP added to each reaction. This was determined by adding 20 µL of diluted tritiated dUMP to 5 mL of liquid scintillation cocktail which was then measured on the liquid scintillation counter. Data presented as mean ± SD of two independent experiments, *n* = 2 technical replicates per experiment.

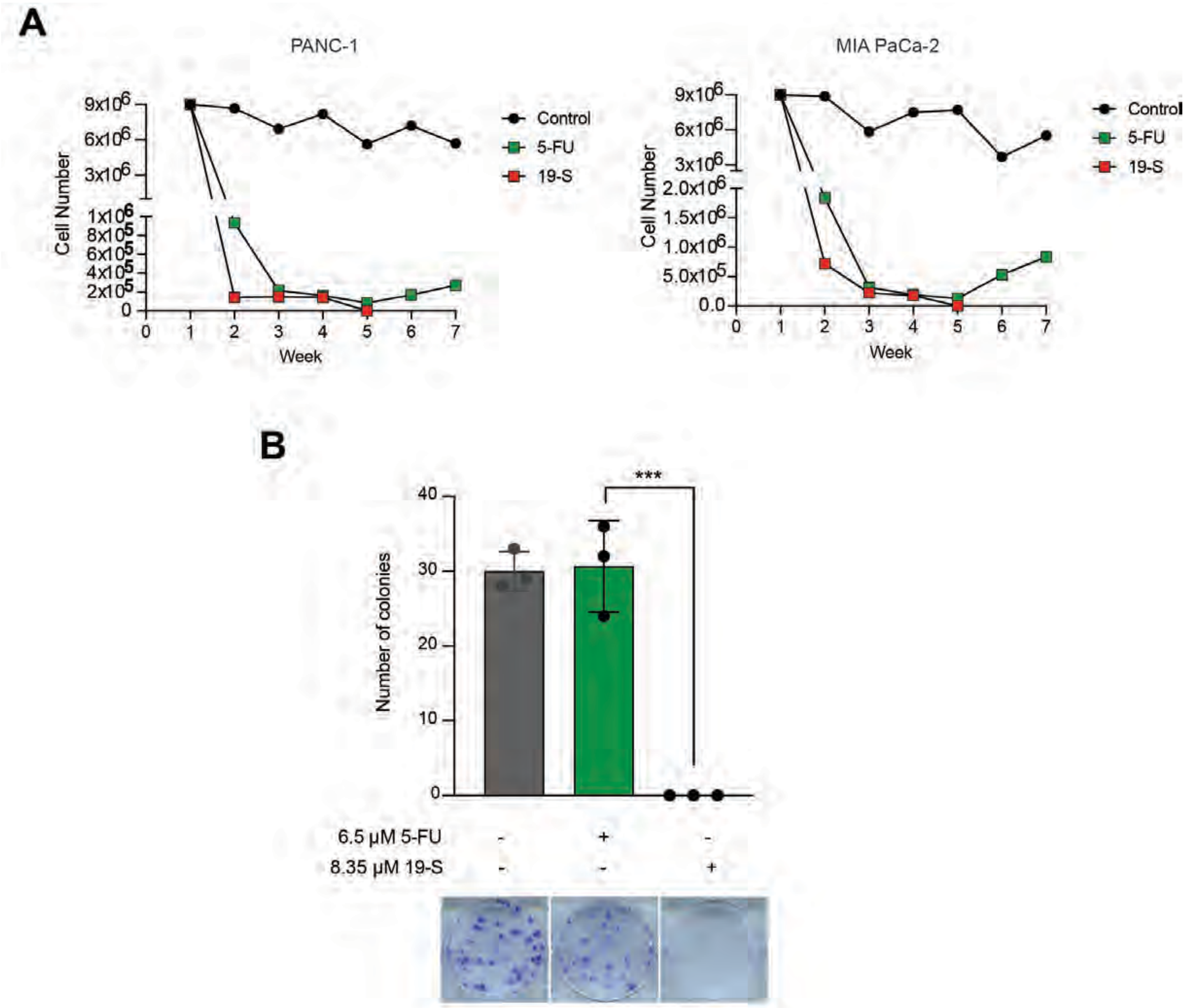

Supplemental Figure 3. **Compound 19-S does not induce resistance in PANC-1 and MIA PaCa-2 cells and shows a potent cytotoxic effect in 5-FU PANC-1 resistant cells.** (**A**) Cell counts of parental PANC-1 and MIA PaCa-2 cells treated with 10 μM of either compound 19-S or 5-FU compared to non-treated cells over 7 weeks. (**B**) Colony forming assay of PANC-1 cells resistant to 6.5 μM of 5-FU treated with either 6.5 μM 5-FU or 8.35 μM 19-S compared to untreated controls after 21 days of treatment. Data is expressed as mean ± SD of triplicates. Statistical analysis was performed using unpaired 2-tailed t-test ***P = 0.0010. A representative picture of the treated wells is shown per condition.

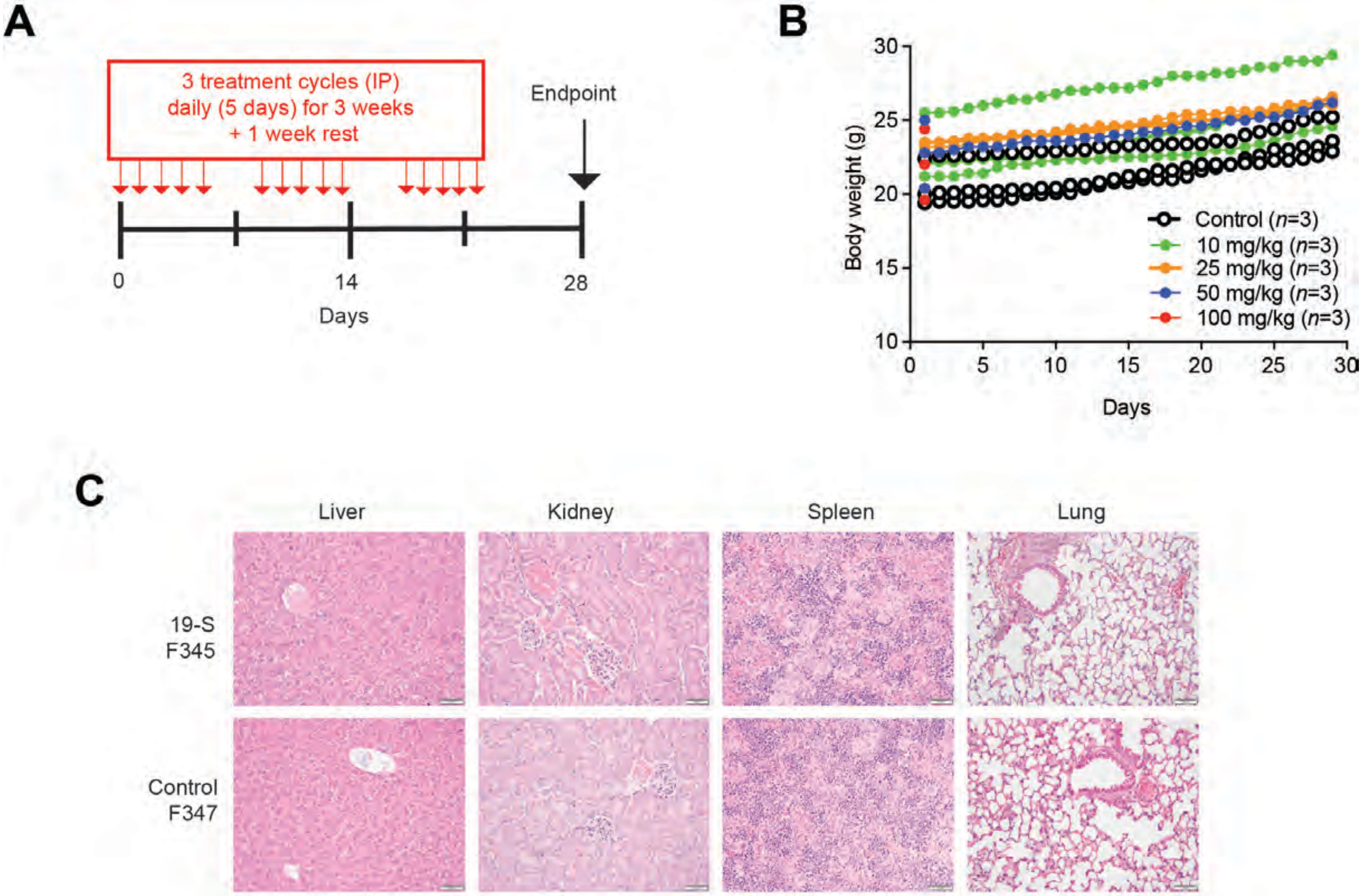

Supplemental Figure 4. Compound 19-S is not toxic at a dose of 25 mg/kg for IP treatment in NSG mice. (**A**) Timeline indicating IP treatment schedule for compound 19-S in NSG mice to determine maximum tolerated dose (MTD). Compound 19-S was administered IP 5 days a week for 3 weeks and animals rested (no treatment) for one extra week before experimental endpoint. (**B**) Effect of compound 19-S [10 mg/kg (n = 3), 25 mg/kg (n = 3), 50 mg/kg (n = 3) and 100 mg/kg (n = 3)] on BW following the treatment schedule shown in (A), compared to vehicle control (n = 3). Individual mice weight is represented. All 100 mg/kg treated mice were found dead after 1st dose. Out of n = 3 animals treated with 50 mg/kg, 2 were found dead after 1st dose and one animal reached endpoint. Mice treated with 10 and 25 mg/kg reached endpoint. (**C**) Representative pictures of H&E stained FFPE sections of liver, kidney, spleen and lung from compound 19-S (F347) compared to vehicle control treated NSG mice (F345). No evidence of drug tissue injury was found in any of the organs examined from 19-S treated mice compared to control mice. Scale bar represents 50 μm.

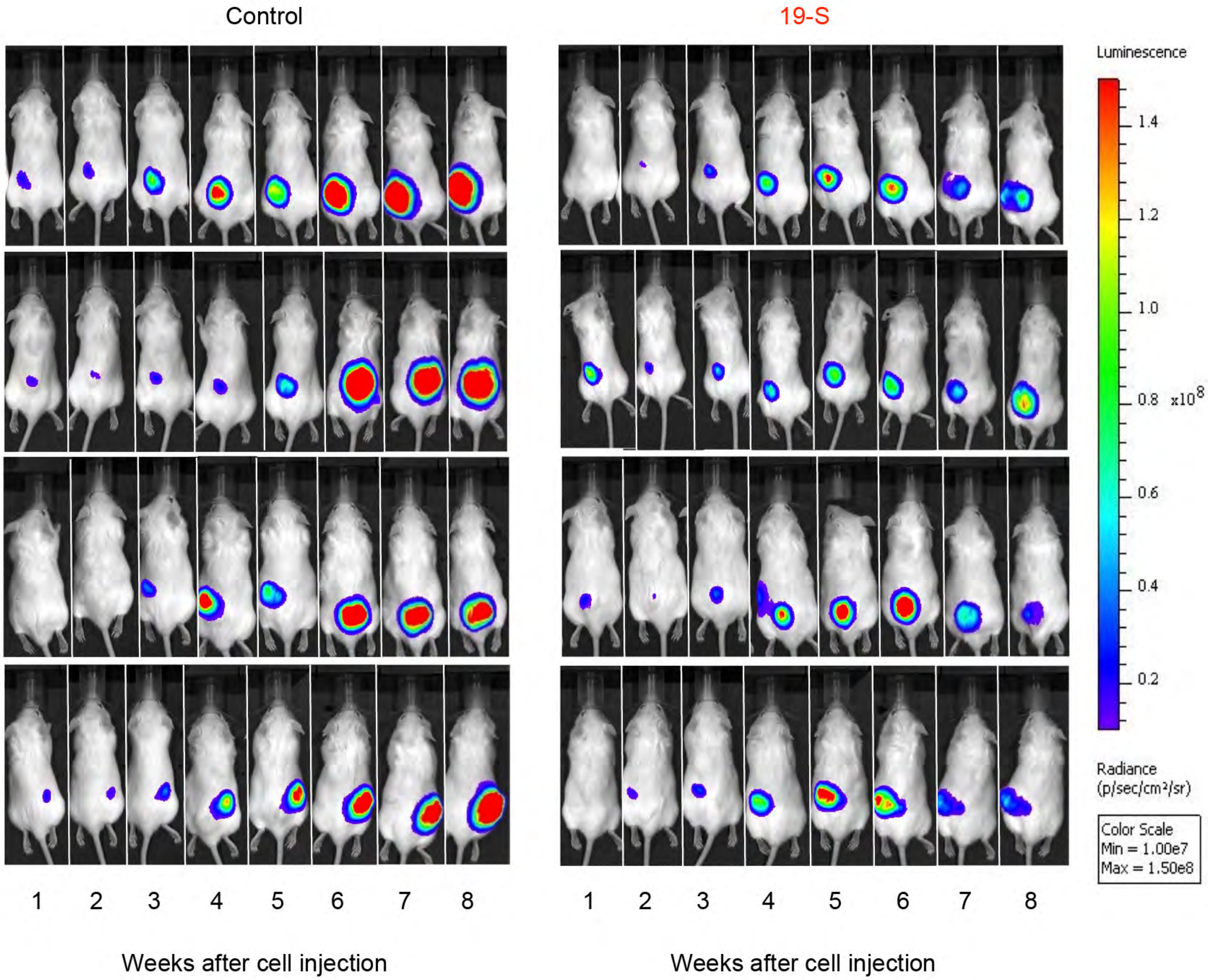

Supplemental Figure 5. Bioluminescence imaging of Luc-PANC-1 derived tumors (IP treatment). Bioluminescence imaging of Luc-PANC-1 derived tumors showing the bioluminescence photon flux over time for animals treated via IP injection with compound 19-S (19-S) (*n* = 4) or vehicle control (*n* = 4).

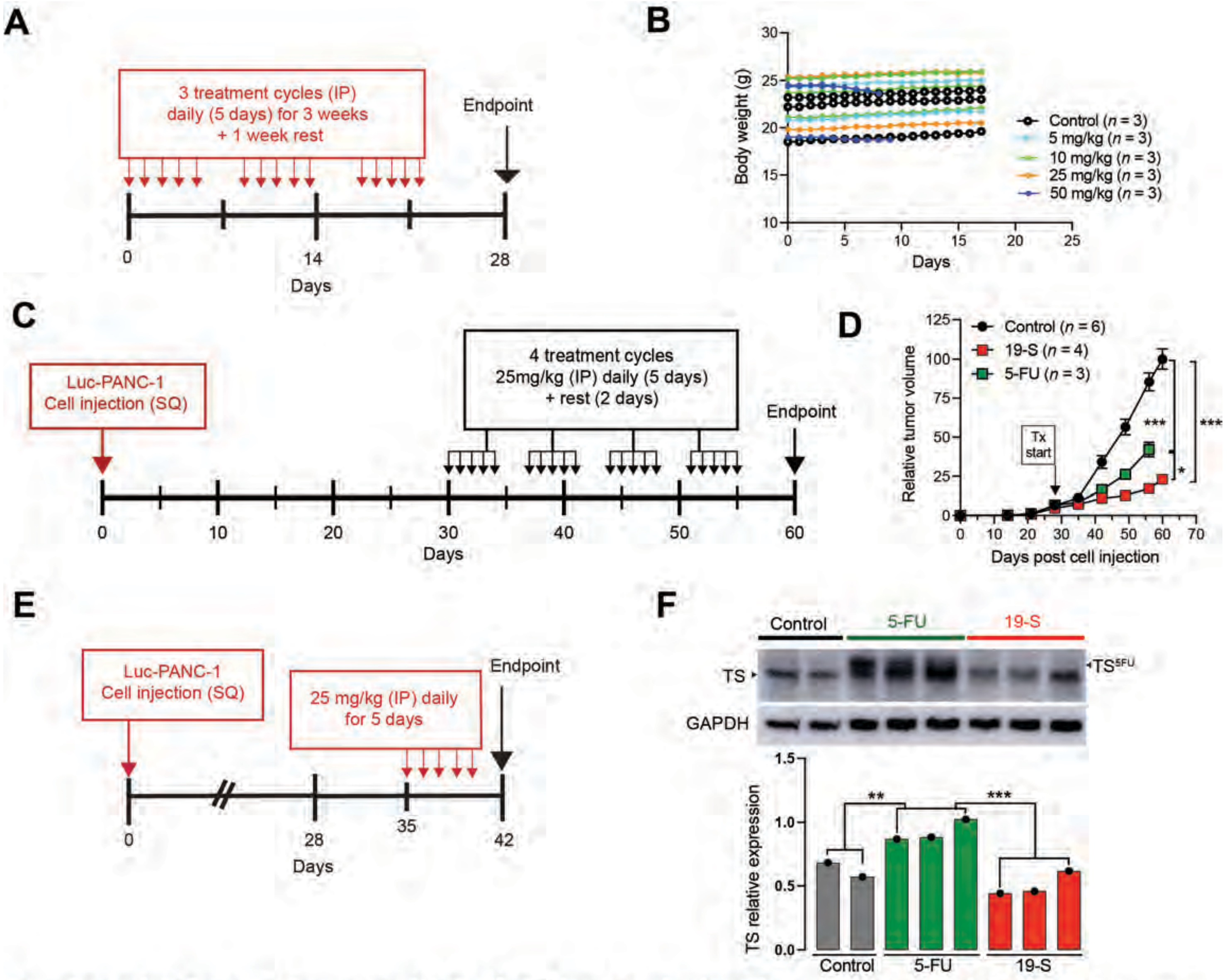

Supplemental Figure 6. **Compound 19-S (19-S) has a potent antitumoral effect and does not increase TYMS (TS) levels in Luc-PANC-1 derived tumors.** (**A**) Timeline indicating IP treatment schedule for 5-FU in NSG mice to determine maximum tolerated dose (MTD). 5-FU was administered IP 5 days a week for 3 weeks and animals rested (no treatment) for one extra week before experimental endpoint. (**B**) Effect of 5-FU [5 mg/kg (*n* = 3), 10 mg/kg (*n* = 3), 25 mg/kg (*n* = 3) and 50 mg/kg (*n* = 3)] on BW following the treatment schedule shown in A, compared to vehicle control (*n* = 3). Individual mice weight is represented. Out of *n* = 3 animals treated with 50 mg/kg, 2 were found dead after 8th dose and one animal after 9th dose. (**C**) Experimental timeline for the subcutaneous Luc-PANC-1 cell line derived tumor model treated with compound 19-S, 5-FU or vehicle control. Timeline indicates the subcutaneous injection of Luc-PANC-1 cells to generate tumors and compound 19-S (25 mg/kg, *n* = 4), 5-FU (25 mg/kg, *n* = 3) or vehicle control (*n* = 6) treatment cycles administered by IP injection. For each cycle animals were treated once a day for 5 continuous days then allowed 2 days rest when no treatments were administered. Tumor bearing mice received a total of 4 treatment cycles before endpoint. (**D**) Luc-PANC-1 relative tumor volumes for animals treated with compound 19-S, 5-FU or vehicle control as described in C. Compound 19-S (*n* = 4) and vehicle control (*n* = 4) treated animals from Figure 2D were compared to 5-FU (*n* = 3) and vehicle control treated mice (*n* = 2). Relative tumor volume was calculated for each group using combined controls (*n* = 6) as 100%. Data is expressed as mean ± SEM. Statistical analysis was performed using a 2-way ANOVA, Tukey's multiple comparison test, $^{*}P = 0.049$, $^{***}P < 0.001$. (**E**) Experimental timeline for the subcutaneous Luc-PANC-1 cell line derived xenograft tumor model. Tumors were generated in NSG mice and then were treated with compound 19-S (25 mg/kg, *n* = 3), 5-FU (25 mg/kg, *n* = 3) or vehicle control (*n* = 2) by IP injection once a day for 5 continuous days then allowed 2 days rest when no treatments were administered. (**F**) TS Immunoblot analysis and relative quantification from tumors generated as described in E. TS overexpression following 5-FU treatment (TS^{5FU}) and stable TS expression levels following compound 19-S treatment is shown. Significance was calculated by 2-way ANOVA Tukey's Multiple comparison test, $^{**}P = 0.030$, $^{***}P = 0.0005$.

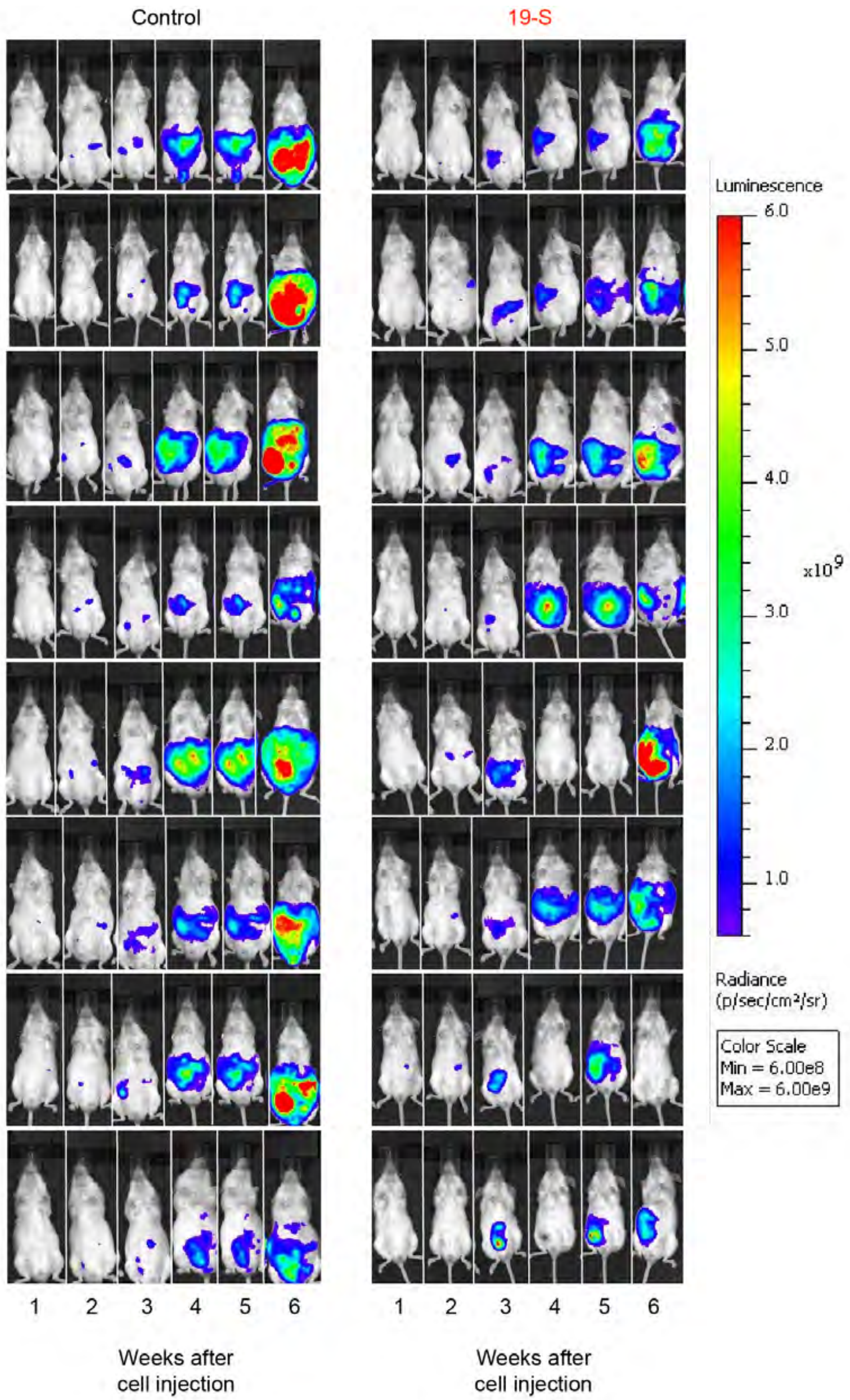

Supplemental Figure 7. **Bioluminescence imaging of Luc-CM derived tumors (IP treatment).** Bioluminescence imaging of Luc-CM tumor bearing NSG mice showing the bioluminescence photon flux from the abdominal region to quantify tumor burden over time for animals treated via IP injection with compound 19-S (19-S) (n = 8) or vehicle control (n = 8).

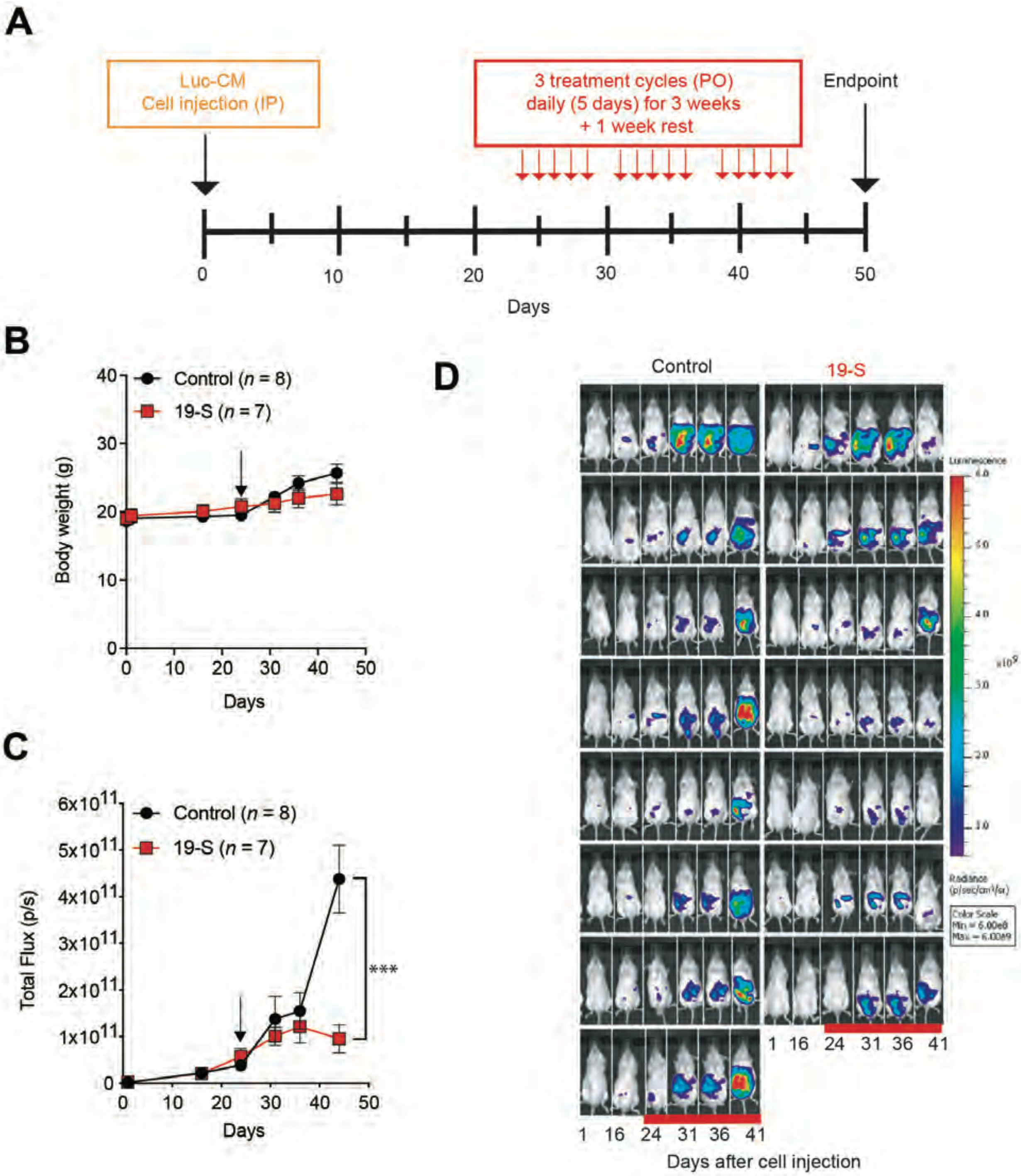

Supplemental Figure 8. Compound 19-S (19-S) inhibits tumor growth and progression at 25 mg/kg in a Luc-CM disseminated tumor model (PO treatment). (**A**) Timeline indicating IP injection of Luc-CM cells to generate disseminated tumor model and compound 19-S (25 mg/kg) or vehicle control treatment cycles administered PO. For each cycle animals were treated once a day for 5 consecutive days followed by 2 days rest (no treatment administered). After 3 treatment cycles, mice rested for one extra week without treatment before endpoint. (**B**) Effect of compound 19-S on BW compared to matched vehicle control (administered as indicated in A) to Luc-CM tumor bearing NSG mice. Data are expressed as mean ± SEM of n = 8 controls and n = 7 compound 19-S treated mice. Arrow indicates day when treatment started. (**C**) Bioluminescence quantification of total photon flux of abdominal region of Luc-CM tumor bearing NSG mice treated PO with compound 19-S (n = 7) or vehicle control (n = 8). Data are expressed as mean ± SEM. A two-way ANOVA was used to assess differences between control and treated mice over time, ***$P < 0.0001$. Arrow indicates day when treatment started. (**D**) Bioluminescence images of Luc-CM tumor bearing NSG mice to determine tumor burden over time in animals treated with compound 19-S compared to vehicle control mice. Red bar over days after cell injection indicates days of treatment.

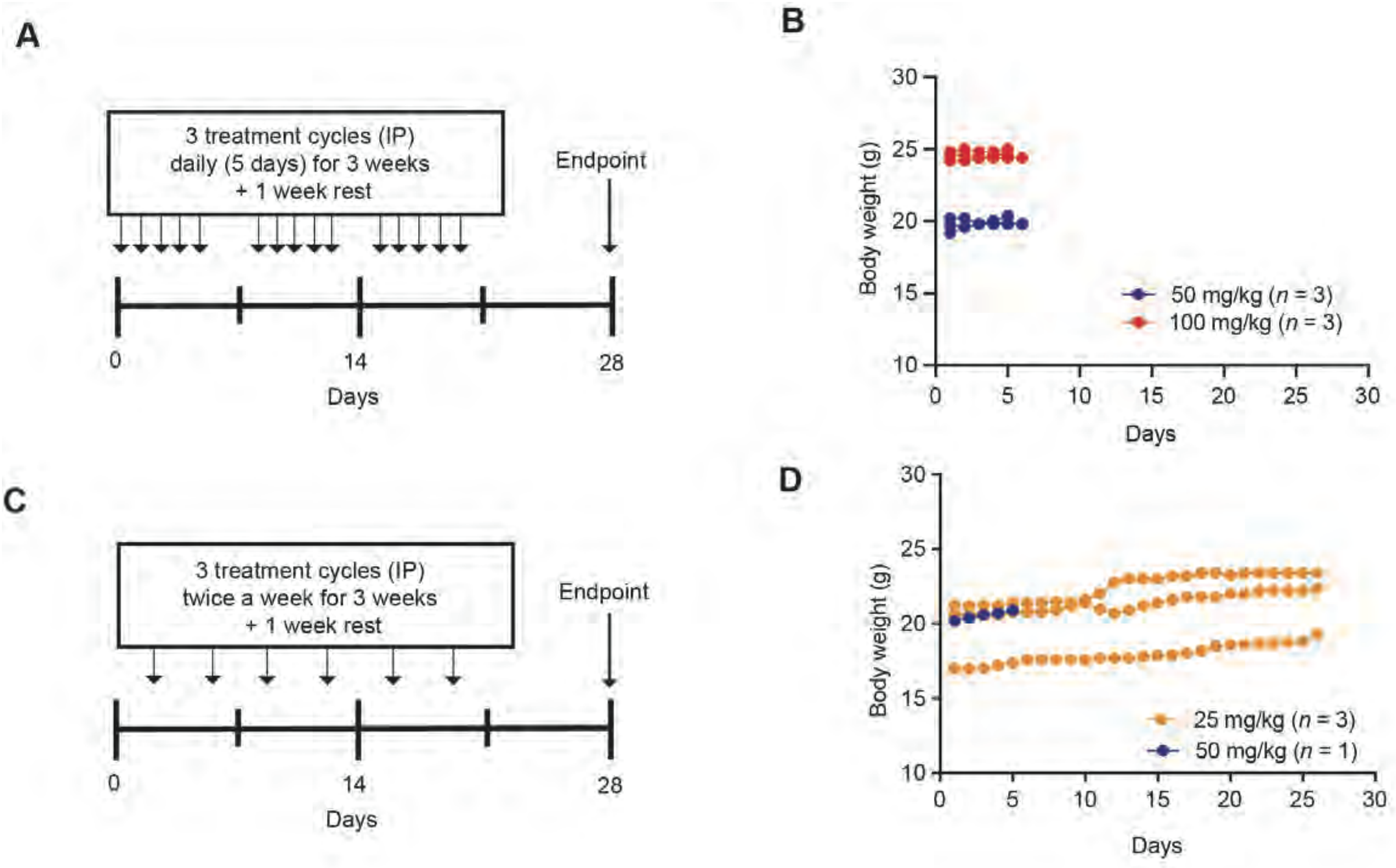

Supplemental Figure 9. **Compound 19-S (19-S) is not toxic at a dose of 25 mg/kg for IP treatment in FVB/129/sv mice.** (**A**) Timeline indicating IP treatment schedule for compound 19-S in FVB/129/sv (*Ink4a/Arf* $^{+/+}$) mice to determine maximum tolerated dose (MTD). Compound 19-S was administered IP 5 days a week for 3 weeks and animals rested for one extra week without treatment before endpoint. (**B**) Effect of compound 19-S [50 mg/kg (*n* = 3) and 100 mg/kg (*n* = 3)] on BW administered as shown in A. From the 100 mg/kg treated group, 2 mice out of 3 were found dead after 5th dose; 1 mouse, after 6th dose. From the 50 mg/kg group, one mouse out of 3 was found dead after the 5th dose and 2 mice were found dead after the 6th dose. (**C**) Timeline indicating IP treatment schedule for compound 19-S in FVB/129/sv (*Ink4a/Arf* $^{-/-}$) mice to determine MTD. Compound 19-S was administered IP twice a week for 3 weeks and animals rested for one extra week without treatment before endpoint. (**D**) Effect of compound 19-S [25 mg/kg (*n* = 3) and 50 mg/kg (*n* = 1) on BW, administered as shown in C. One mouse from the 50 mg/kg treated group was found dead after 5th dose, all 25 mg/kg mice (*n* = 3) reached endpoint.

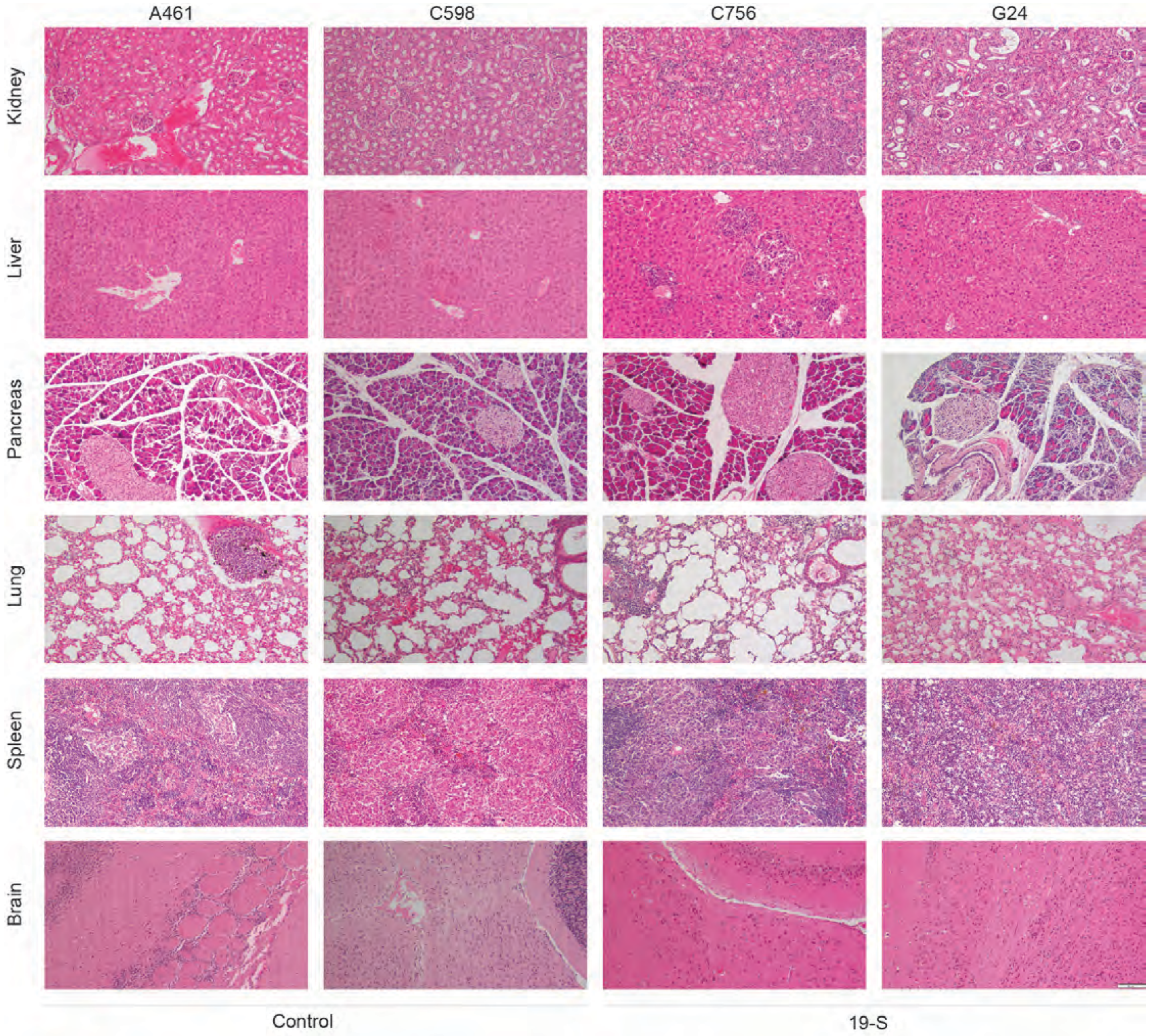

Supplementary Figure 10. **Compound 19-S (19-S) shows no evidence of drug injury at 10 mg/kg in *hTS/Ink4a/Arf* $^{-/-}$ mice.** Representative pictures of H&E stained FFPE sections of kidney, liver, pancreas, lung, spleen and brain from compound 19-S (C756 and G24) compared to vehicle control treated *hTS/Ink4a/Arf* $^{-/-}$ mice (A461 and C598). No evidence of drug tissue injury was found in any of the organs examined from 19-S treated mice compared to control mice. Scale bar represents 100 μm.

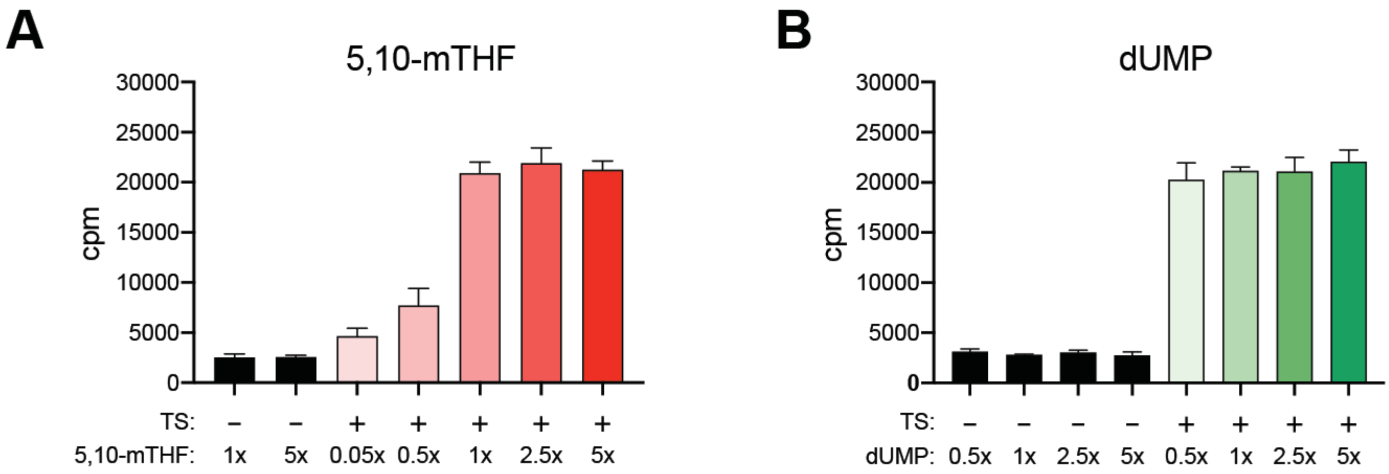

Supplemental Figure 11. **Increased dUMP and 5,10-mTHF concentrations do not impact conversion of dUMP in a tritium based TYMS (TS) catalytic assay.** Tritium assay for TS activity showing the actual scintillation counts recorded from the indicated reaction conditions to demonstrate how increased substrate concentrations: 5, 10-mTHF (A) and dUMP (B) do not impact the total conversion of tritiated dUMP since the standard 1x substrate concentrations already provide saturating substrate conditions.

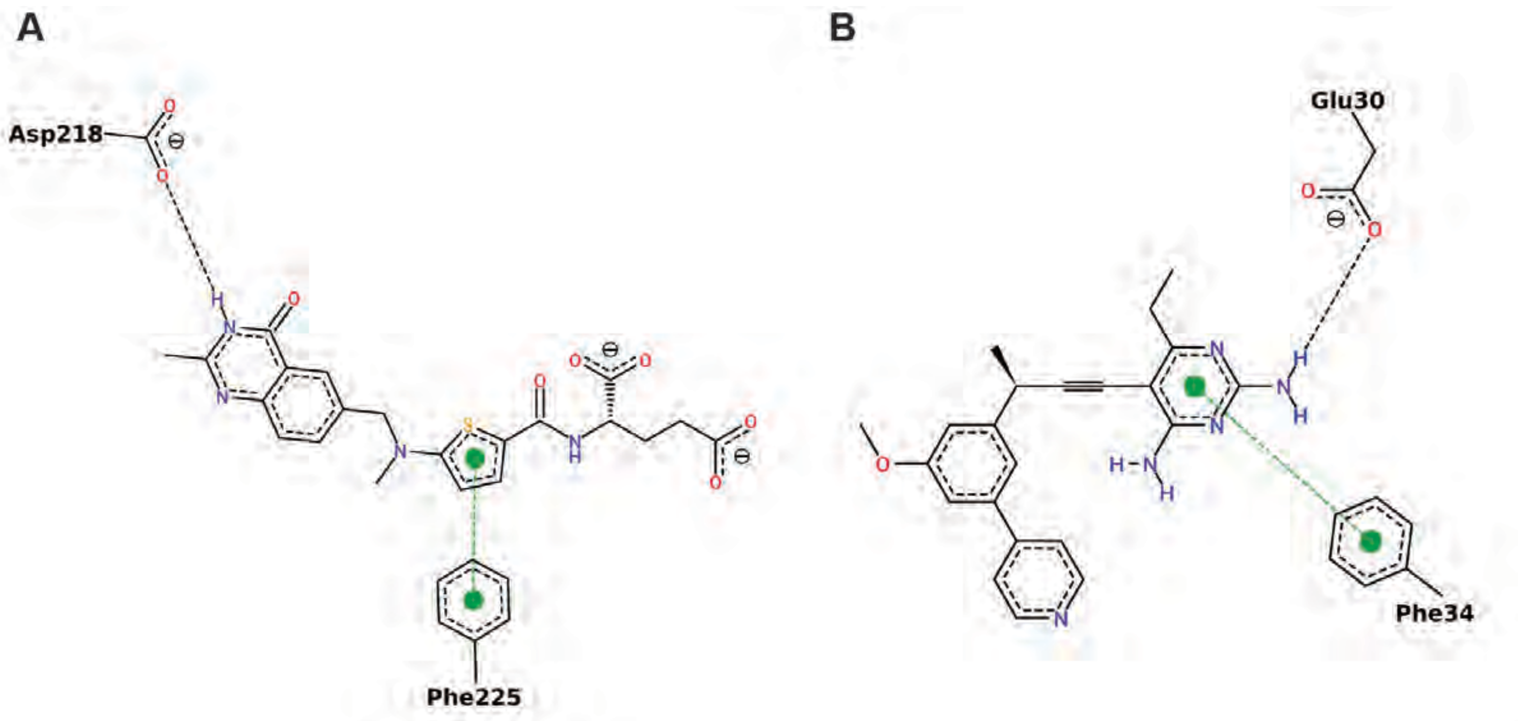

Supplemental Figure 12. Pharmacophoric interaction used as a restraint for biased docking simulations. Panel A and B show interactions used as pharmacophoric restraints for TYMS (PDB ID: 1HVY) and DHFR (PDB ID: 4KAK), respectively. Polar and aromatic restraints are presented as black and green dotted dashed lines, respectively.

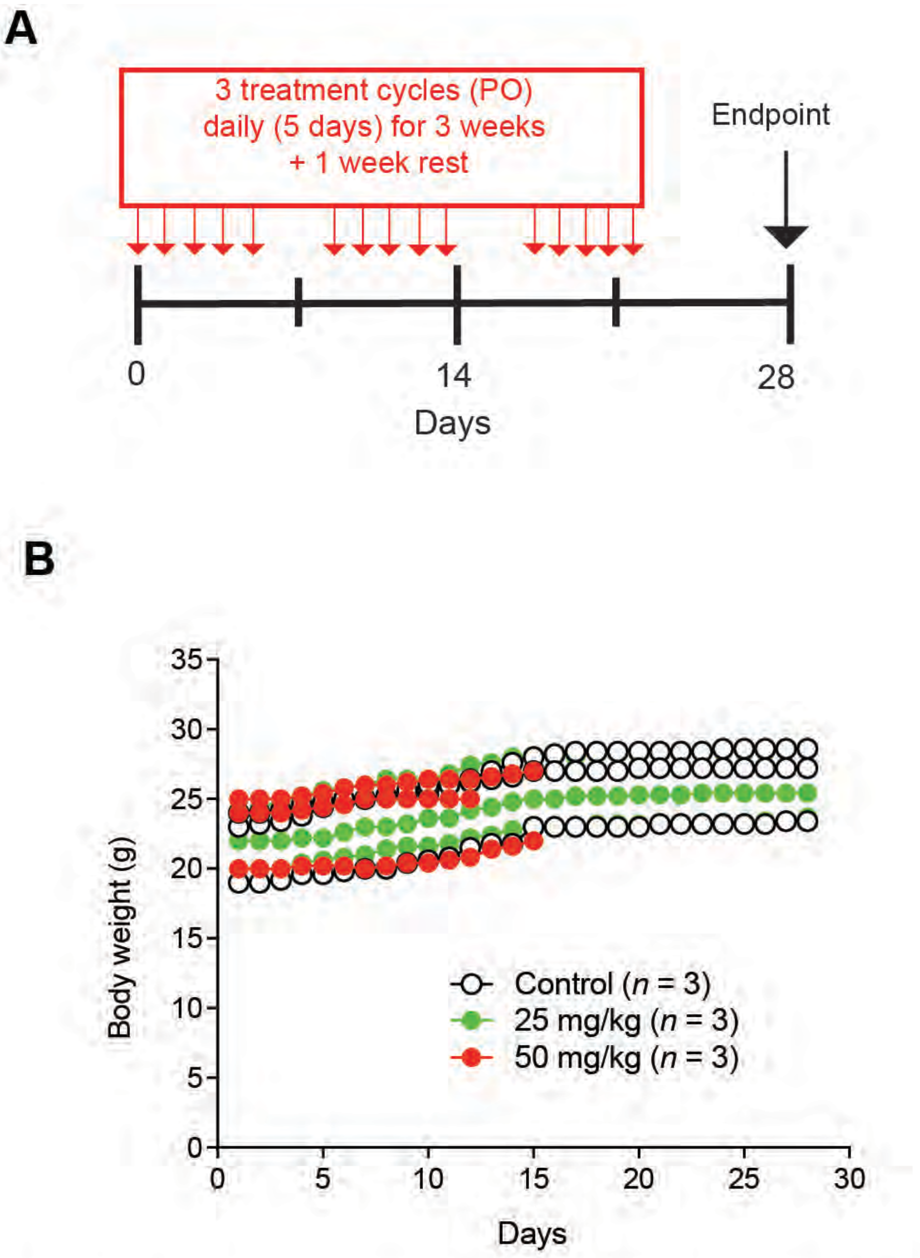

Supplemental Figure 13. Compound 19-S7 is not toxic at 25 mg/kg for PO treatment in NSG mice. (**A**) Timeline indicating PO administration of compound 19-S7 in NSG mice to determine MTD. 25 mg/kg, 50 mg/kg or vehicle control treatment was administered PO to 6-8 weeks old NSG mice. Schedule was once a day for five continuous days, then allowed 2 days to rest when no treatment was administered. After 3 treatment cycles, animals rest for 1 week with no treatment before endpoint. (**B**) Effect of compound 19-S7 [25 mg/kg (*n* = 3) and 50 mg/kg (*n* = 3)] compared to vehicle control (*n* = 3) on NSG BW. Individual mice BW is represented. 50 mg/kg treated mice (*n* = 3) were found dead after 12th (*n* = 1) and 15th (*n* = 2) dose, respectively. Animals treated with 25 mg/kg (*n* = 3) reached endpoint.

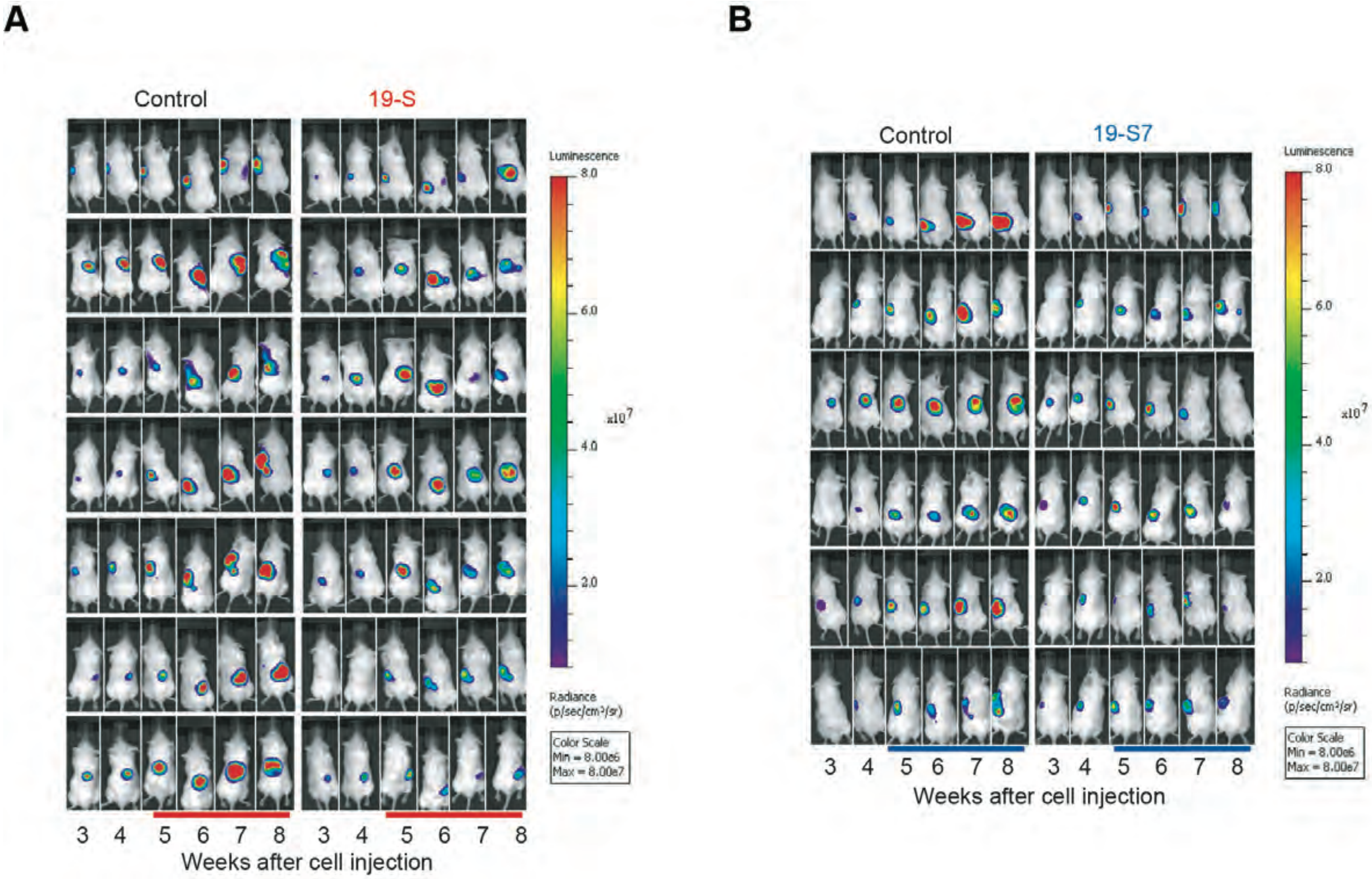

Supplemental Figure 14. Bioluminescence imaging of Luc-PANC-1 derived tumors (PO treatment). (**A**) Bioluminescence imaging of Luc-PANC-1 derived tumors over time for animals treated with 19-S (19-S) (*n* = 7) or vehicle control (*n* = 7). (**B**) Bioluminescence imaging of Luc-PANC-1 derived tumors over time for animals treated with 19-S7 (*n* = 6) or vehicle control (*n* = 6). Red/blue bars over weeks after cell injection label indicates treatment phase.

Type of bias	TYMS	DHFR	Modified maps
Acceptor	Asp218	Glu30	OA, NA
Aromatic	Phe225	Phe34	AC

Supplemental Table 1. Pharmacophoric interaction used as a restraint for biased docking simulations.

**Mutation**	ΔΔG **(Kcal/mol)**
W109A	+1.79
D218A	+2.31
F225A	+1.97
V79A	-0.48
L221A	+0.72
I108A	+0.08

Supplemental Table 2. Effect of substitution by Alanine of TS ligand binding site amino acids in 19-S compound affinity. Details of experiment are explained in Supplemental Methods.

**Mutation**	ΔΔG **(Kcal/mol)**
E30A	+2.52
F34A	+1.80
S59A	+2.09
L67A	+0.82
I60A	+0.33
F31A	+0.15
L22A	+0.52
V8A	+0.37

Supplemental Table 3. Effect of substitution by Alanine of DHFR ligand binding site amino acids in 19-S compound affinity. Details of experiment are explained in Supplemental Methods.

Chemical Synthesis, Characterization Data, and NMR Spectra

A) Chemical Synthesis and Characterization Data.

All reagents for chemical synthesis were purchased at ≥ 95% purity from commercial sources and used without further purification. Analytical thin layer chromatography (TLC) was performed using 250 μm Silica Gel 60 F254 pre-coated plates (EMD Chemicals Inc.) and used to monitor all reactions. Flash column chromatography was performed using 230-400 Mesh 60Å Silica Gel from Sorbent Technologies. Melting points were obtained, uncorrected, using a Mel-Temp capillary melting point apparatus from Laboratory Services, Inc.

NMR experiments were recorded using broadband probes on a Varian Mercury-Plus-400 spectrometer via VNMR-J software (400 MHz for ^{1}H and 101 MHz for ^{13}C), Varian Mercury-Plus-500 spectrometer via VNMR-J software (500 MHz for ^{1}H and 126 MHz for ^{13}C), and Bruker Avance III (500 MHz for ^{1}H; 126 MHz for ^{13}C). All spectra are presented using MestReNova 11.0 (Mnova) software and are displayed without the use of the signal suppression function. Spectra were obtained at room temperature in the following solvents (reference peaks for ^{1}H and ^{13}C NMRs are included): $CDCl_3$ (^{1}H NMR, 7.26 ppm; ^{13}C NMR, 77.23 ppm) and DMSO-d_6 (^{1}H NMR, 2.50 ppm; ^{13}C NMR, 39.52 ppm). Chemical shift values (δ) are reported in parts per million (ppm) for all ^{1}H NMR and ^{13}C NMR spectra. ^{1}H NMR multiplicities are reported as: s = singlet, br. s = broad singlet, d = doublet, t = triplet, q = quartet, m = multiplet. HSQC was used to identify a few challenging ^{13}C signals and those spectra are reported in the supporting information. High-Resolution Mass Spectrometry (HRMS) were obtained for all new compounds from the Chemistry Department at the University of Florida.

Synthesis Overview

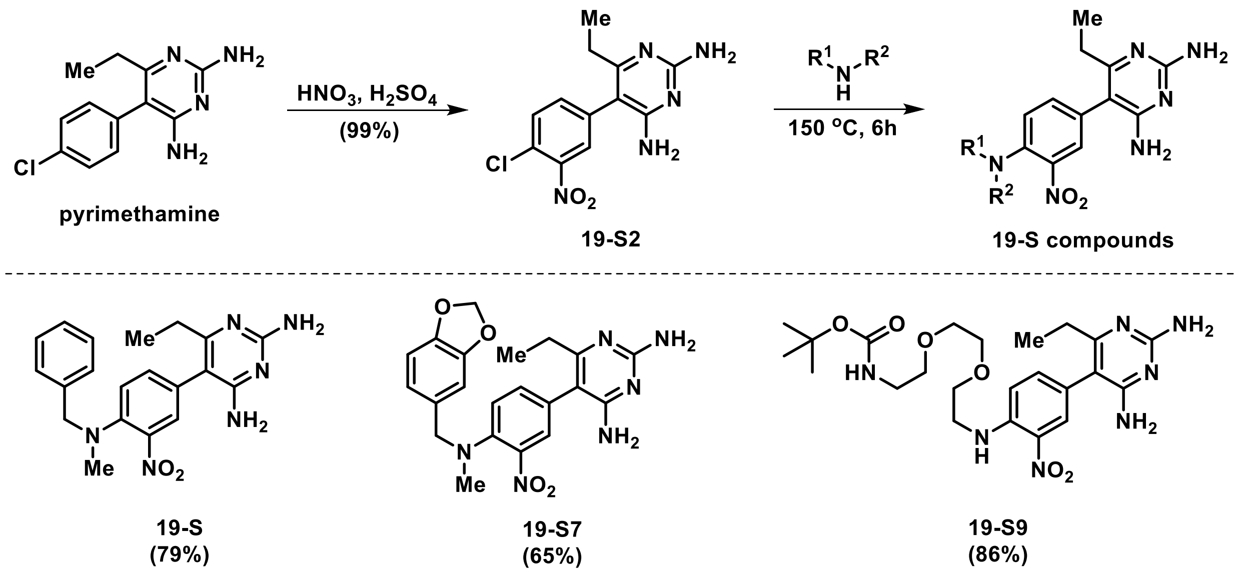

Compound **19-S** (19-S) and derivatives were synthesized through a known two-step route (14). First, commercially available pyrimethamine was selectively *ortho*-nitrated using nitric acid in sulfuric acid (99% yield). The nitro precursor was subjected to a nucleophilic aromatic substitution reaction with various amines to produce Compound S and derivatives in a rapid and high yielding synthetic sequence (12 examples, average yield = 58%). Additionally, both the nitration reaction as well as several analogues were able to scale to several hundreds of milligrams with negligible loss in yield, which provided more than enough material for substantial *in vivo* testing in mice tumor models (e.g., second step scales for select analogues of interest include: 391 mg **19-S**, 461 mg **19-S7**).

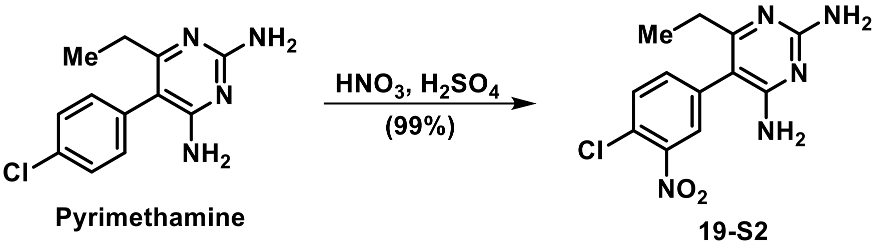

Procedure for the synthesis of 19-S2 (19-S2): A round-bottom flask was added concentrated sulfuric acid (2.0 mL) and cooled to 0 °C. Concentrated nitric acid was added dropwise and allowed to stir for 5 minutes. Pyrimethamine (500 mg, 2.01 mmol) was added in several portions and the reaction was stirred at 0 °C for 15 minutes, then allowed to warm to room temperature, then heated to 50 °C for 1 hour. The resulting yellow solution was cooled to room temperature, then slowly added to a flask containing ice:ammonium hydroxide:water which produced a yellow precipitate. The precipitate was

filtered, washed with additional water, and dried to afford pure 19-S**2** (589 mg, 99%) as a yellow solid. Note: **19-S2** is a known compound (CAS number: 21813-35-4).

^{1}H NMR: (400 MHz, d_6-DMSO) δ 7.87 (d, *J* = 2.1 Hz, 1H), 7.78 (d, *J* = 8.3 Hz, 1H), 7.51 (dd, *J* = 8.3, 2.1 Hz, 1H), 5.99 (br. s, 2H), 5.86 (br. s, 2H), 2.12 (q, *J* = 7.5 Hz, 2H), 0.98 (t, *J* = 7.5 Hz, 3H).

^{13}C NMR: (101 MHz, d_6-DMSO) δ 166.4, 162.3, 162.0, 148.0, 137.0, 136.6, 132.0, 127.9, 123.7, 103.6, 27.4, 12.9.

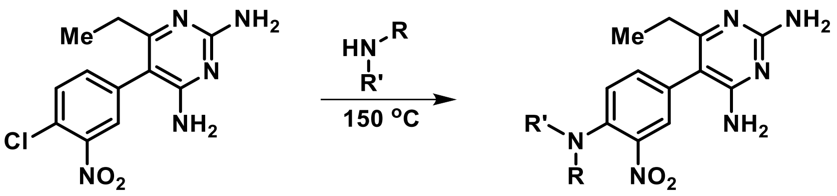

19-S2

General procedure for the synthesis of 19-S compounds: 19-S2 (293 mg, 1.00 mmol) was added to a screw-top vessel, purged with argon and added benzylmethylamine (1 mL, 7.75 mmol). The vessel was sealed and heated to 150 °C for 6 hours. The resulting dark-red solution was cooled to room temperature and diethyl ether was added (35 mL). The precipitate was collected, washed with additional ether and dried. The crude precipitate was purified via recrystallization from 2-ethoxyethanol: water 3.5:1 to afford compound **19-S** (300 mg, 79%) as a red, crystalline solid.

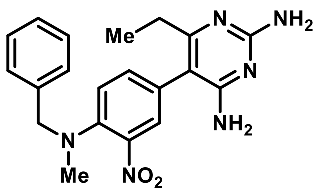

19-S

Yield: 79%; 300 mg of **19-S** isolated as a red, crystalline solid. Note: **19-S** is a known compound (CAS number: 118344-71-1); however, no published spectra were found for comparison.

^{1}H NMR: (400 MHz, d_6-DMSO) δ 7.55 (s, 1H), 7.39 – 7.33 (m, 2H), 7.33 – 7.24 (m, 5H), 5.90 (br. s, 2H), 5.72 (br. s, 2H), 4.42 (br. s, 2H), 2.72 (s, 3H), 2.12 (q, *J* = 7.5 Hz, 2H), 0.97 (t, *J* = 7.5 Hz, 3H).

^{13}C NMR: (101 MHz, d_6-DMSO) δ 166.7, 162.3, 162.1, 144.3, 140.1, 137.4, 135.9, 128.5, 127.7, 127.5, 127.2, 126.7, 120.6, 104.4, 57.8, 40.3, 27.4, 13.1.

MP: 208 – 210 °C, lit: 210 – 211 °C.

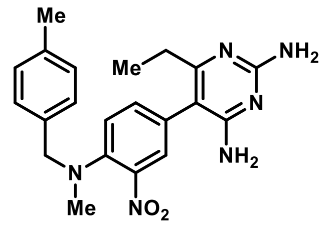

19-S1

Yield: 74%; 580 mg of **19-S1** isolated as an orange, crystalline solid.

^{1}H NMR: (400 MHz, d_6-DMSO) δ 7.54 (d, *J* = 1.7 Hz, 1H), 7.26 (d, *J* = 1.9 Hz, 2H), 7.22 – 7.03 (m, 4H), 5.89 (br s, 2H), 5.70 (br s, 2H), 4.36 (br s, 2H), 2.70 (s, 3H), 2.28 (s, 3H), 2.12 (q, *J* = 7.5 Hz, 2H), 0.97 (t, *J* = 7.5 Hz, 3H).

^{13}C NMR: (101 MHz, d_6-DMSO) δ 166.9, 162.3, 162.1, 144.3, 140.1, 136.3, 135.9, 134.2, 129.0, 127.7, 127.6, 126.6, 120.6, 104.4, 57.5, 40.2, 27.5, 20.7, 13.1.

HRMS (ESI): calc. for $C_{21}H_{25}N_6O_2$ $[M + H]^+$: 393.2034, found: 393.2046.

MP: 232 – 233 °C.

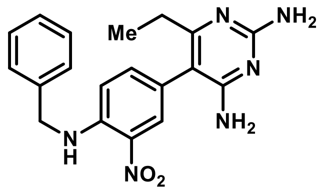

19-S3

Yield: 62%; 78.3 mg of **19-S3** (**19-S3**) isolated as a red, crystalline solid. Note: **19-S3** is a known compound (CAS number: 118344-70-0); however, no published spectra were found for comparison.

^{1}H NMR: (400 MHz, d_6-DMSO) δ 8.72 (t, *J* = 6.1 Hz, 1H), 7.82 (d, *J* = 2.1 Hz, 1H), 7.45 – 7.40 (m, 2H), 7.40 – 7.33 (m, 2H), 7.31 – 7.23 (m, 2H), 6.99 (d, *J* = 8.9 Hz, 1H), 5.84 (br. s, 2H), 5.66 (br. s, 2H), 4.65 (d, *J* = 6.0 Hz, 2H), 2.10 (q, *J* = 7.5 Hz, 2H), 0.95 (t, *J* = 7.5 Hz, 3H).

^{13}C NMR: (101 MHz, d_6-DMSO) δ 167.0, 162.5, 162.1, 144.2, 139.3, 138.6, 131.4, 128.6, 127.8, 127.1 (2), 122.9, 115.4, 104.4, 45.9, 27.5, 13.1.

HRMS (ESI): calc. for $C_{19}H_{21}N_6O_2$ $[M + H]^+$: 365.1721, found: 365.1719.

MP: >250 °C, lit: 253 – 255 °C.

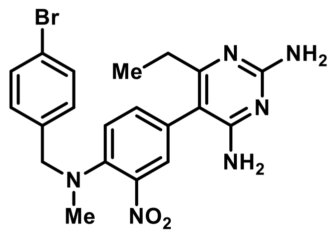

19-S4

Yield: 78%; 204 mg of **19-S4** (**19-S4**) isolated as a red-orange solid.

^{1}H NMR: (400 MHz, d_6-DMSO) δ 7.60 – 7.51 (m, 3H), 7.32 – 7.22 (m, 4H), 5.88 (br. s, 2H), 5.69 (br. s, 2H), 4.38 (s, 2H), 2.71 (s, 3H), 2.11 (q, *J* = 7.5 Hz, 2H), 0.97 (t, *J* = 7.5 Hz, 3H).

^{13}C NMR: (101 MHz, d_6-DMSO) δ 166.9, 162.3, 162.1, 144.1, 140.4, 136.9, 136.0, 131.3, 129.8, 127.7, 127.2, 120.8, 120.2, 104.3, 57.2, 40.4, 27.5, 13.1.

HRMS (ESI): calc. for $C_{20}H_{22}BrN_6O_2$ $[M + H]^+$: 457.0982, found: 457.0996.

MP: 219 – 221 °C.

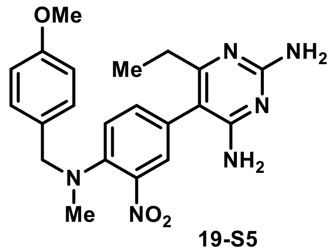

19-S5

Yield: 48%; 107 mg of **19-S5** (**19-S5**) isolated as an orange solid. Note: **19-S5** has an assigned CAS number (118344-80-2), but no published spectra were found for comparison.

^{1}H NMR: (400 MHz, d_6-DMSO) δ 7.54 (s, 1H), 7.21 (d, J = 8.3 Hz, 2H), 7.21 (d, J = 8.5 Hz, 2H), 6.91 (d, J = 8.3 Hz, 2H), 5.88 (br. s, 2H), 5.69 (br. s, 2H), 4.32 (s, 2H), 3.74 (s, 3H), 2.68 (s, 3H), 2.12 (q, J = 7.5 Hz, 2H), 0.97 (t, J = 7.5 Hz, 3H).

^{13}C NMR: (101 MHz, d_6-DMSO) δ 166.9, 162.3, 162.1, 158.5, 144.3, 140.3, 135.9, 129.0, 128.9, 127.6, 126.8, 120.7, 113.8, 104.4, 57.3, 55.0, 40.0, 27.5, 13.1.

HRMS (ESI): calc. for $C_{21}H_{25}N_6O_3$ $[M + H]^+$: 409.1983, found: 409.1999.

MP: 195 – 197 °C, lit: 201 – 203 °C.

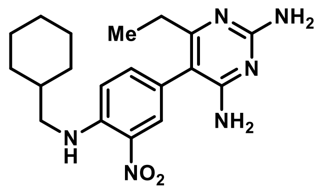

19-S6

Yield: 70%; 136 mg of **19-S6** (**19-S6**) isolated as an orange crystalline solid.

^{1}H NMR: (400 MHz, d_6-DMSO) δ 8.26 (t, J = 5.2 Hz, 1H), 7.79 (d, J = 1.6 Hz, 1H), 7.31 (dd, J = 8.5, 2.1 Hz, 1H), 7.11 (d, J = 8.9 Hz, 1H), 5.84 (br. s, 2H), 5.68 (br. s, 2H), 3.24 (t, J = 6.3 Hz, 2H), 2.13 (q, J = 7.5 Hz, 2H), 1.85 – 1.58 (m, 6H), 1.31 – 1.10 (m, 3H), 1.10 – 1.01 (m, 2H), 0.97 (t, J = 7.5 Hz, 3H).

^{13}C NMR: (101 MHz, d_6-DMSO) δ 167.0, 162.5, 162.1, 144.7, 139.5, 130.9, 127.7, 122.5, 115.2, 104.5, 48.5, 36.7, 30.4, 27.5, 26.0, 25.4, 13.1.

HRMS (ESI): calc. for $C_{19}H_{27}N_6O_2$ $[M + H]^+$: 371.2190, found: 271.2196.

MP: >250 °C.

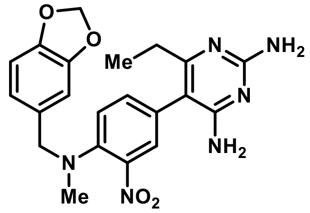

19-S7

Yield: 65%; 148 mg of **19-S7** (**19-S7**) isolated as an orange solid.

^{1}H NMR: (400 MHz, d_6-DMSO) δ 7.54 (t, J = 1.2 Hz, 1H), 7.28 (d, J = 1.2 Hz, 2H), 6.88 (d, J = 7.9 Hz, 1H), 6.83 (d, J = 1.6 Hz, 1H), 6.77 (dd, J = 7.9, 1.7 Hz, 1H), 6.00 (s, 2H), 5.88 (s, 2H), 5.70 (br. s, 2H), 4.29 (br. s, 2H), 2.69 (s, 3H), 2.12 (q, J = 7.5 Hz, 2H), 0.97 (t, J = 7.5 Hz, 3H).

^{13}C NMR: (101 MHz, d_6-DMSO) δ 166.8, 162.3, 162.1, 147.4, 146.4, 144.2, 140.4, 135.9, 131.1, 127.6, 126.9, 120.9, 120.8, 108.1, 107.9, 104.4, 100.9, 57.6, 40.2 (buried under d_6-DMSO), 27.5, 13.0.

HRMS (ESI): calc. for $C_{21}H_{23}N_6O_4$ $[M + H]^+$: 423.1775, found: 423.1783.

MP: 194 – 196 °C.

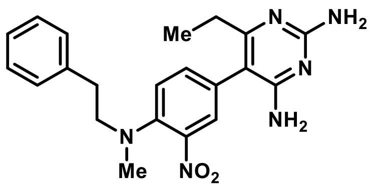

19-S8

Yield: 22%; 45.5 mg of **19-S8** (**19-S8**) isolated as an orange solid.

^{1}H NMR: (400 MHz, d_6-DMSO) δ 7.47 (s, 1H), 7.29 – 7.14 (m, 7H), 5.87 (br. s, 2H), 5.64 (br. s, 2H), 3.37 (m, 2H), 2.92 – 2.82 (m, 5H), 2.11 (q, J = 7.5 Hz, 2H), 0.97 (t, J = 7.5 Hz, 3H).

^{13}C NMR: (101 MHz, d_6-DMSO) δ 166.9, 162.3, 162.1, 143.8, 139.8, 139.1, 135.8, 128.7, 128.3, 127.7, 126.2, 126.0, 120.1, 104.4, 55.9, 39.7 (buried under d_6-DMSO), 32.9, 27.4, 13.1.

HRMS (ESI): calc. for $C_{21}H_{25}N_6O_2$ $[M + H]^+$: 393.2034, found: 393.2022.

MP: 184 – 186 °C.

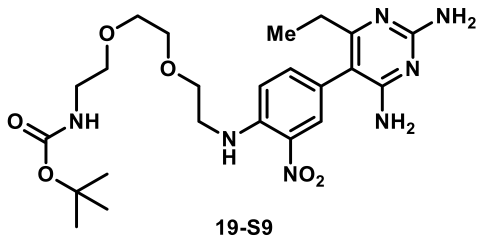

19-S9

Yield: 86%; 447 mg of **19-S9** (**19-S9**) isolated as a red-orange solid. Note: **19-S9** was purified via column chromatography using a gradient of 99:1 hexanes:triethylamine to 98:1:1 ethyl acetate:methanol:triethylamine.

^{1}H NMR: (400 MHz, $CDCl_3$) δ 8.19 (t, J = 5.0 Hz, 1H), 7.96 (d, J = 2.1 Hz, 1H), 7.24 (dd, J = 8.8, 2.1 Hz, 1H), 6.88 (d, J = 8.9 Hz, 1H), 5.55 (t, J = 5.9 Hz, 1H), 5.30 (s, 2H), 5.17 – 4.94 (m, 2H), 3.73 (t, J = 5.3 Hz, 2H), 3.67 – 3.56 (m, 4H), 3.54 – 3.39 (m, 4H), 3.30 – 3.19 (m, 2H), 2.18 (q, J = 7.6 Hz, 2H), 1.36 (s, 9H), 0.97 (t, J = 7.6 Hz, 3H).

^{13}C NMR: (101 MHz, $CDCl_3$) δ 168.8, 162.7, 161.9, 156.1, 144.8, 138.9, 132.1, 128.5, 122.2, 115.0, 106.0, 79.2, 70.6, 70.5, 70.3, 68.9, 42.9, 40.4, 28.5, 28.1, 13.4.

HRMS (ESI): calc. for $C_{23}H_{36}N_7O_6$ $[M + H]^+$: 506.2722, found: 506.2742.

MP: 87 – 89 °C.

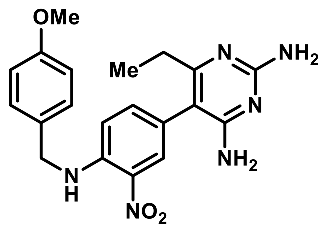

19-S11

Yield: 27%; 57.8 mg of **19-S11** (**19-S11**) isolated as a red, crystalline solid. Note: **19-S11** is a known compound (CAS number: 118344-94-8); however, no published spectra were found for comparison.

^{1}H NMR: (400 MHz, *d*$_6$-DMSO) δ 8.63 (t, *J* = 5.7 Hz, 1H), 7.81 (s, 1H), 7.36 (d, *J* = 8.2 Hz, 2H), 7.27 (d, *J* = 8.8 Hz, 1H), 7.03 (d, *J* = 8.8 Hz, 1H), 6.93 (d, *J* = 8.2 Hz, 2H), 5.84 (br. s, 2H), 5.65 (br. s, 2H), 4.56 (d, *J* = 5.8 Hz, 2H), 3.73 (s, 3H), 2.10 (q, *J* = 7.5 Hz, 2H), 0.95 (t, *J* = 7.5 Hz, 3H).

^{13}C NMR: (101 MHz, *d*$_6$-DMSO) δ 166.9, 162.5, 162.0, 158.5, 144.2, 139.3, 131.3, 130.3, 128.5, 127.8, 122.8, 115.4, 114.0, 104.4, 55.1, 45.4, 27.4, 13.1.

HRMS (ESI): calc. for $C_{20}H_{23}N_6O_3$ $[M + H]^+$: 395.1826, found: 395.1826.

MP: 242 – 244 °C, lit: 241 – 242 °C.

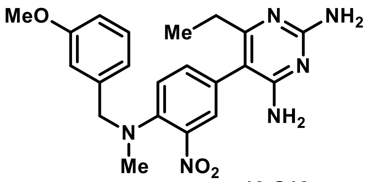

19-S12

Yield: 55%; 121 mg of **19-S12** (**19-S12**) isolated as a yellow-orange solid.

^{1}H NMR: (400 MHz, *d*$_6$-DMSO) δ 7.54 (s, 1H), 7.30 – 7.22 (m, 3H), 6.90 – 6.80 (m, 3H), 5.87 (br. s, 2H), 5.68 (br. s, 2H), 4.40 (s, 2H), 3.73 (s, 3H), 2.72 (s, 3H), 2.11 (q, *J* = 7.5 Hz, 2H), 0.97 (t, *J* = 7.5 Hz, 3H).

^{13}C NMR: (101 MHz, *d*$_6$-DMSO) δ 166.9, 162.3, 162.1, 159.4, 144.2, 140.2, 139.1, 135.9, 129.5, 127.7, 126.8, 120.6, 119.6, 113.0, 112.6, 104.3, 57.6, 54.9, 40.5, 27.5, 13.0.

HRMS (ESI): calc. for $C_{21}H_{25}N_6O_3$ $[M + H]^+$: 409.1983, found: 409.1991.

MP: 163 – 165 °C.

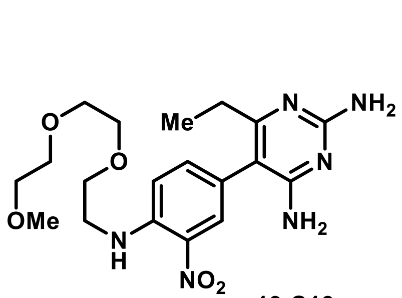

19-S13

Yield: 29%; 61.2 mg of **19-S13** (**19-S3**) isolated as an orange solid. Note: **19-S13** was purified via column chromatography using a gradient of 99:1 hexanes:triethylamine to 98:1:1 ethyl acetate:methanol:triethylamine.

^{1}H NMR: (400 MHz, $CDCl_3$) δ 8.27 (t, *J* = 5.2 Hz, 1H), 8.04 (d, *J* = 2.1 Hz, 1H), 7.28 (dd, *J* = 8.8, 2.1 Hz, 1H), 6.94 (d, *J* = 8.8 Hz, 1H), 5.04 (br. s, 2H), 4.76 (br. s, 2H), 3.79

(t, *J* = 5.3 Hz, 2H), 3.73 – 3.60 (m, 6H), 3.56 – 3.48 (m, 4H), 3.35 (s, 3H), 2.25 (q, *J* = 7.6 Hz, 2H), 1.04 (t, *J* = 7.6 Hz, 3H).

^{13}C NMR: (101 MHz, $CDCl_3$) δ 168.9, 162.7, 161.7, 145.0, 138.8, 132.4, 128.7, 121.9, 115.1, 106.4, 72.1, 70.9, 70.8, 70.8, 69.2, 59.2, 43.0, 28.2, 13.5.

HRMS (ESI): calc. for $C_{19}H_{28}N_6O_5Na$ $[M + Na]^+$: 443.2013, found: 443.2024.

MP: 131 – 133 °C.

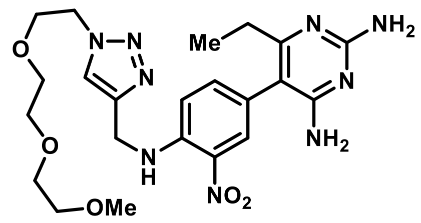

19-S14

Yield: 61%; 41.5 mg of **19-S14** (**19-S4**) isolated as an orange-red, amorphous solid. Note: **19-S14** was purified via silica gel, column chromatography using a gradient of 99:1 ethyl acetate:triethylamine to 97:2:1 ethyl acetate:methanol:triethylamine. After the first column, an unidentified impurity coeluted with the desired product. A second purification was performed via column chromatography, using alumina neutral act I and a gradient of 100% dichloromethane to 2% methanol:dichloromethane to afford pure product.

^{1}H NMR: (500 MHz, $CDCl_3$) δ 8.46 (t, *J* = 5.8 Hz, 1H), 8.09 (s, 1H), 7.81 (s, 1H), 7.32 (d, *J* = 8.7 Hz, 1H), 7.16 (d, *J* = 8.7 Hz, 1H), 4.78 (br. s, 2H), 4.70 (d, *J* = 5.6 Hz, 2H), 4.56 (t, *J* = 5.0 Hz, 2H), 4.50 (br. s, 2H), 3.87 (t, *J* = 5.0 Hz, 2H), 3.65 – 3.57 (m, 6H), 3.57 – 3.51 (m, 2H), 3.36 (s, 3H), 2.29 (q, *J* = 7.6 Hz, 2H), 1.08 (t, *J* = 7.6 Hz, 3H).

^{13}C NMR: (126 MHz, $CDCl_3$) δ 168.2, 162.8, 161.4, 144.5, 144.3, 138.9, 132.6, 128.6, 123.5, 122.3, 115.5, 106.3, 72.0, 70.6 (3), 69.5, 59.1, 50.6, 39.1, 28.0, 13.5.

HRMS (ESI): calc. for $C_{22}H_{32}N_9O_5$ $[M + H]^+$: 502.2521, found: 502.2537.

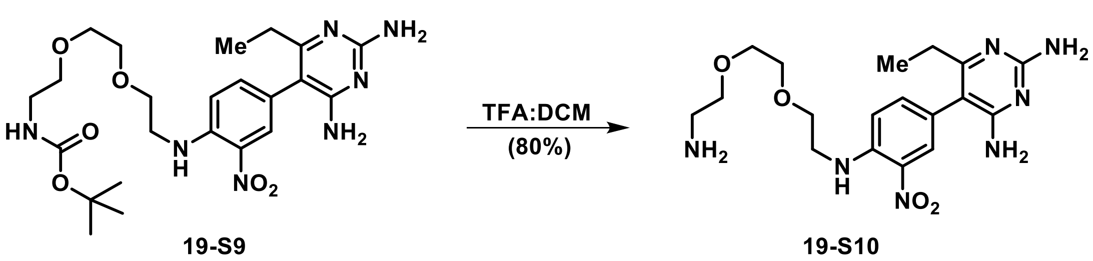

19-S9 **19-S10**

Procedure for the synthesis of 19-S10 (**19-S10**)**:** Compound **19-S9** (179 mg, 0.35 mmol) was added to a flame-dry round-bottom flask and dissolved in dichloromethane (1.5 mL). The solution was cooled

to 0 °C and added trifluoroacetic acid (1 mL) dropwise. The reaction was stirred at 0 °C for 0.5 hours then warmed to room temperature and concentrated *in vacuo*. The residue was dissolved in dichloromethane (30 mL) and extracted with saturated sodium bicarbonate solution (4 x 150 mL). The organics were pooled, dried with sodium sulfate, filtered and concentrated to afford pure compound **19-S10** (115 mg, 80%) as a red-orange solid.

^{1}H NMR: (400 MHz, d_6-DMSO) δ 8.26 (t, *J* = 5.3 Hz, 1H), 7.80 (d, *J* = 2.1 Hz, 1H), 7.33 (dd, *J* = 8.8, 2.2 Hz, 1H), 7.15 (d, *J* = 8.8 Hz, 1H), 5.85 (s, 2H), 5.68 (s, 2H), 3.71 (t, *J* = 5.4 Hz, 2H), 3.63 – 3.58 (m, 2H), 3.58 – 3.49 (m, 4H), 3.38 – 3.34 (m, 4H), 2.63 (t, *J* = 5.8 Hz, 2H), 2.13 (q, *J* = 7.5 Hz, 2H), 0.97 (t, *J* = 7.5 Hz, 3H).

^{13}C NMR: (101 MHz, d_6-DMSO) δ 167.0, 162.5, 162.1, 144.4, 139.5, 131.2, 127.7, 122.7, 115.4, 104.5, 72.9, 69.7, 69.6, 68.4, 42.2, 41.2, 27.5, 13.2.

HRMS (ESI): calc. for $C_{18}H_{28}N_7O_4$ $[M + H]^+$: 406.2197, found: 406.2192.

MP: 134 – 136 °C.

Supplemental References

1. Chen M, Rahman L, Voeller D, Kastanos E, Yang SX, Feigenbaum L, et al. Transgenic expression of human thymidylate synthase accelerates the development of hyperplasia and tumors in the endocrine pancreas. *Oncogene.* 2007;26(33):4817-24.
2. Allegra CJ, Chabner BA, Drake JC, Lutz R, Rodbard D, and Jolivet J. Enhanced inhibition of thymidylate synthase by methotrexate polyglutamates. *J Biol Chem.* 1985;260(17):9720-6.
3. Rahman L, Voeller D, Rahman M, Lipkowitz S, Allegra C, Barrett JC, et al. Thymidylate synthase as an oncogene: a novel role for an essential DNA synthesis enzyme. *Cancer Cell.* 2004;5(4):341-51.
4. Tetko IV, Tanchuk VY, Kasheva TN, and Villa AE. Estimation of aqueous solubility of chemical compounds using E-state indices. *J Chem Inf Comput Sci.* 2001;41(6):1488-93.
5. Humphrey W, Dalke A, and Schulten K. VMD: visual molecular dynamics. *J Mol Graph.* 1996;14(1):33-8, 27-8.

6. Morris GM, Huey R, Lindstrom W, Sanner MF, Belew RK, Goodsell DS, et al. AutoDock4 and AutoDockTools4: Automated docking with selective receptor flexibility. *J Comput Chem.* 2009;30(16):2785-91.
7. Arcon JP, Modenutti CP, Avendano D, Lopez ED, Defelipe LA, Ambrosio FA, et al. AutoDock Bias: improving binding mode prediction and virtual screening using known protein-ligand interactions. *Bioinformatics.* 2019;35(19):3836-8.
8. Arcon JP, Defelipe LA, Modenutti CP, Lopez ED, Alvarez-Garcia D, Barril X, et al. Molecular Dynamics in Mixed Solvents Reveals Protein-Ligand Interactions, Improves Docking, and Allows Accurate Binding Free Energy Predictions. *J Chem Inf Model.* 2017;57(4):846-63.
9. Arcon JP, Defelipe LA, Lopez ED, Burastero O, Modenutti CP, Barril X, et al. Cosolvent-Based Protein Pharmacophore for Ligand Enrichment in Virtual Screening. *J Chem Inf Model.* 2019;59(8):3572-83.
10. Stierand K, Maass PC, and Rarey M. Molecular complexes at a glance: automated generation of two-dimensional complex diagrams. *Bioinformatics.* 2006;22(14):1710-6.
11. Radusky LG, and Serrano L. pyFoldX: enabling biomolecular analysis and engineering along structural ensembles. *Bioinformatics.* 2022;38(8):2353-5.
12. Tong Y, Liu-Chen X, Ercikan-Abali EA, Zhao SC, Banerjee D, Maley F, et al. Probing the folate-binding site of human thymidylate synthase by site-directed mutagenesis. Generation of mutants that confer resistance to raltitrexed, Thymitaq, and BW1843U89. *J Biol Chem.* 1998;273(47):31209-14.
13. Lamb KM, N GD, Wright DL, and Anderson AC. Elucidating features that drive the design of selective antifolates using crystal structures of human dihydrofolate reductase. *Biochemistry.* 2013;52(41):7318-26.
14. Griffin RJ, Meek MA, Schwalbe CH, and Stevens MF. Structural studies on bioactive compounds. 8. Synthesis, crystal structure, and biological properties of a new series of 2,4-diamino-5-aryl-6-ethylpyrimidine dihydrofolate reductase inhibitors with in vivo activity against a methotrexate-resistant tumor cell line. *J Med Chem.* 1989;32(11):2468-74.

B) <u>NMR Spectra.</u>

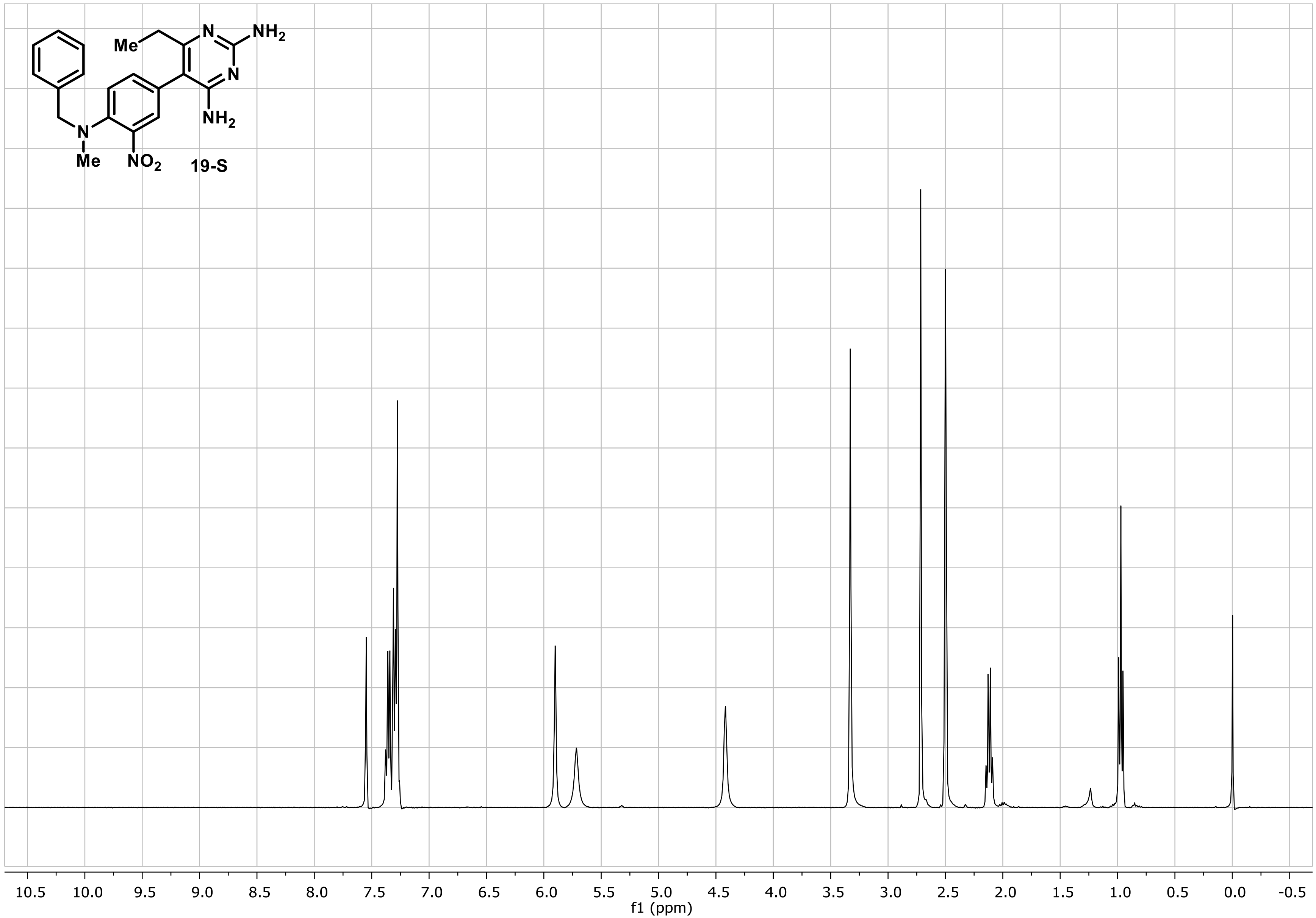
19-S
10.5 10.0 9.5 9.0 8.5 8.0 7.5 7.0 6.5 6.0 5.5 5.0 4.5 4.0 3.5 3.0 2.5 2.0 1.5 1.0 0.5 0.0 -0.5
f1 (ppm)

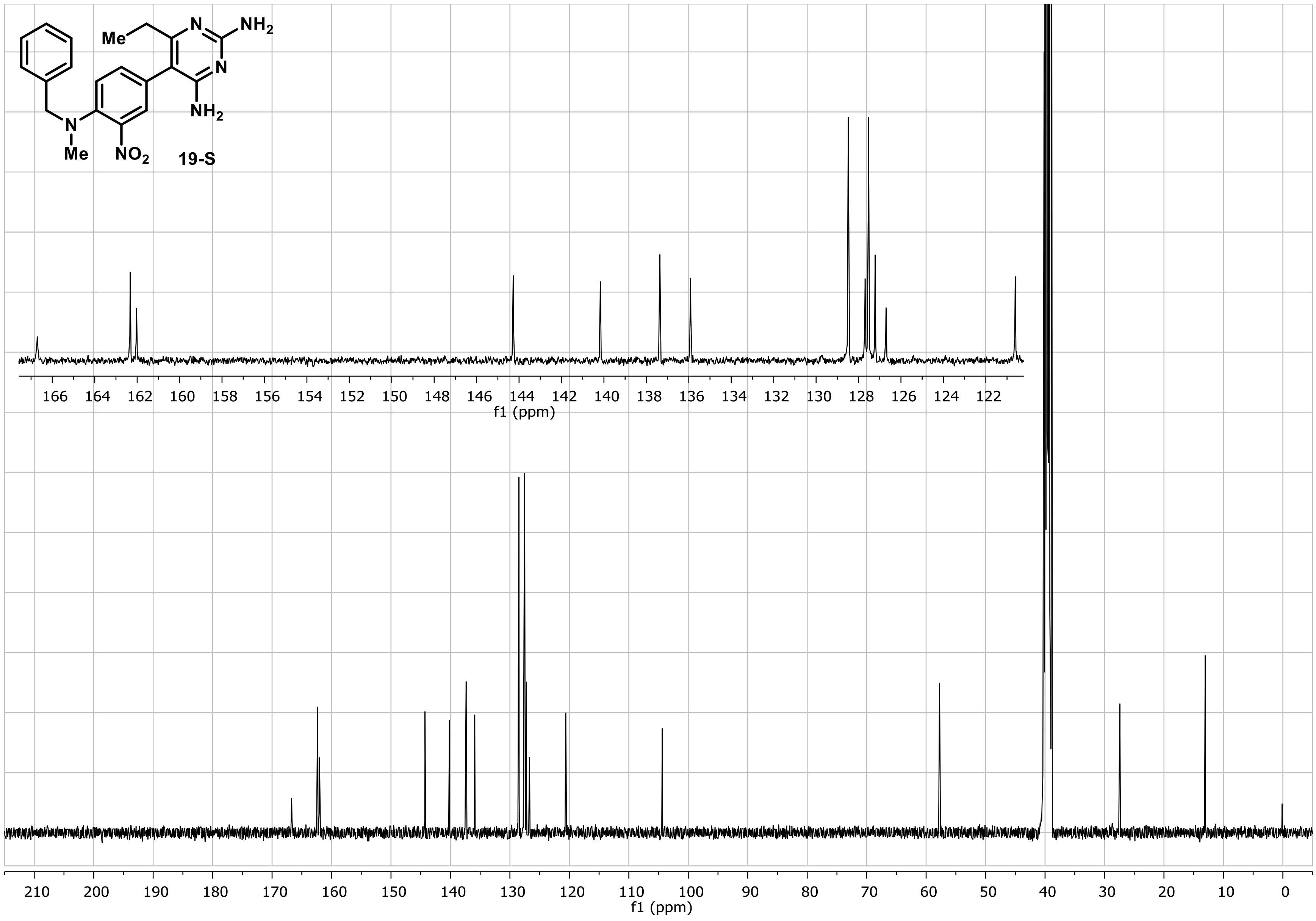
Me
N
NH2
N
NH2
N
Me
NO2
19-S
f1 (ppm)
f1 (ppm)

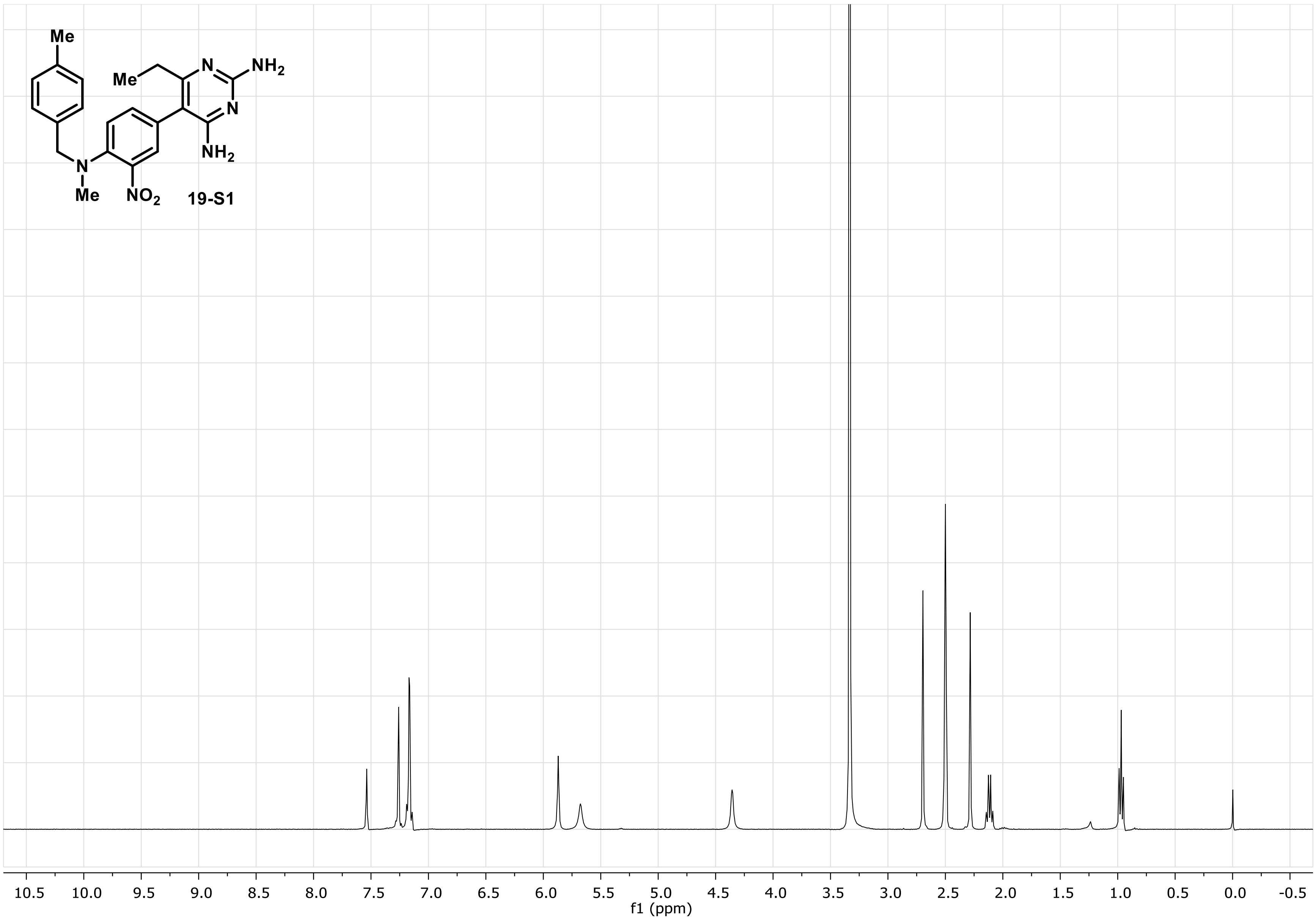
Me
Me
N
NH2
N
NH2
N
Me
NO2
19-S1
10.5
10.0
9.5
9.0
8.5
8.0
7.5
7.0
6.5
6.0
5.5
5.0
4.5
4.0
3.5
3.0
2.5
2.0
1.5
1.0
0.5
0.0
-0.5
f1 (ppm)

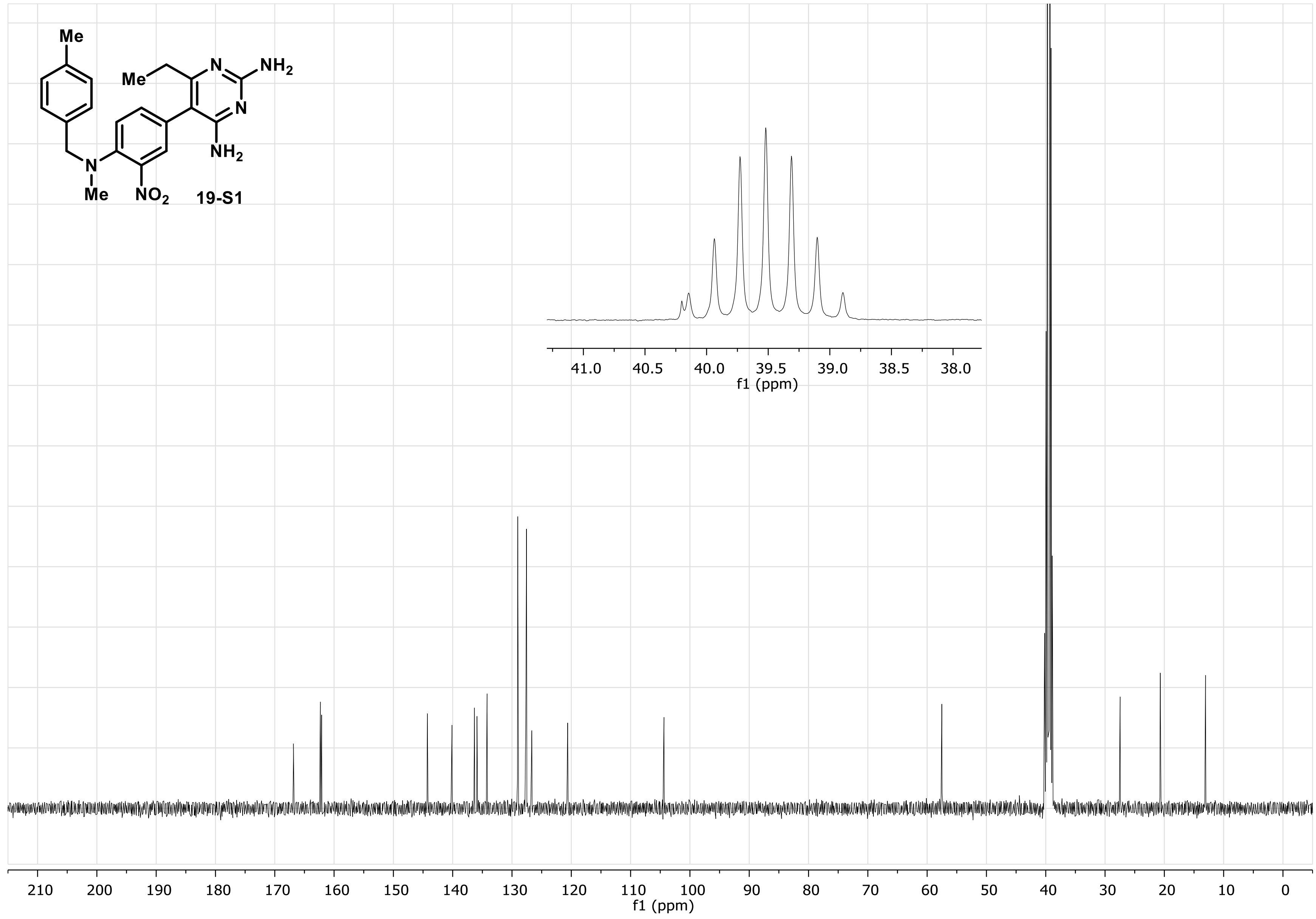
19-S1
Me
NH2
NO2
f1 (ppm)
41.0 40.5 40.0 39.5 39.0 38.5 38.0
210 200 190 180 170 160 150 140 130 120 110 100 90 80 70 60 50 40 30 20 10 0

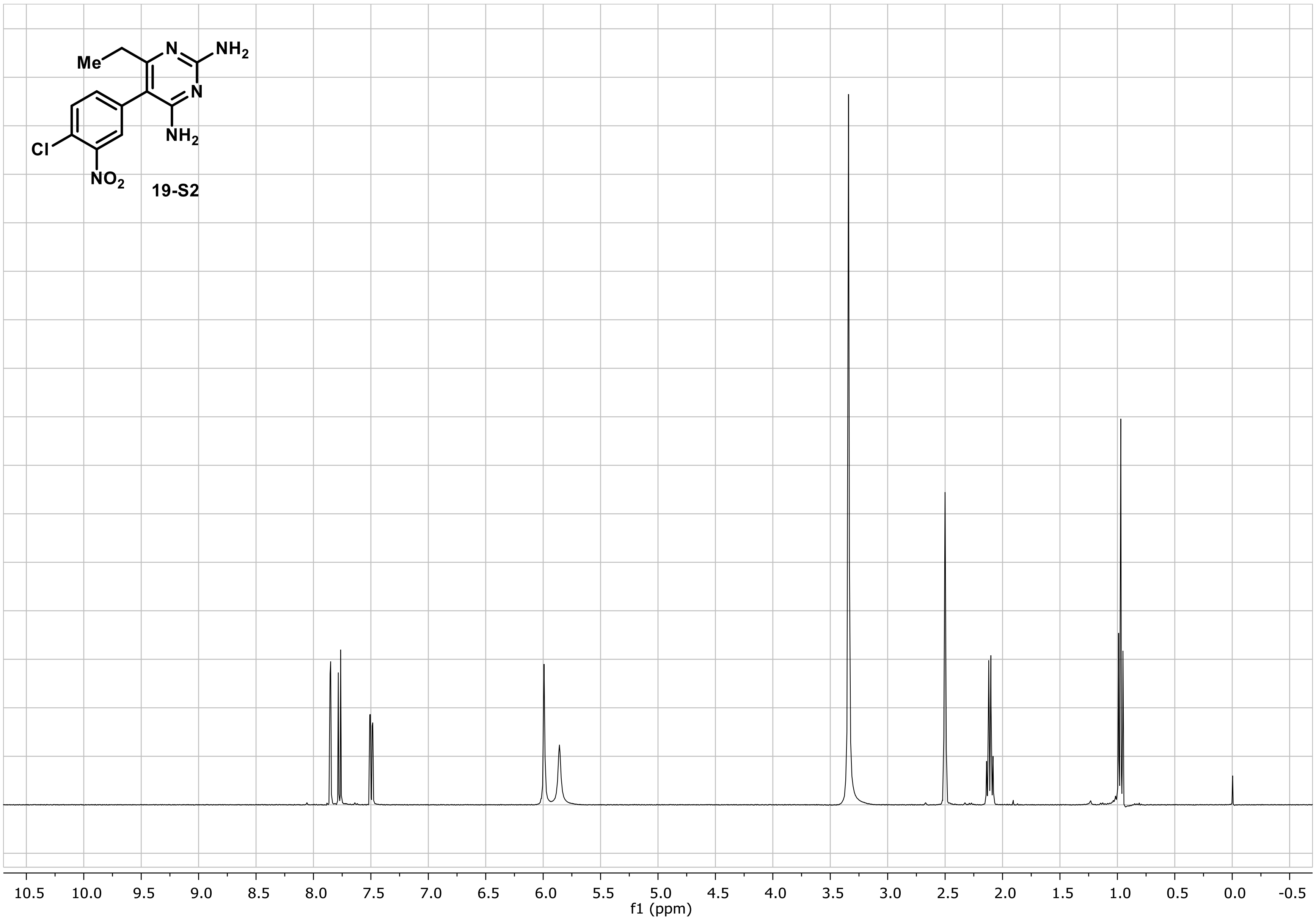
Me
N
NH2
N
NH2
Cl
NO2
19-S2
10.5 10.0 9.5 9.0 8.5 8.0 7.5 7.0 6.5 6.0 5.5 5.0 4.5 4.0 3.5 3.0 2.5 2.0 1.5 1.0 0.5 0.0 -0.5
f1 (ppm)

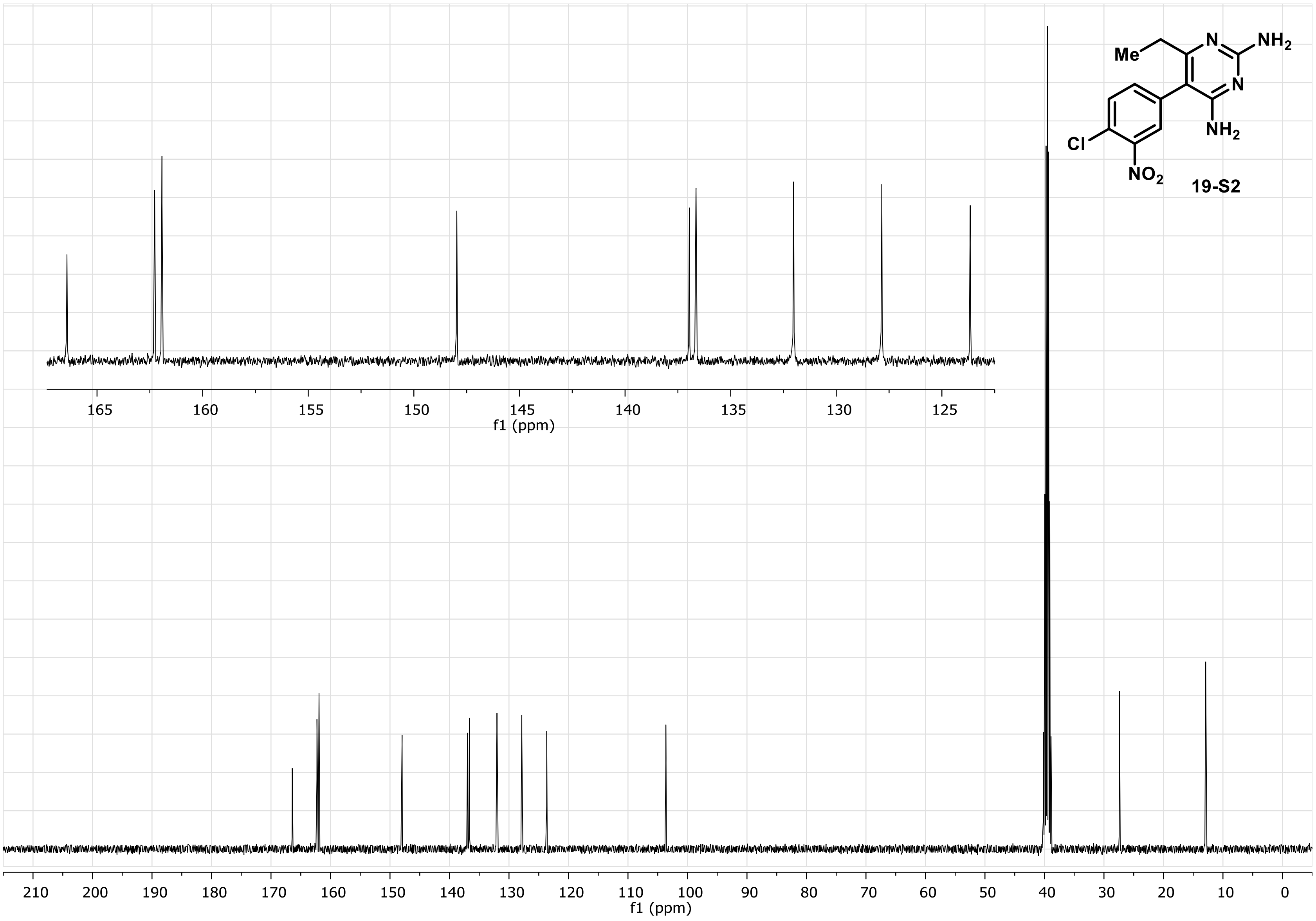
Me
N
NH2
N
NH2
Cl
NO2
19-S2
165
160
155
150
145
140
135
130
125
f1 (ppm)
210
200
190
180
170
160
150
140
130
120
110
100
90
80
70
60
50
40
30
20
10
0
f1 (ppm)

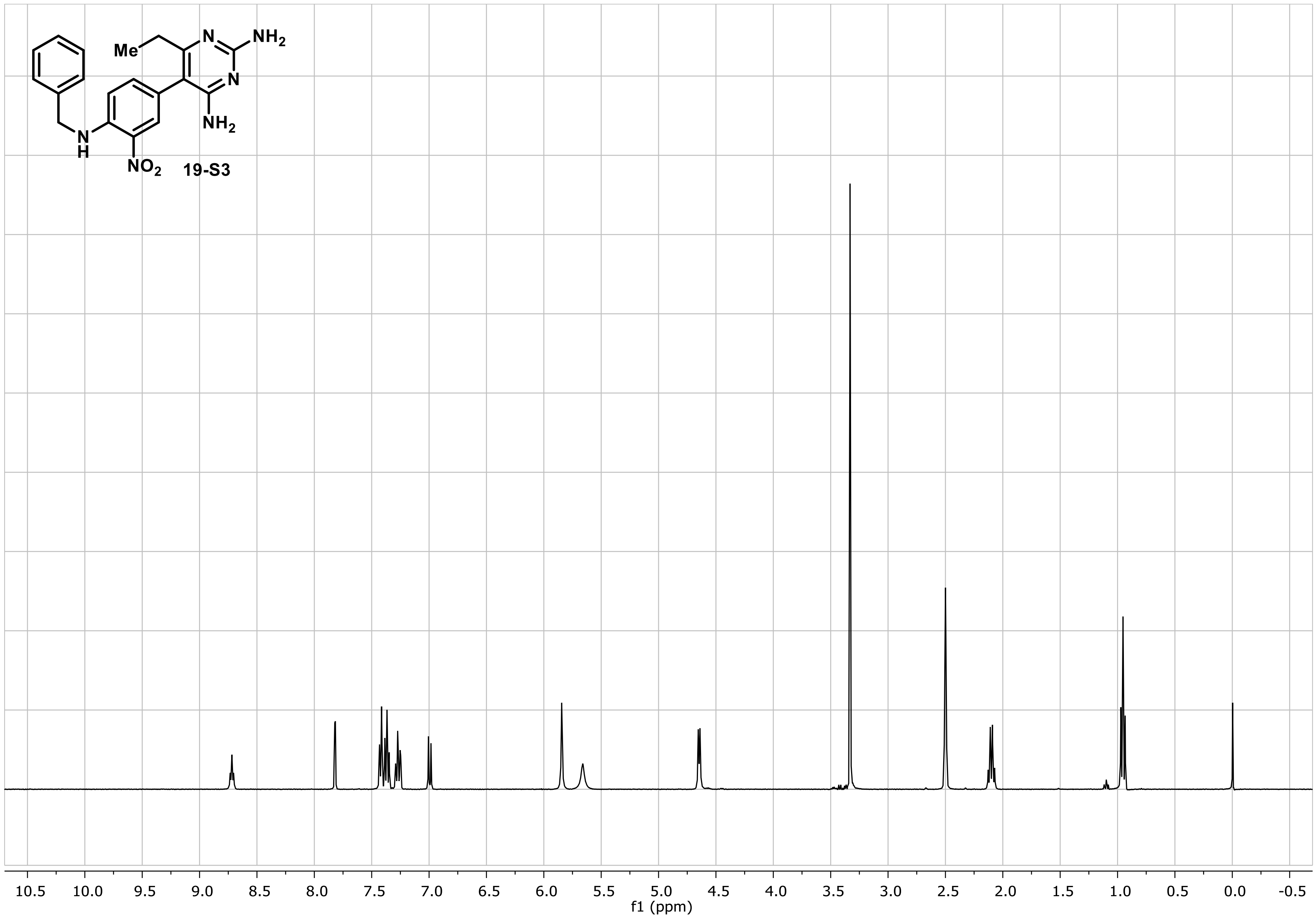

Me
N
NH2
N
NH2
N
H
NO2
19-S3
10.5 10.0 9.5 9.0 8.5 8.0 7.5 7.0 6.5 6.0 5.5 5.0 4.5 4.0 3.5 3.0 2.5 2.0 1.5 1.0 0.5 0.0 -0.5
f1 (ppm)

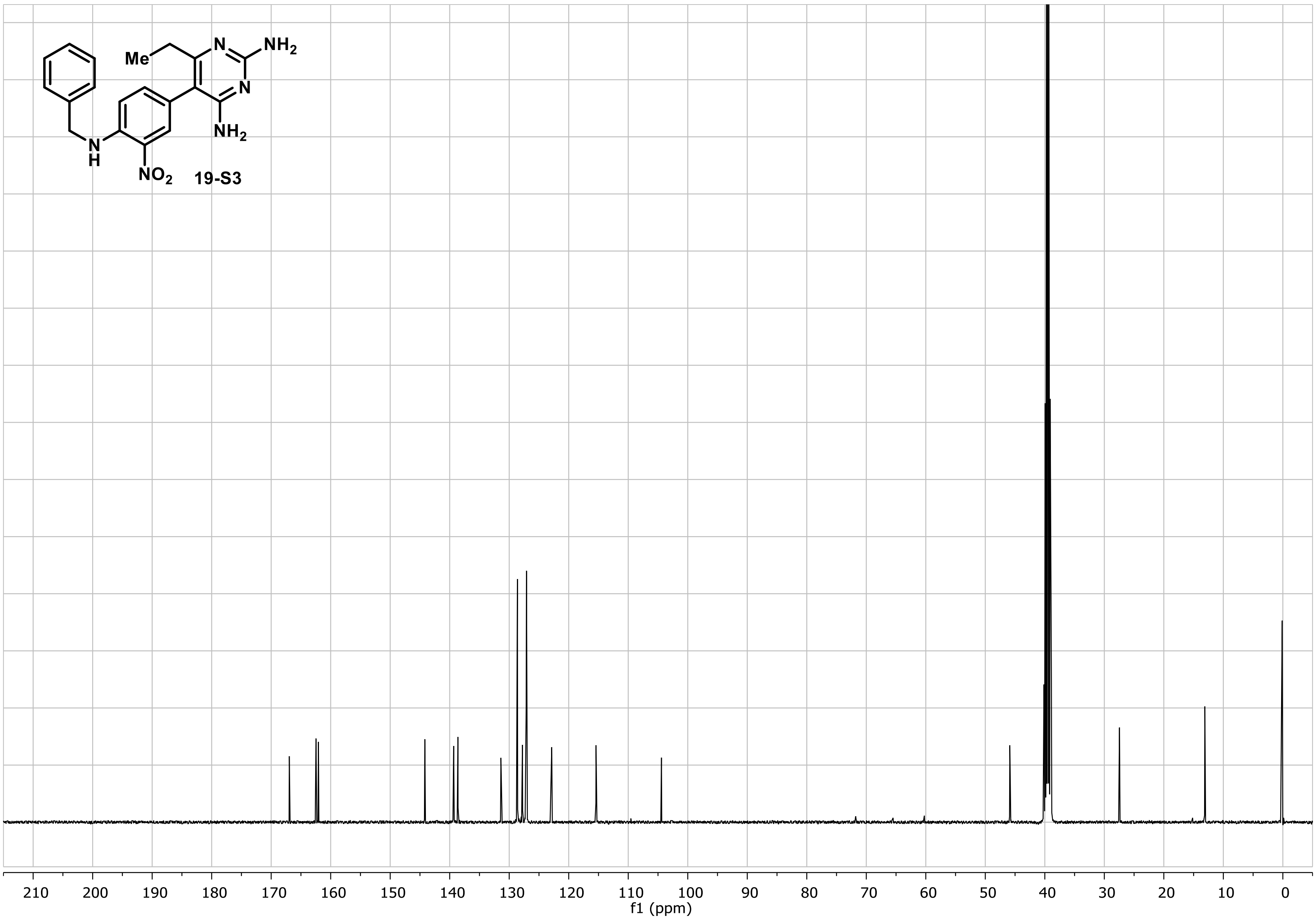

Me
N
NH2
N
NH2
N
H
NO2
19-S3
210 200 190 180 170 160 150 140 130 120 110 100 90 80 70 60 50 40 30 20 10 0
f1 (ppm)

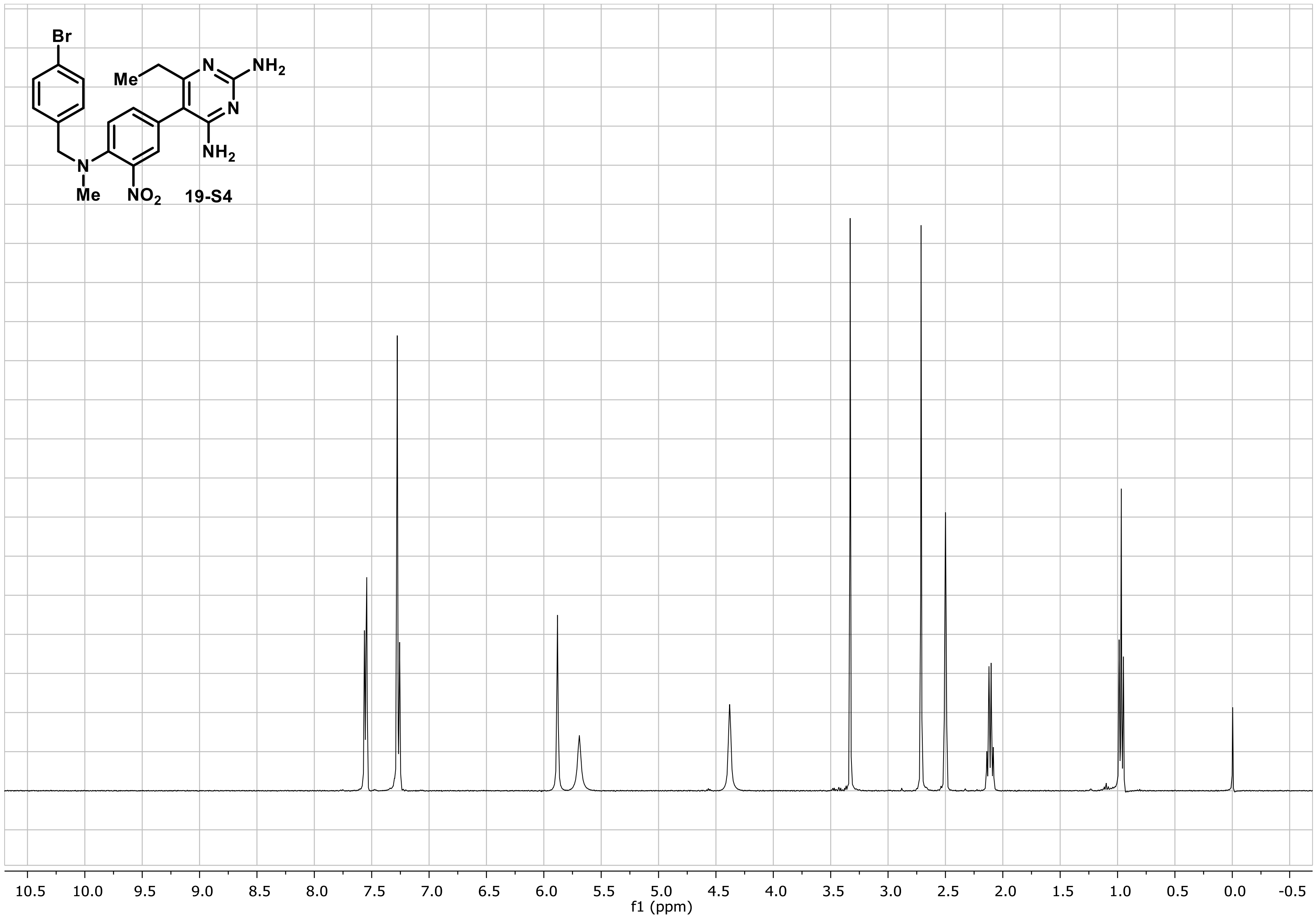
Br
Me
N
NH2
N
NH2
N
Me
NO2
19-S4
10.5
10.0
9.5
9.0
8.5
8.0
7.5
7.0
6.5
6.0
5.5
5.0
4.5
4.0
3.5
3.0
2.5
2.0
1.5
1.0
0.5
0.0
-0.5
f1 (ppm)

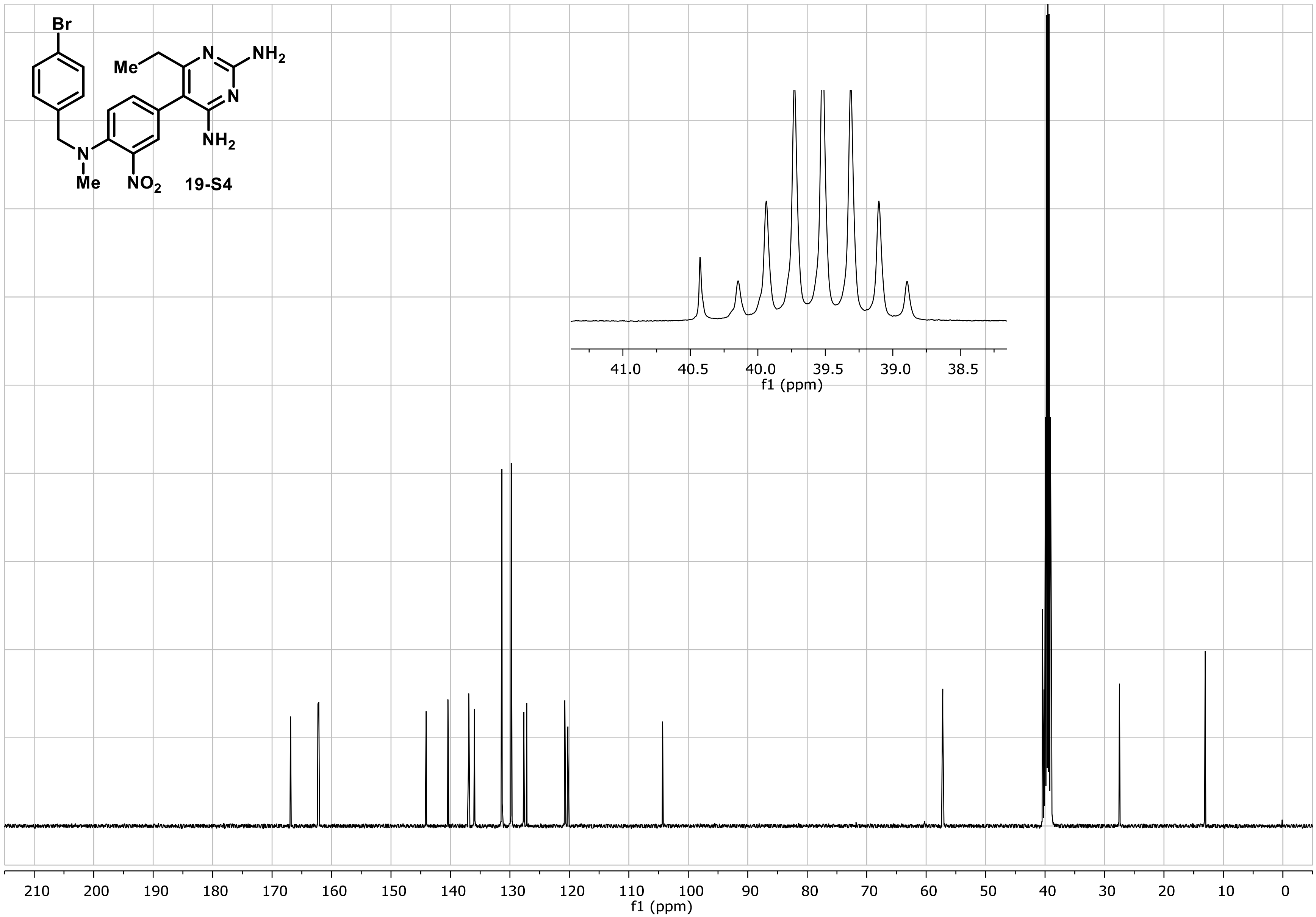
19-S4
41.0
40.5
40.0
39.5
39.0
38.5
f1 (ppm)
210
200
190
180
170
160
150
140
130
120
110
100
90
80
70
60
50
40
30
20
10
0
f1 (ppm)

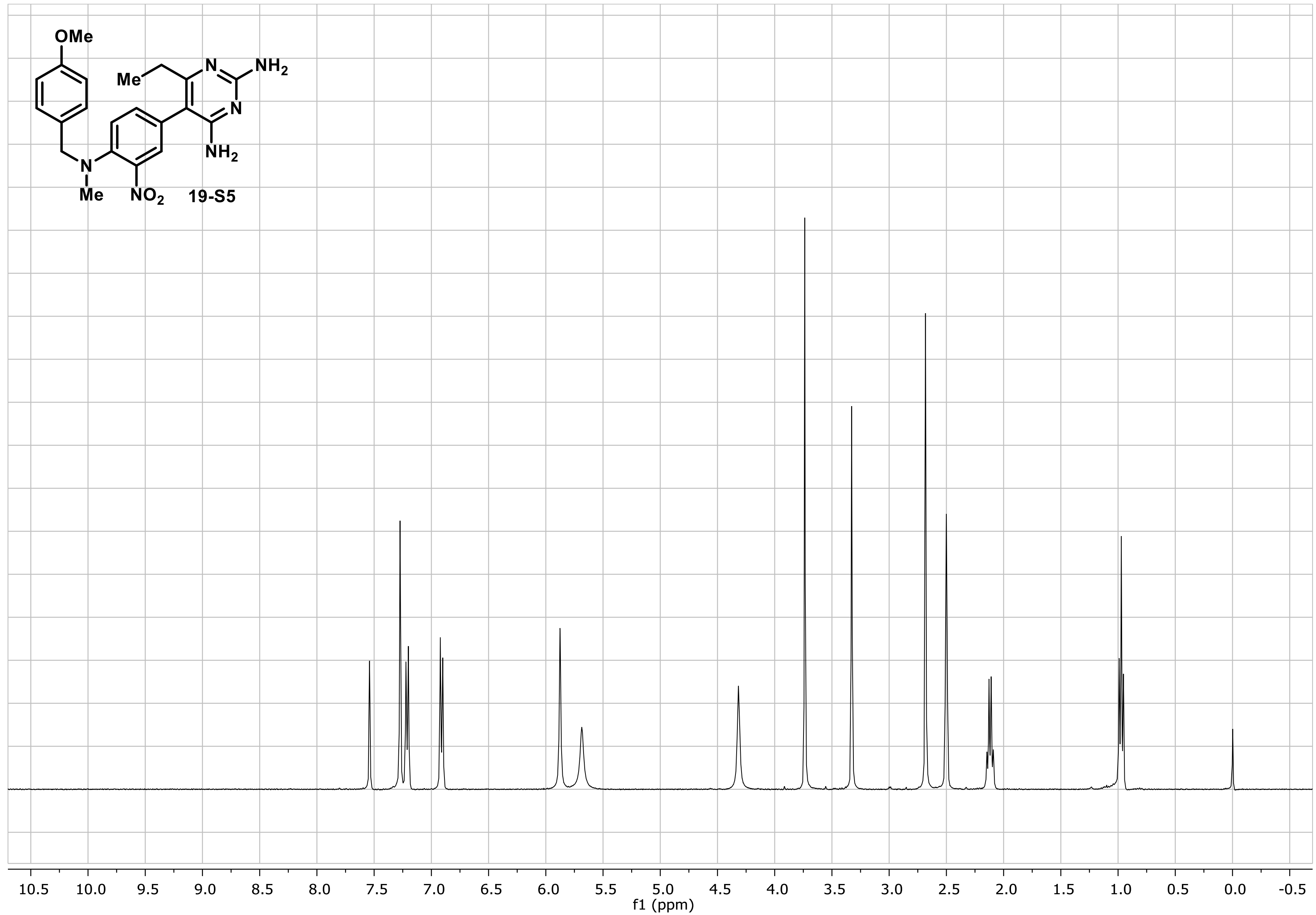
OMe
Me
N
NH2
N
N
Me
NO2
NH2
19-S5
10.5 10.0 9.5 9.0 8.5 8.0 7.5 7.0 6.5 6.0 5.5 5.0 4.5 4.0 3.5 3.0 2.5 2.0 1.5 1.0 0.5 0.0 -0.5
f1 (ppm)

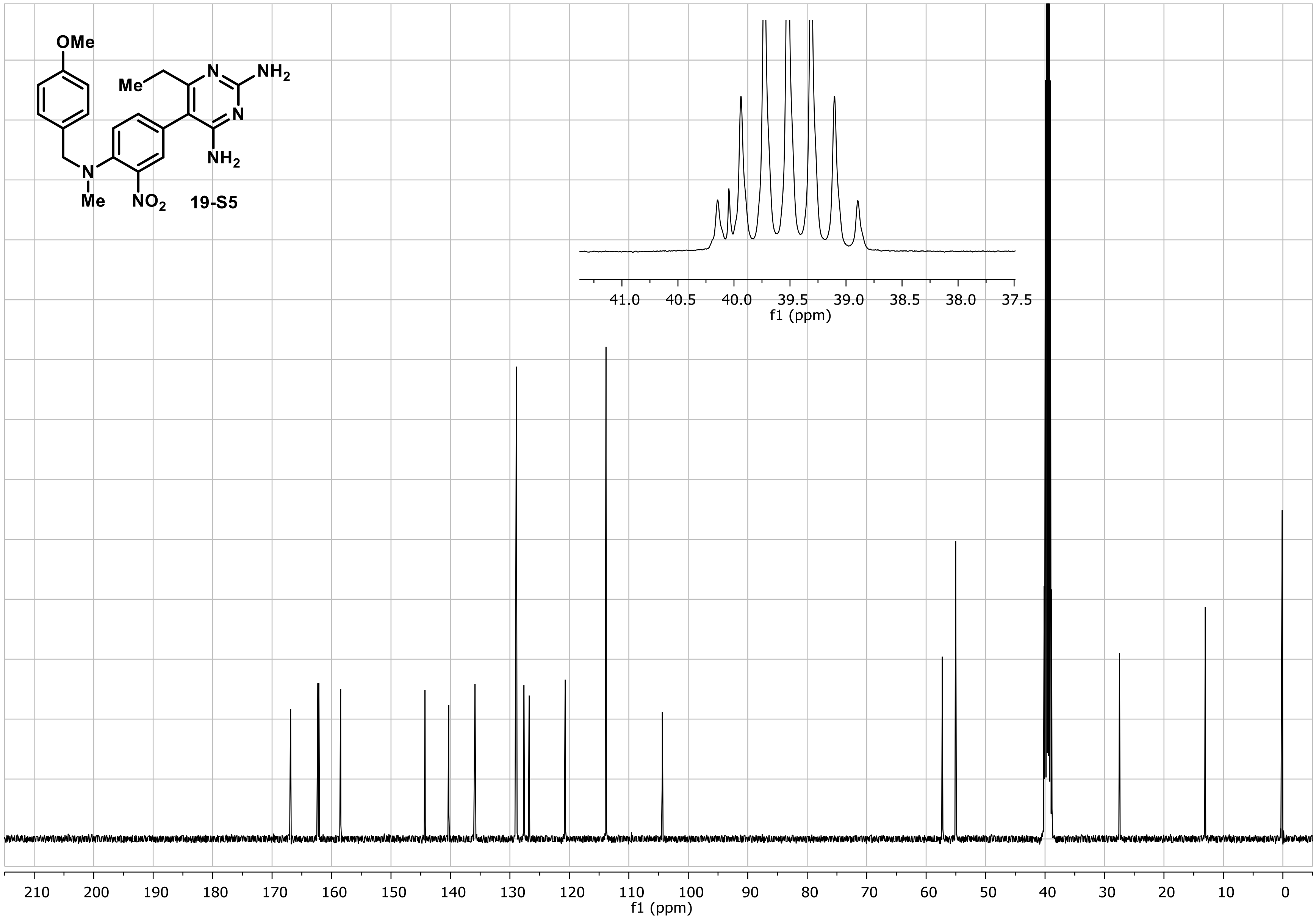
OMe
Me
N
NH2
N
NH2
N
Me
NO2
19-S5
41.0
40.5
40.0
39.5
39.0
38.5
38.0
37.5
f1 (ppm)
210
200
190
180
170
160
150
140
130
120
110
100
90
80
70
60
50
40
30
20
10
0
f1 (ppm)

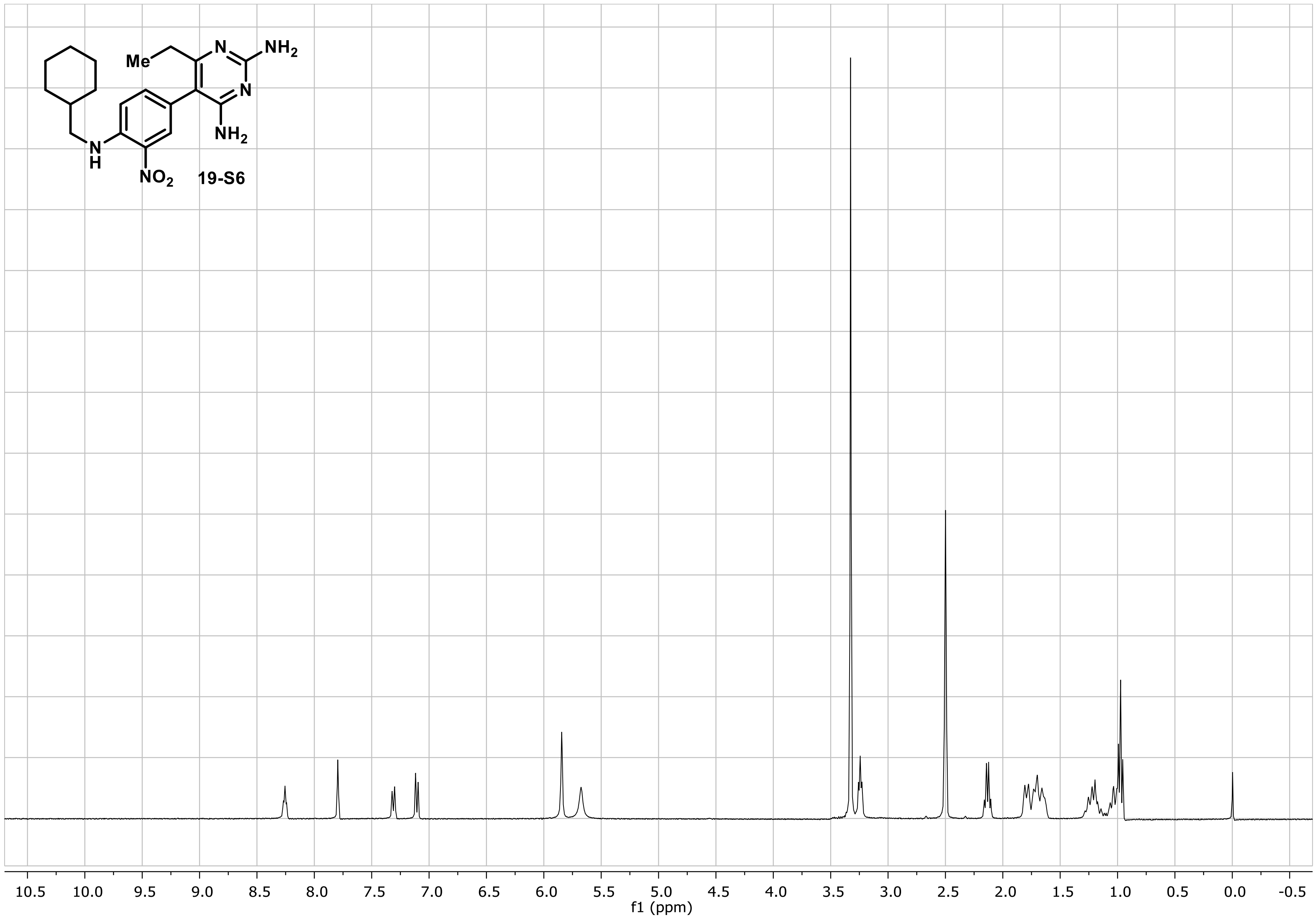
Me
N
NH2
N
NH2
N
H
NO2
19-S6
10.5 10.0 9.5 9.0 8.5 8.0 7.5 7.0 6.5 6.0 5.5 5.0 4.5 4.0 3.5 3.0 2.5 2.0 1.5 1.0 0.5 0.0 -0.5
f1 (ppm)

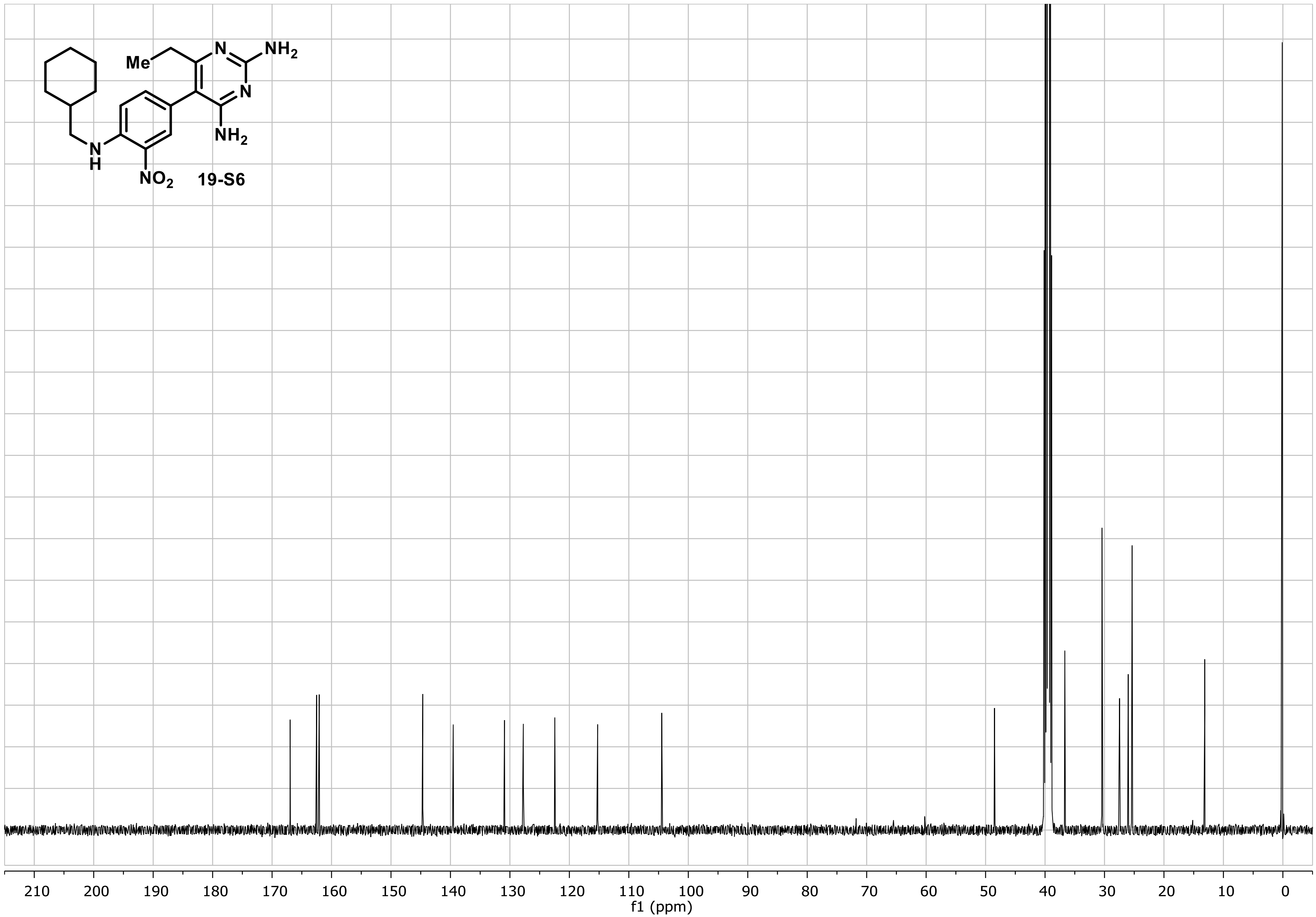
Me
N
NH2
N
NH2
N
H
NO2
19-S6
210 200 190 180 170 160 150 140 130 120 110 100 90 80 70 60 50 40 30 20 10 0
f1 (ppm)

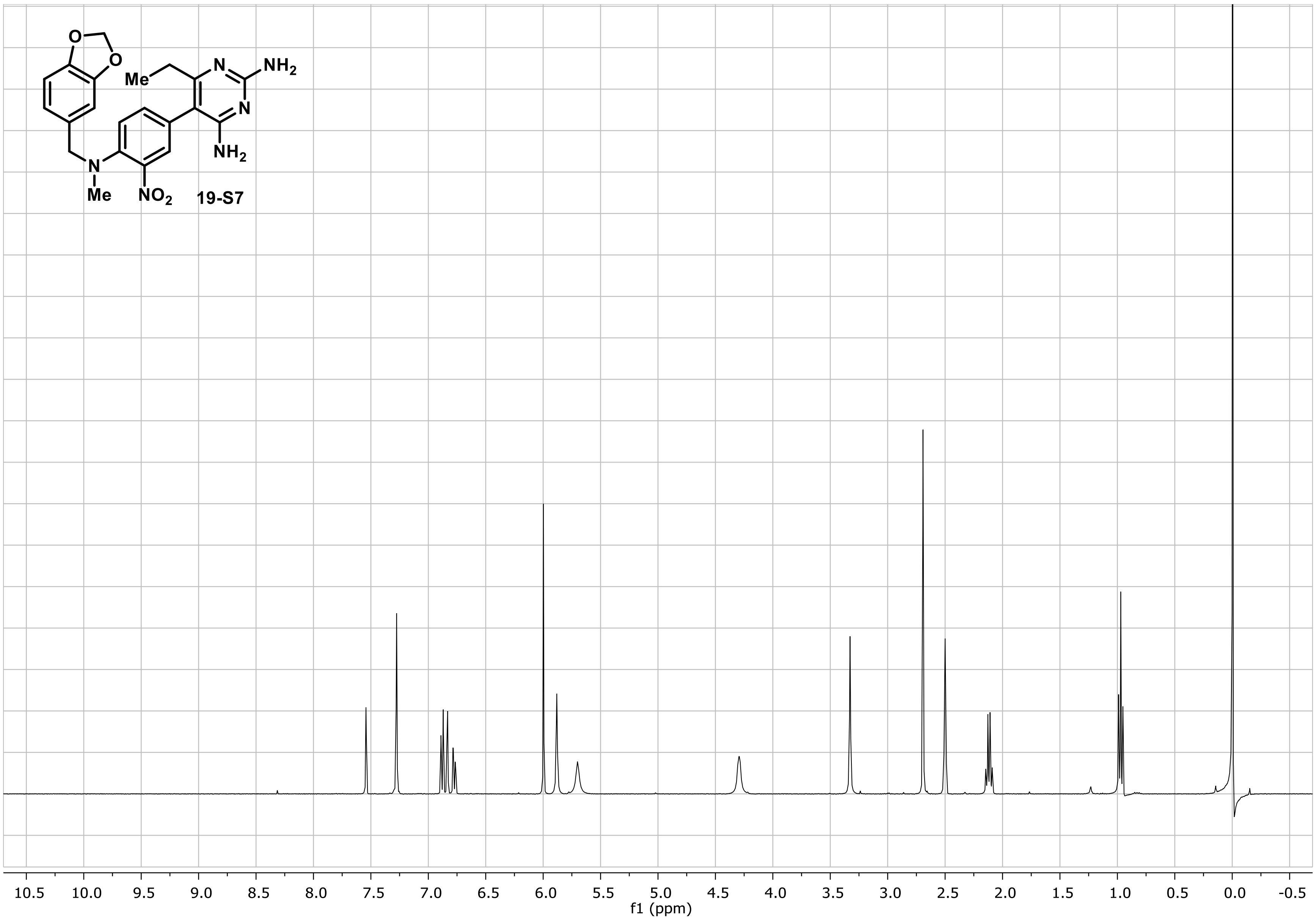

Me
N
NH2
N
NH2
N
Me
NO2
O
O
19-S7
10.5 10.0 9.5 9.0 8.5 8.0 7.5 7.0 6.5 6.0 5.5 5.0 4.5 4.0 3.5 3.0 2.5 2.0 1.5 1.0 0.5 0.0 -0.5
f1 (ppm)

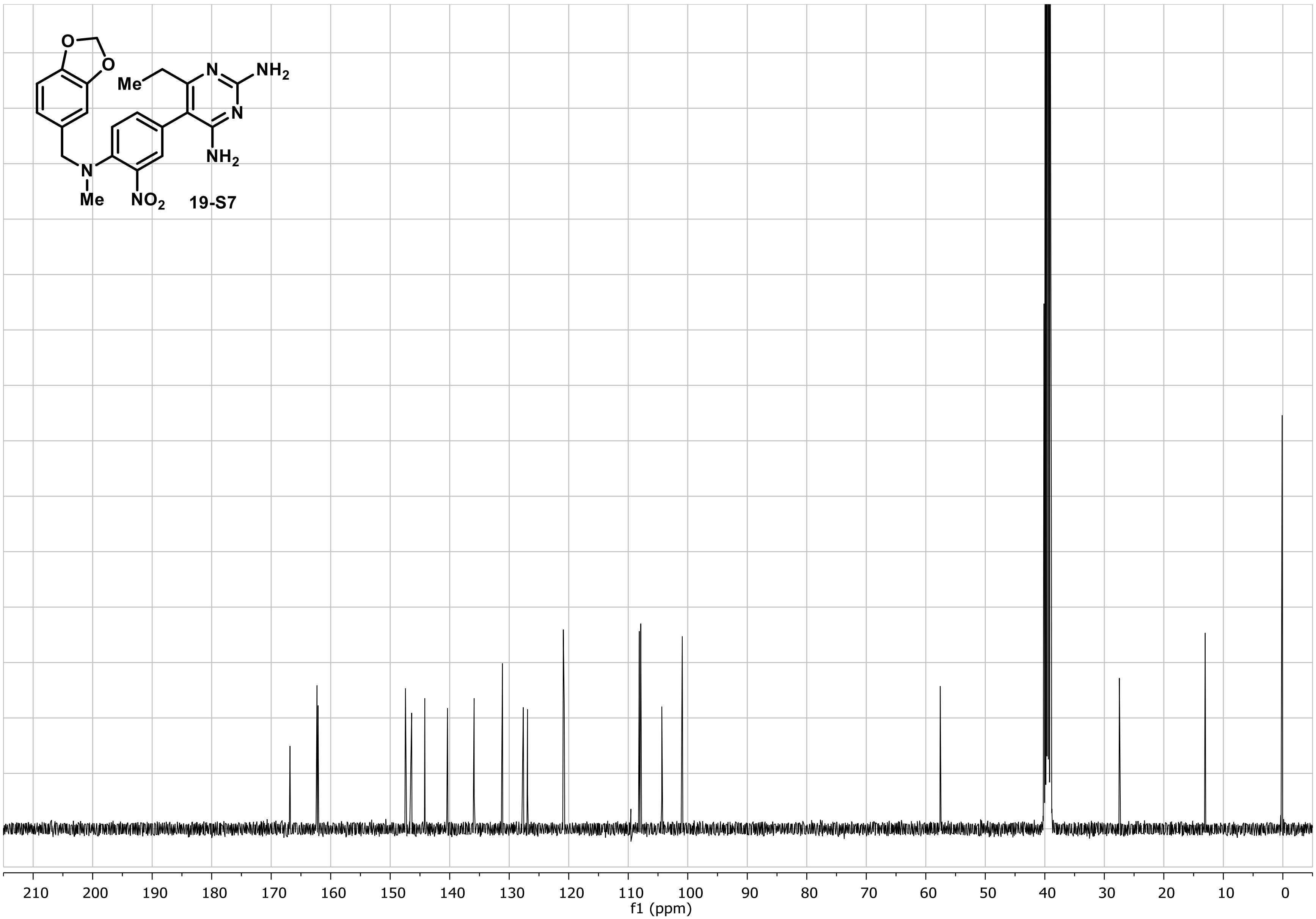

NH2
N
N
NH2
Me
O
O
N
Me
NO2
19-S7
210 200 190 180 170 160 150 140 130 120 110 100 90 80 70 60 50 40 30 20 10 0
f1 (ppm)

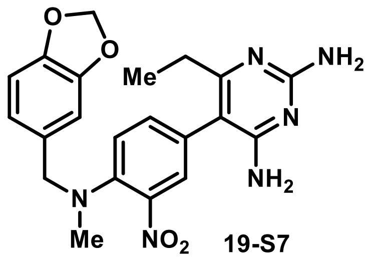

19-S7

HSQC

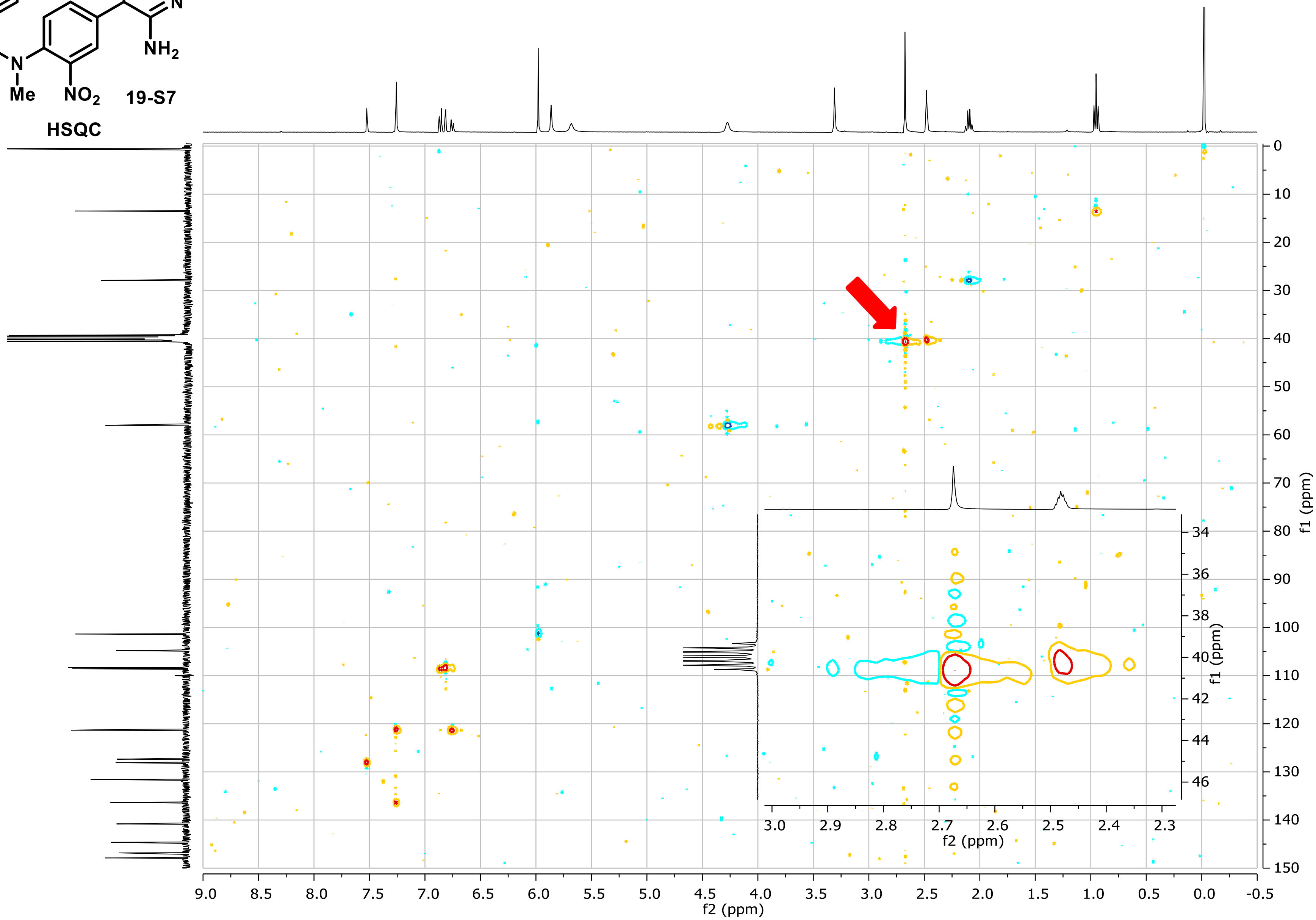

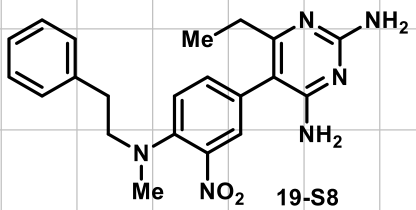

Me
NH2
N
N
NH2
N
Me
NO2

19-S8

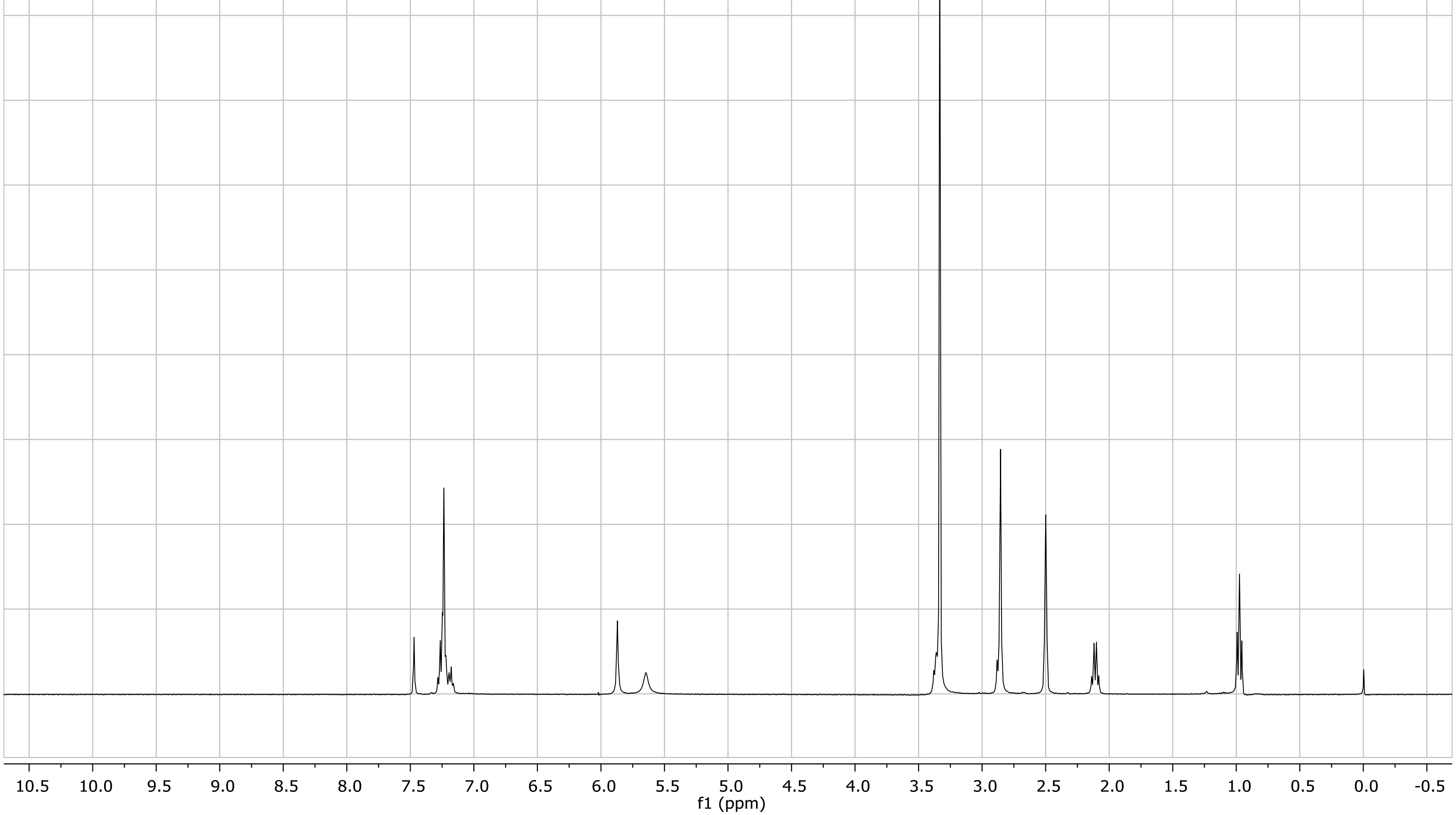

10.5
10.0
9.5
9.0
8.5
8.0
7.5
7.0
6.5
6.0
5.5
5.0
4.5
4.0
3.5
3.0
2.5
2.0
1.5
1.0
0.5
0.0
-0.5
f1 (ppm)

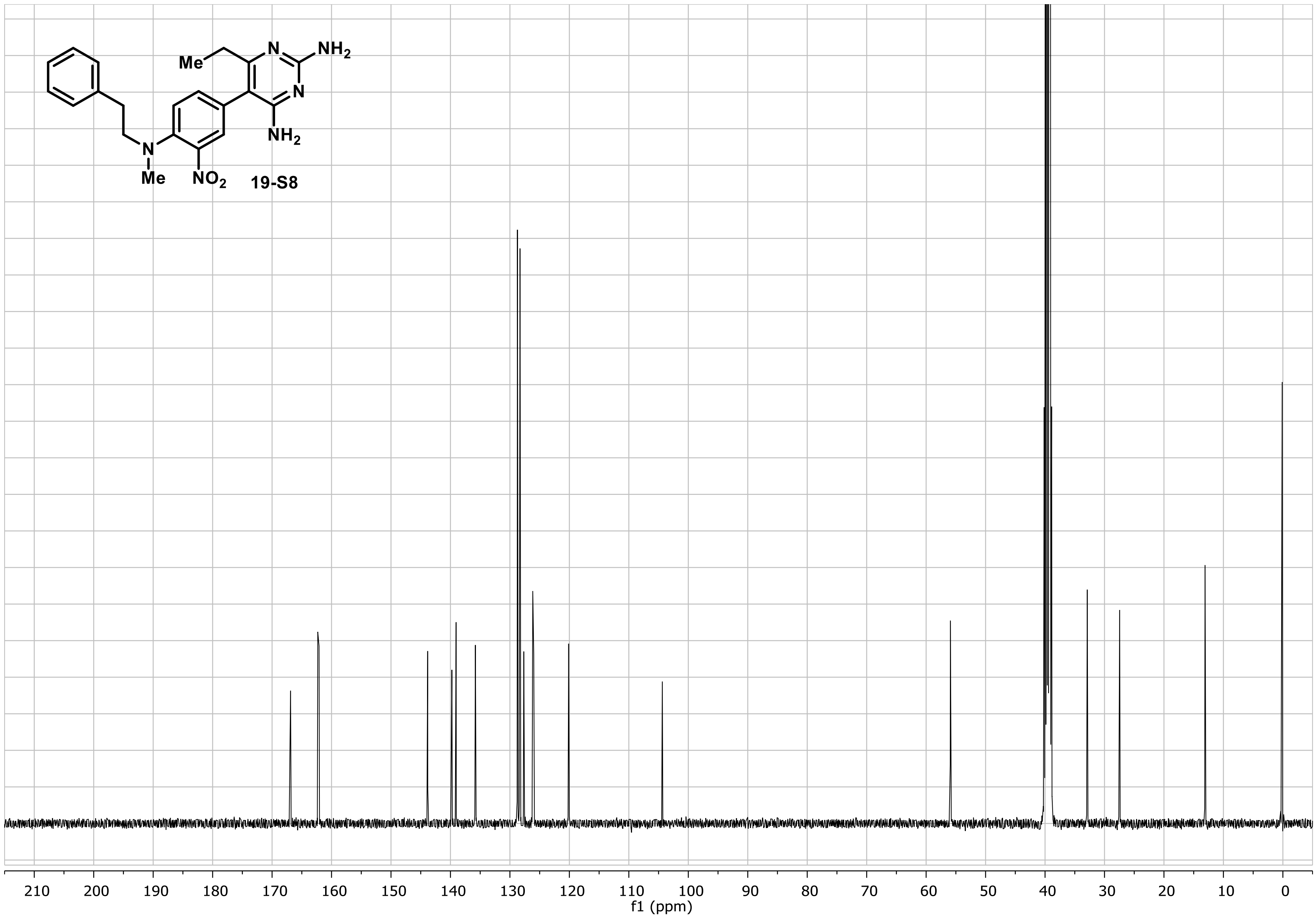
Me
NH2
NH2
N
Me
NO2
19-S8
210 200 190 180 170 160 150 140 130 120 110 100 90 80 70 60 50 40 30 20 10 0
f1 (ppm)

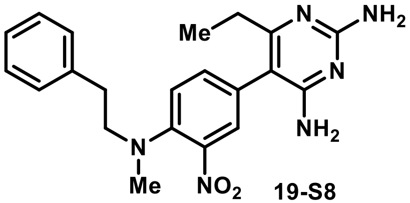

19-S8

HSQC

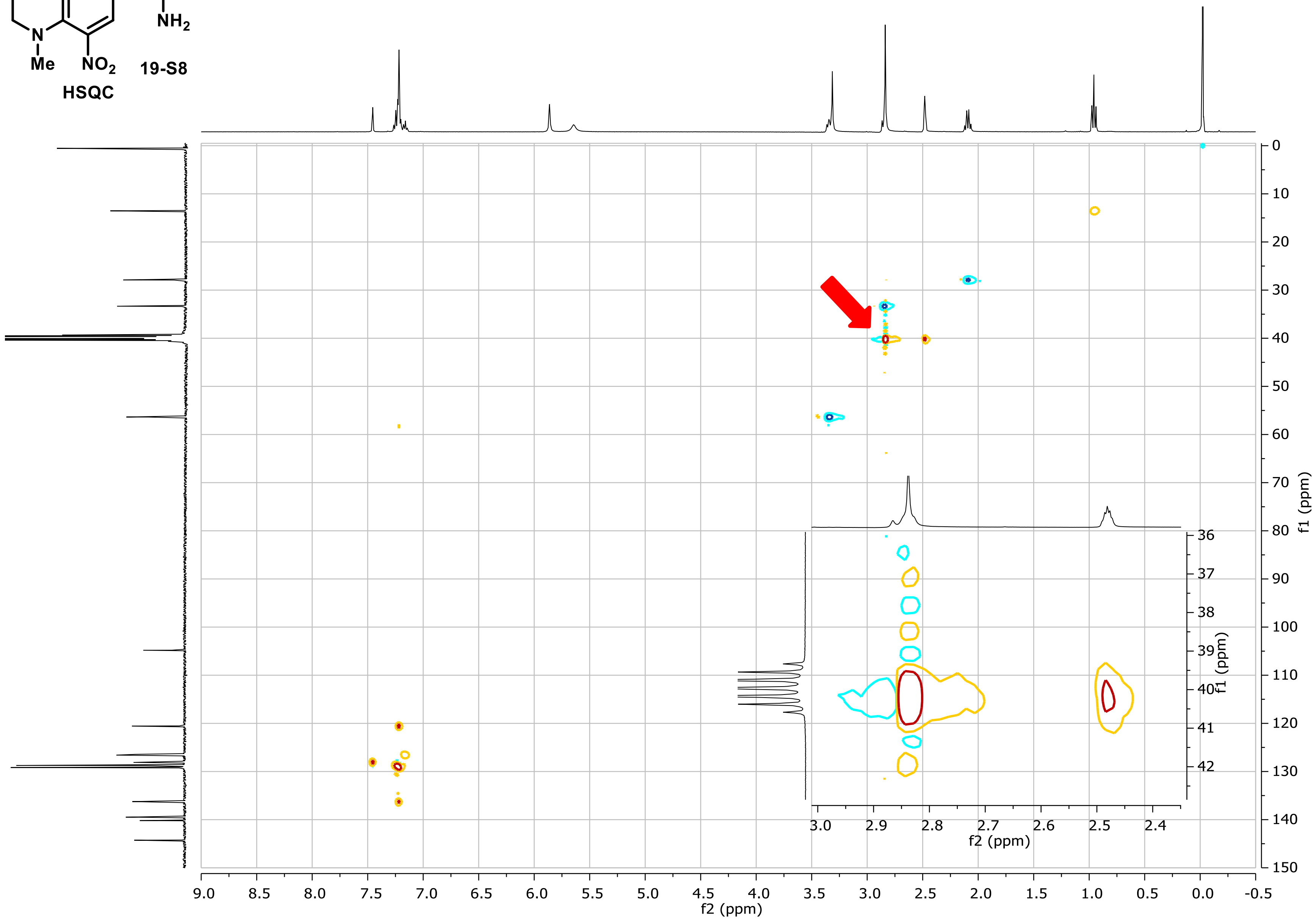

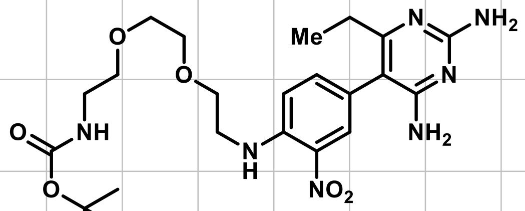
NH
O
O
O
N
H
NO2
Me
N
N
NH2
NH2

19-S9

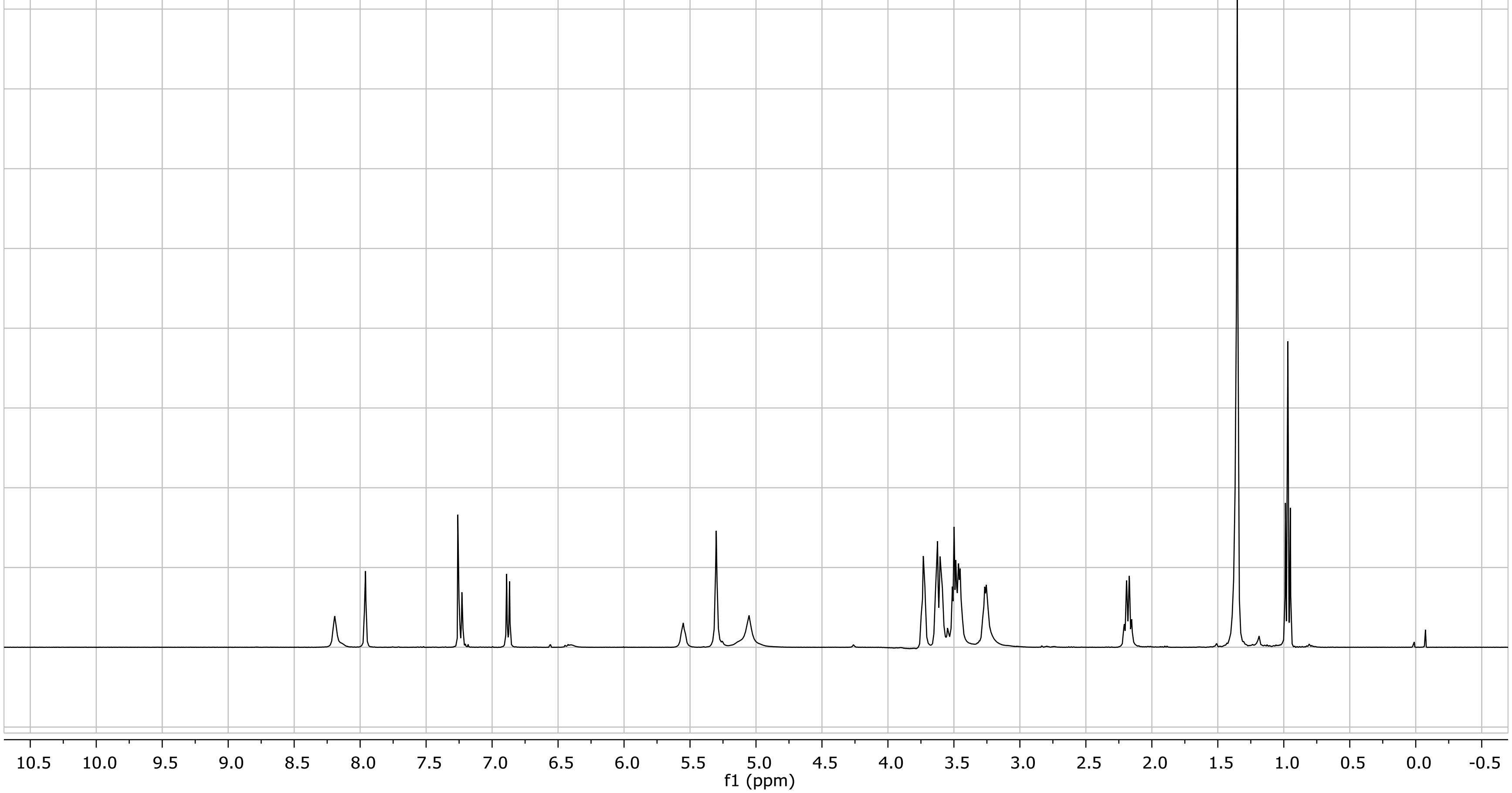
10.5
10.0
9.5
9.0
8.5
8.0
7.5
7.0
6.5
6.0
5.5
5.0
4.5
4.0
3.5
3.0
2.5
2.0
1.5
1.0
0.5
0.0
-0.5
f1 (ppm)

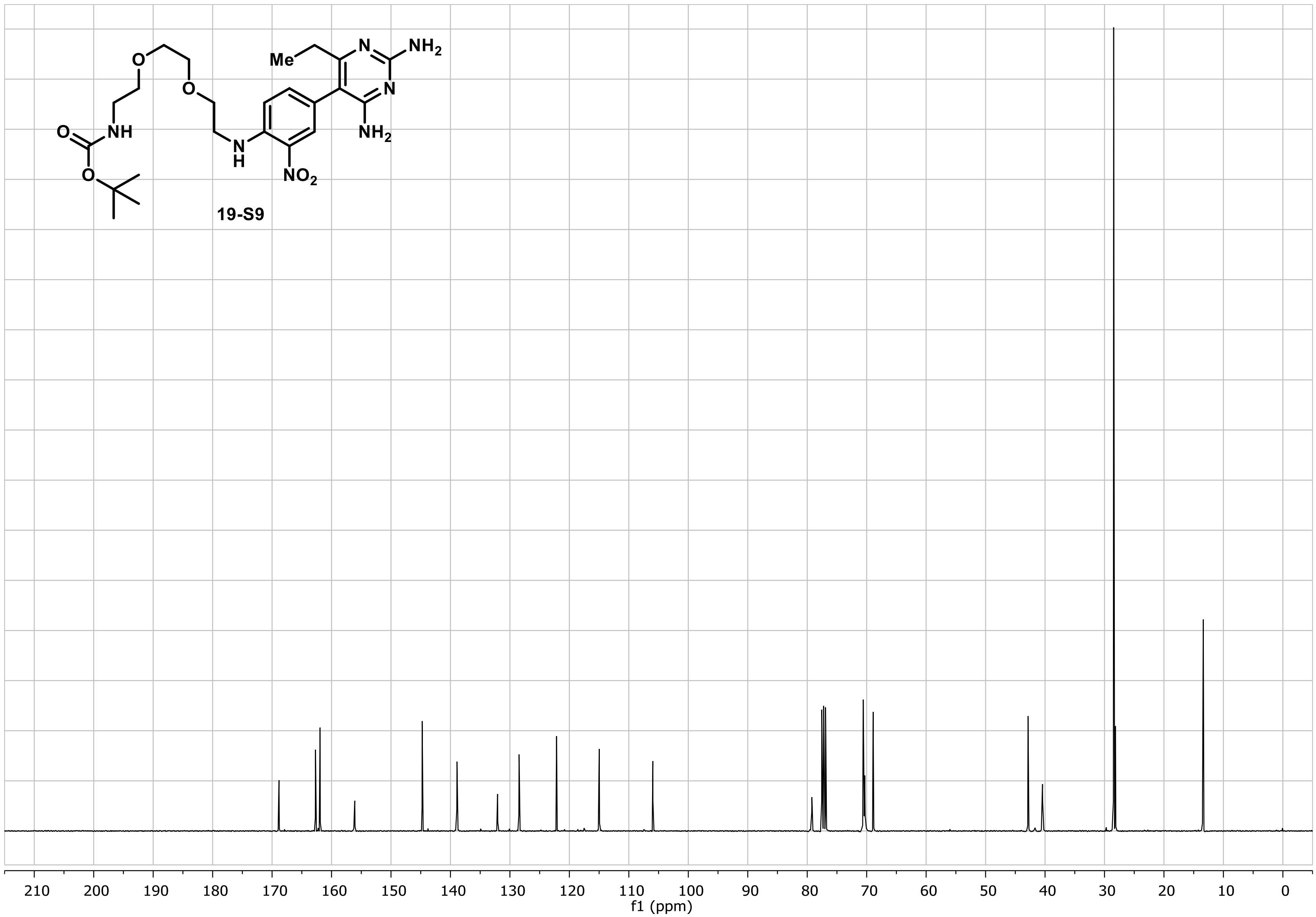

19-S9
Me
NH2
NO2
NH
O
210 200 190 180 170 160 150 140 130 120 110 100 90 80 70 60 50 40 30 20 10 0
f1 (ppm)

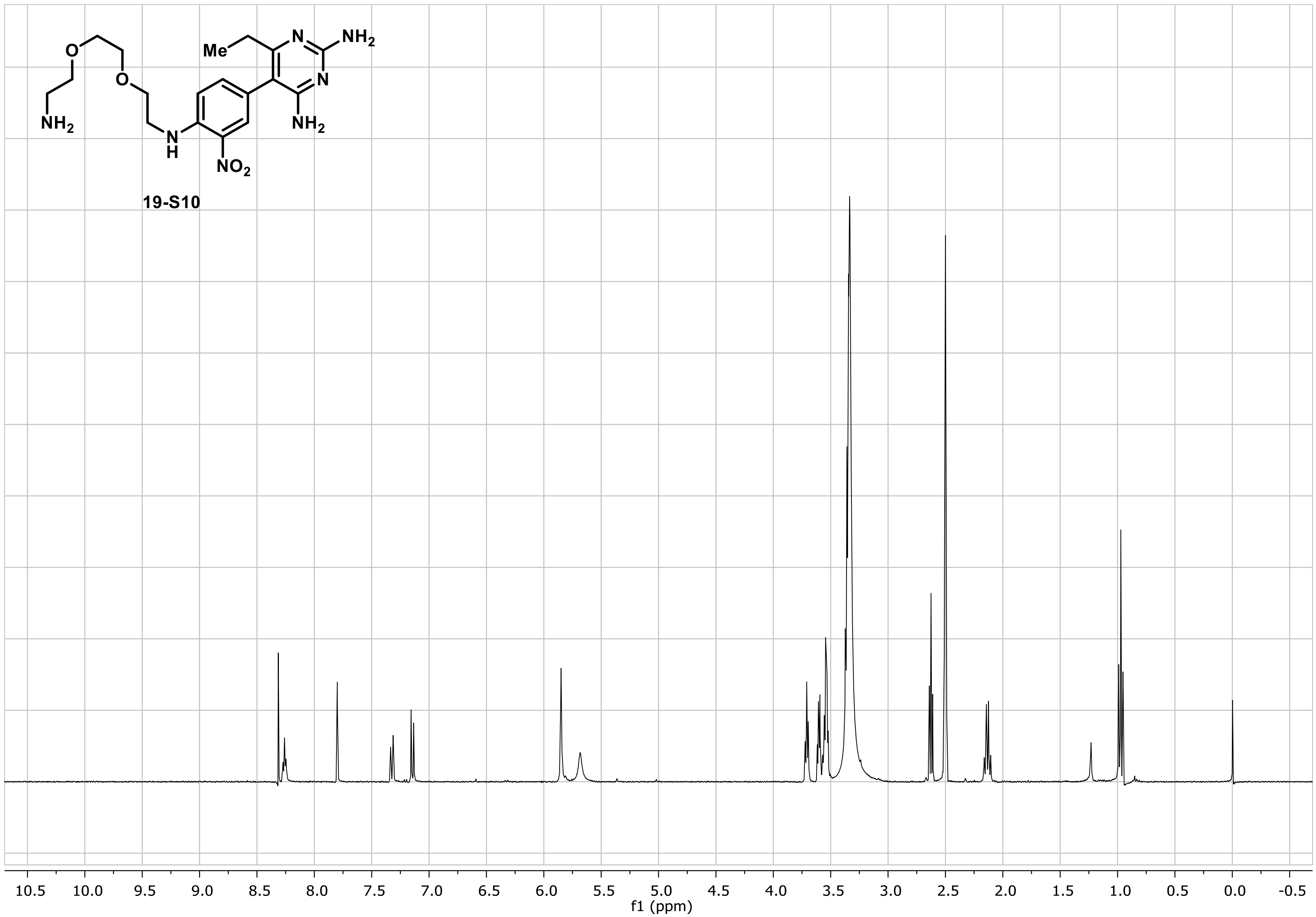
NH2
O
O
N
H
NO2
Me
N
NH2
N
NH2
19-S10
10.5
10.0
9.5
9.0
8.5
8.0
7.5
7.0
6.5
6.0
5.5
5.0
4.5
4.0
3.5
3.0
2.5
2.0
1.5
1.0
0.5
0.0
-0.5
f1 (ppm)

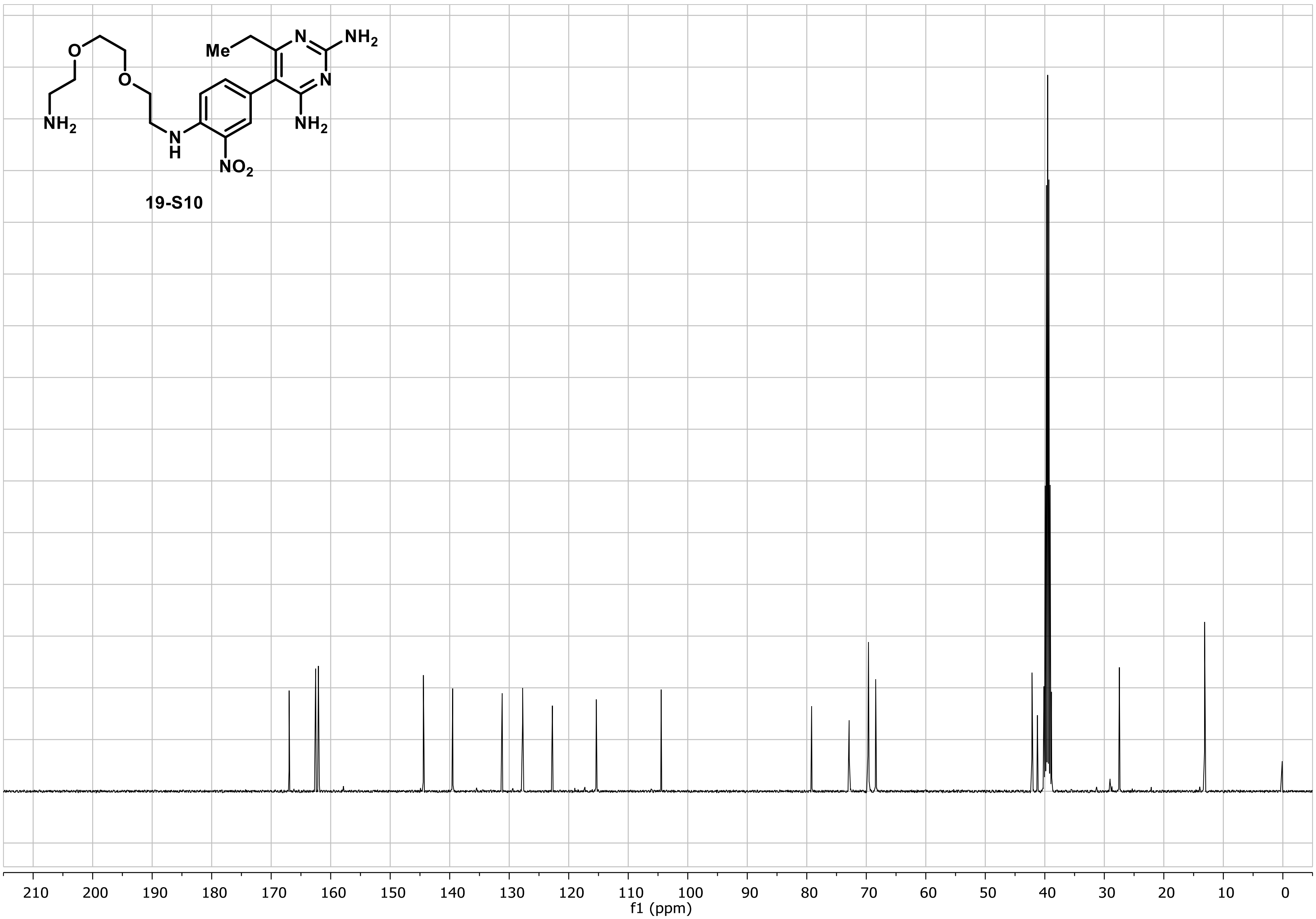

NH2
O
O
N
H
NO2
Me
N
NH2
N
NH2
19-S10
210 200 190 180 170 160 150 140 130 120 110 100 90 80 70 60 50 40 30 20 10 0
f1 (ppm)

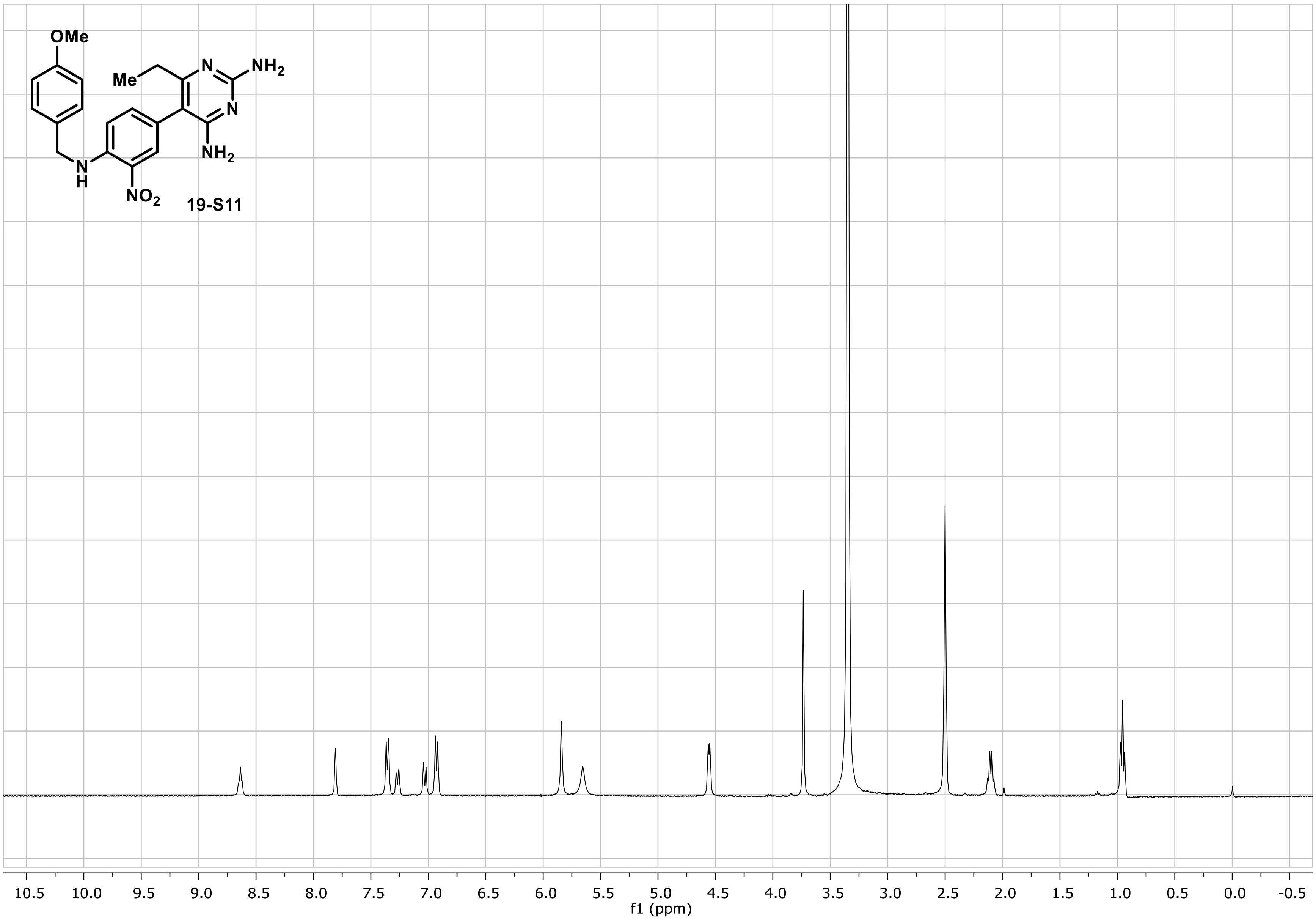

OMe
Me
N
NH2
N
NH2
N
H
NO2
19-S11
10.5
10.0
9.5
9.0
8.5
8.0
7.5
7.0
6.5
6.0
5.5
5.0
4.5
4.0
3.5
3.0
2.5
2.0
1.5
1.0
0.5
0.0
-0.5
f1 (ppm)

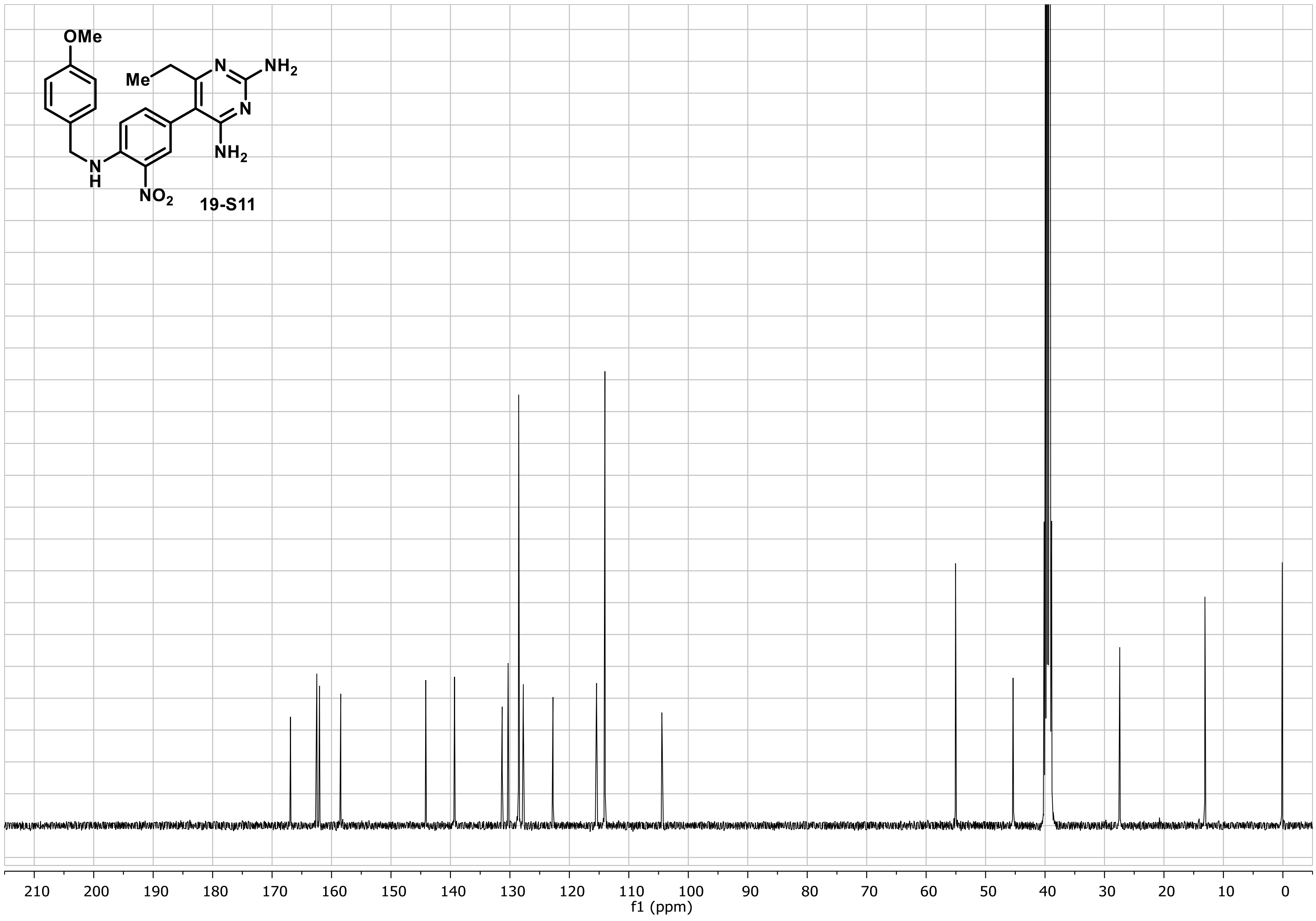
OMe
Me
N
NH2
N
NH2
N
H
NO2
19-S11
210 200 190 180 170 160 150 140 130 120 110 100 90 80 70 60 50 40 30 20 10 0
f1 (ppm)

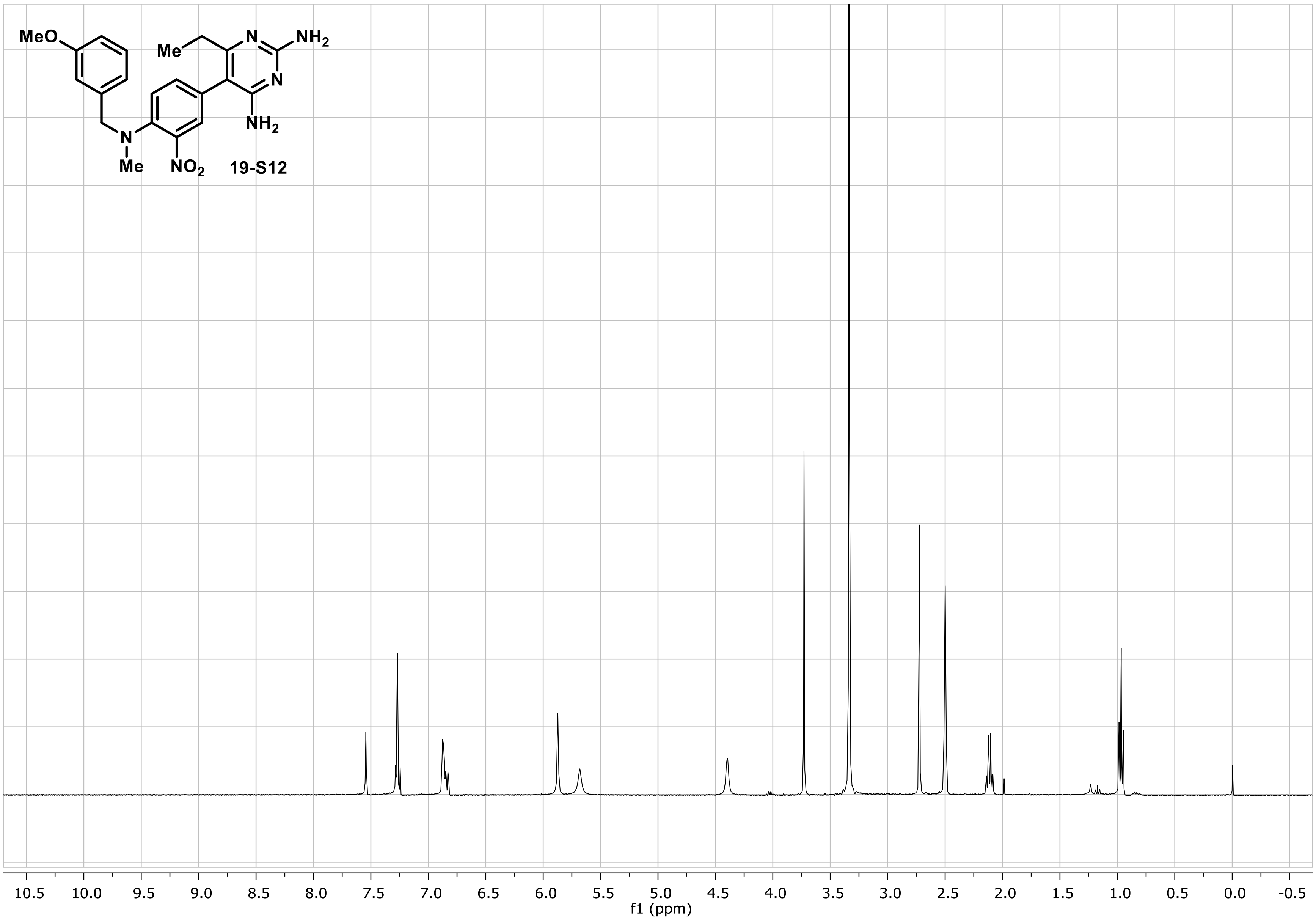
MeO
Me
N
NH2
N
NH2
N
Me
NO2
19-S12
10.5 10.0 9.5 9.0 8.5 8.0 7.5 7.0 6.5 6.0 5.5 5.0 4.5 4.0 3.5 3.0 2.5 2.0 1.5 1.0 0.5 0.0 -0.5
f1 (ppm)

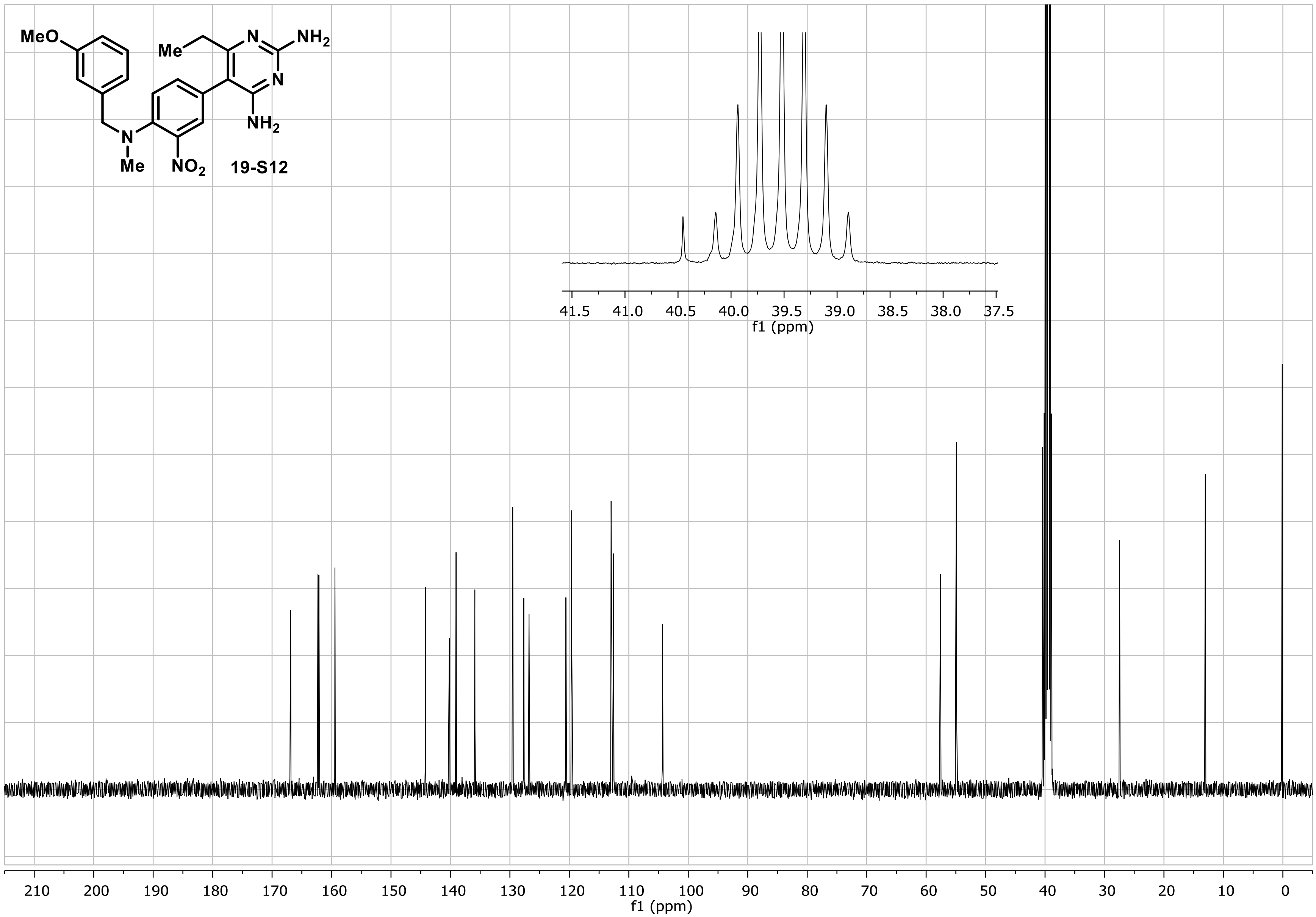
MeO
Me
N
NH2
NH2
N
Me
NO2
41.5
41.0
40.5
40.0
39.5
39.0
38.5
38.0
37.5
f1 (ppm)
210
200
190
180
170
160
150
140
130
120
110
100
90
80
70
60
50
40
30
20
10
0
f1 (ppm)

19-S12

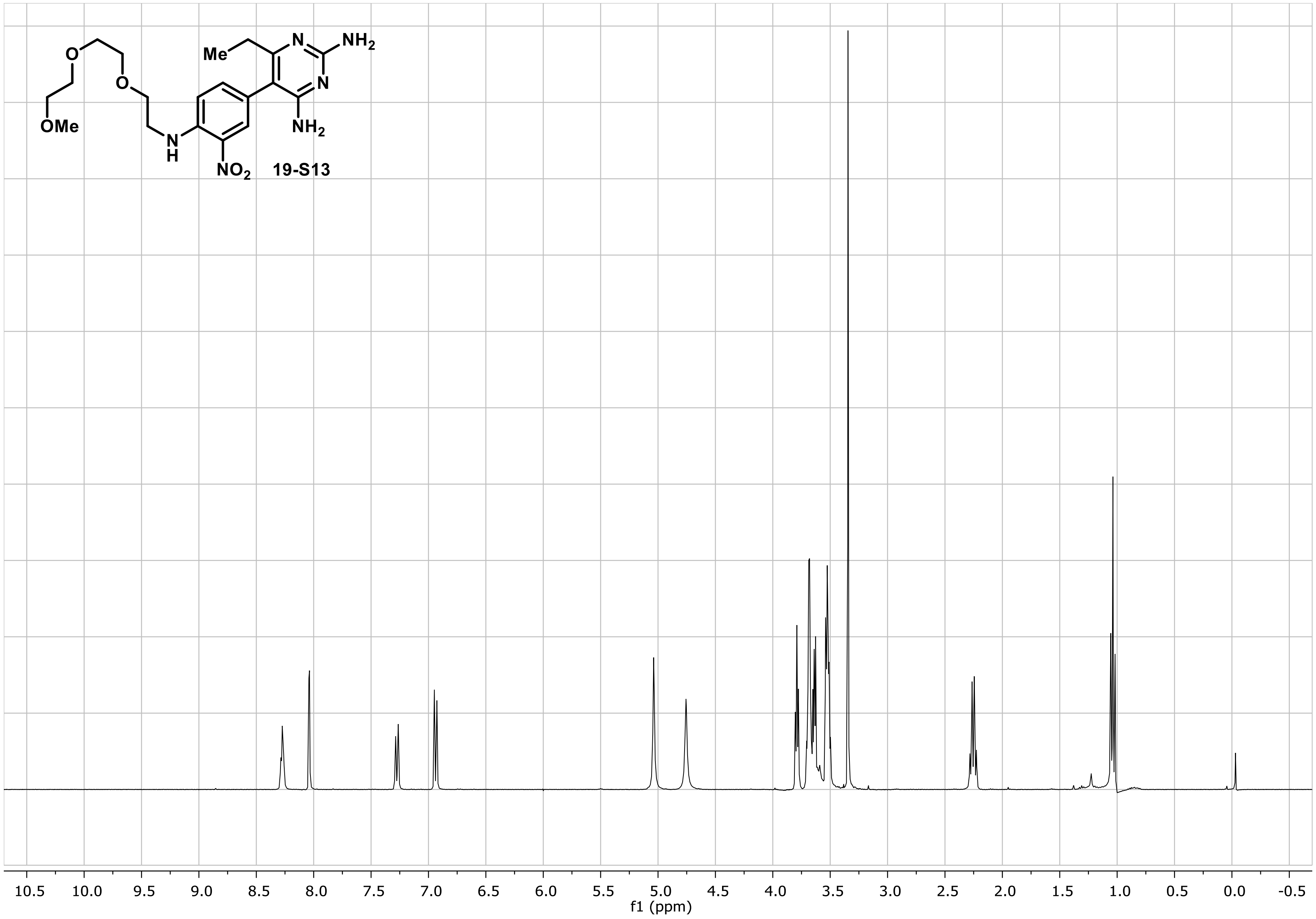
OMe
O
O
N
H
NO2
Me
N
NH2
N
NH2
19-S13
10.5 10.0 9.5 9.0 8.5 8.0 7.5 7.0 6.5 6.0 5.5 5.0 4.5 4.0 3.5 3.0 2.5 2.0 1.5 1.0 0.5 0.0 -0.5
f1 (ppm)

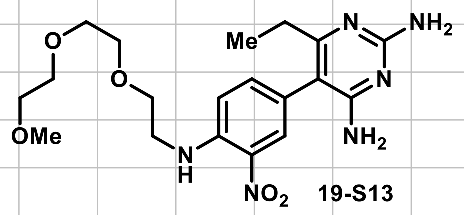

19-S13

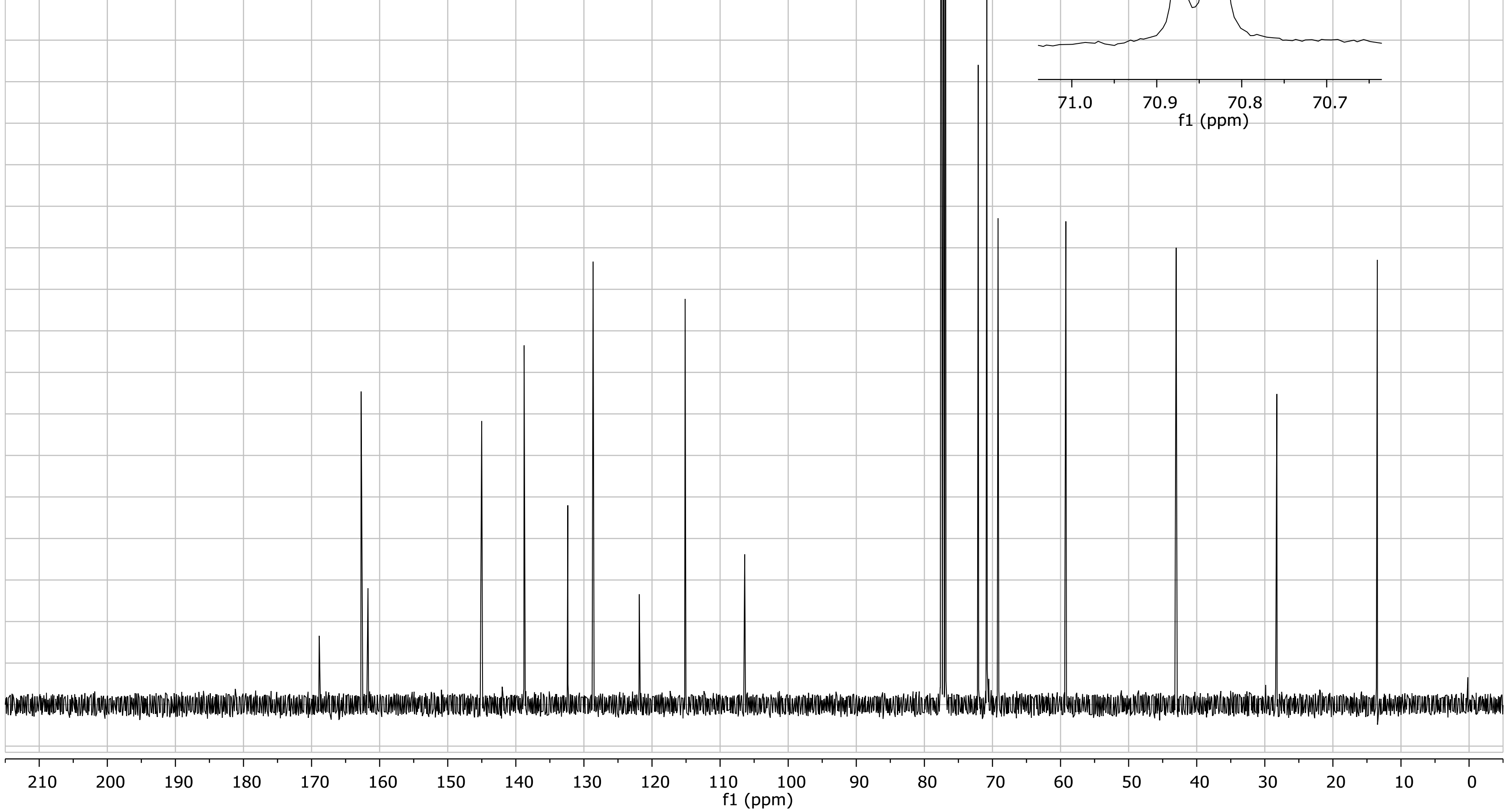

71.0
70.9
70.8
70.7
f1 (ppm)
210
200
190
180
170
160
150
140
130
120
110
100
90
80
70
60
50
40
30
20
10
0
f1 (ppm)

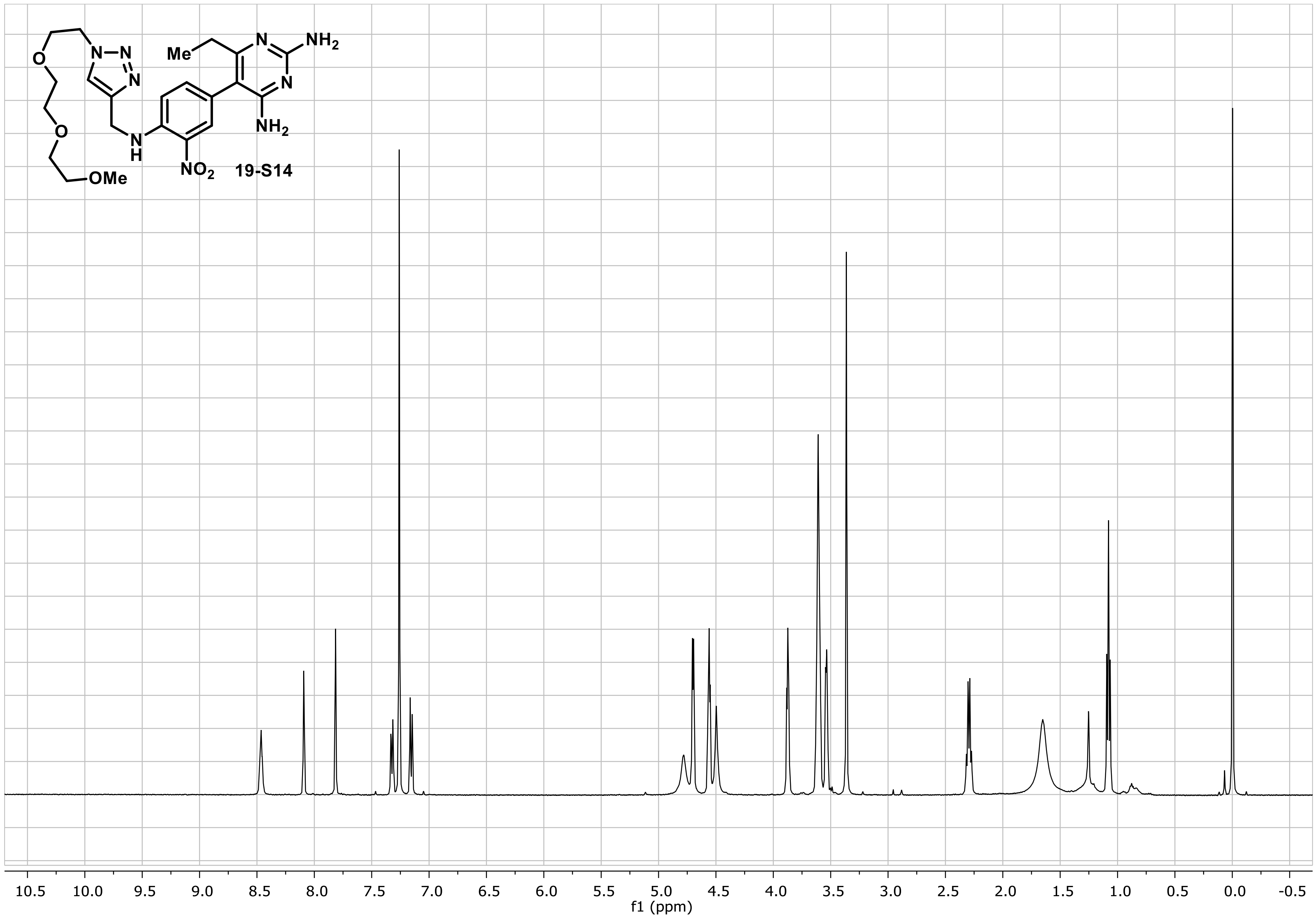
Me
N
NH2
N
NH2
N
N
N
O
O
OMe
N
H
NO2
19-S14
10.5
10.0
9.5
9.0
8.5
8.0
7.5
7.0
6.5
6.0
5.5
5.0
4.5
4.0
3.5
3.0
2.5
2.0
1.5
1.0
0.5
0.0
-0.5
f1 (ppm)

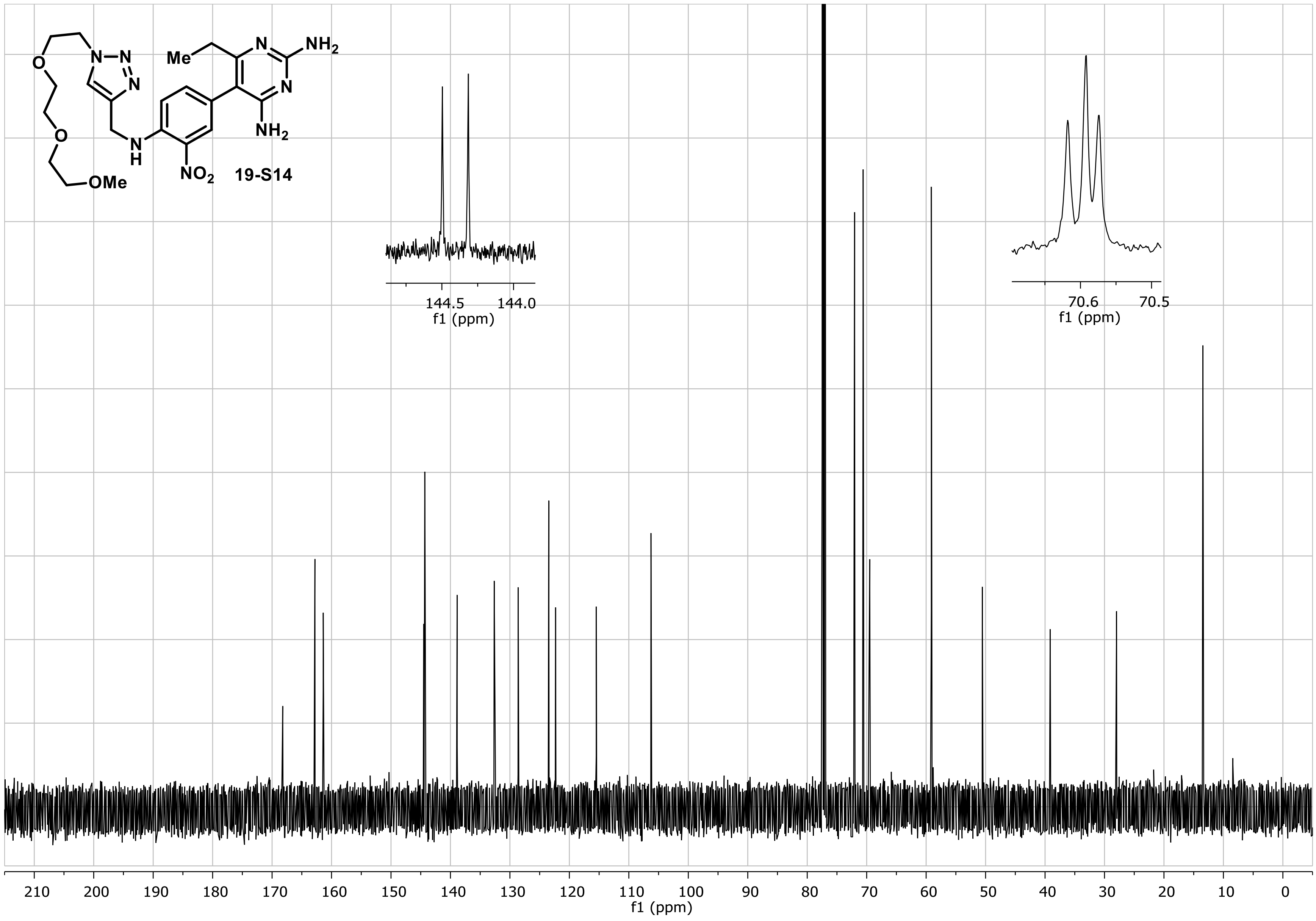

19-S14
144.5
144.0
f1 (ppm)
70.6
70.5
f1 (ppm)
210
200
190
180
170
160
150
140
130
120
110
100
90
80
70
60
50
40
30
20
10
0
f1 (ppm)
